# Misinterpreting Electrophysiology in Human Cognitive Neuroscience

**DOI:** 10.1111/psyp.70303

**Published:** 2026-04-21

**Authors:** Tzvetan Popov

**Affiliations:** ^1^ Department of Psychology University of Konstanz Konstanz Germany; ^2^ Methods of Plasticity Research, Department of Psychology University of Zurich Zurich Switzerland

**Keywords:** action, brain oscillations, brain potentials, cognition, EEG, eye movements

## Abstract

An axiomatic view in contemporary neuroscience is that EEG components such as event‐related brain potentials (ERPs) and oscillations are directly interpretable as manifestations of biological processes that support sensory, motor, and cognitive constructs of interest. This premise justifies and propels research programs in laboratories worldwide, but with a substantial social and economic cost, warranted by the potential for basic‐science discovery and the resulting bench‐to‐bedside transfer for health and disease. But a different premise would be more fruitful. This article proposes that, in psychophysiological experiments relying on vision, EEG components (e.g., P1‐N170, LPP, and alpha oscillations) relate to cognition indirectly through their more direct relationship with oculomotor action. The common experimental design that includes a baseline ocular fixation period preceding stimulus presentation provides an excellent template with which to develop the present proposal. Electrophysiological and eye‐tracking evidence (3 published and 4 new data sets: 7 experiments, N_total_ = 421, in the context of face and affective picture viewing, reading, listening, rest, microsleep and eye movements under closed eye lids), together with simulations grounded in empirically observed oculomotor dynamics, demonstrate how and why common conclusions, and reliance on them in clinical practice/treatment efficacy and drug development studies, are at best premature. Results indicate that the oculomotor system plays a mediating role between such EEG phenomena and cognition. Present evidence supports a complementary view of how EEG can shape the development of a broader thought horizon in psychophysiological theory and practice.

## Impact Statement

In Psychophysiology, it is often assumed that EEG potentials and oscillations directly index cognition. The present work demonstrates instead that canonical signatures (e.g., N170, LPP, alpha‐power) reflect cognitive states indirectly via oculomotor control. Across seven experiments spanning face viewing, affect, reading, listening, rest, microsleep, and eye movements under closed eyelids, this reframing resolves persistent ambiguities and cautions against premature biomarker claims. Integrating eye movements with electrophysiology realigns theory, measurement, and clinical translation.

## 1 Introduction

A century after Hans Berger's seminal discovery, EEG is routinely used today to gauge the mind during work. Measurement of event‐related brain potentials (ERPs) and oscillations is routinely used to encode, decode, and model psychology, and results are interpreted as the manifestation of brain mechanisms that support and implement psychological processes. This at times circular logic is enabled by the boundaries of the accepted set of theoretical and empirical premises that guide the interpretation of the observed results.

The present article makes a case for the role of oculomotor mechanisms as mediators in physiological measurement of cognitive processes. Such work falls squarely within cognitive neuroscience and thus within the broader field of psychophysiology (Marder and Miller 2024). In psychophysiology (whether EEG, MEG, fMRI, pupillography, diverse cardiovascular measures, etc.), the assumption that under controlled experimental conditions the measured signal reflects the neural manifestation of mechanisms that allow direct inference of cognition justifies the use of EEG in psychology, with the expectation of uncovering the set of rules and relationships within and between brain circuits that enable the mind.

What does EEG measure? Dare we ask this question given the profound evidence of a century of work cementing the empirical observation that, irrespective of the sensory domain and experimental paradigm boundaries, the P1‐N170 ERP complex is generated upon stimulus presentation in the time domain, or followed by power modulation of alpha oscillations in the time‐frequency domains?

For the remainder of this narrative, I invite the interested reader to accept the premise that across species the central nervous system evolved to support the hosts' overt action in the environment. This premise is not new. Motor action precedes, accompanies, and follows visual processes as conceived by many influential thinkers in the field (e.g., Lang 1979; Powers 1973; Scheerer 1984). Widely accepted, replicable experiments in the visual domain will serve as examples to evaluate the conjecture that ERP components (P1‐N170) and alpha oscillations primarily reflect oculomotor action, consequent upon which an indirect inference about the manifestation of cognition can be drawn. Yet ERP and oscillation data themselves do not provide direct evidence for this inference, because such data are mechanistically remote from cognitive processes. The present work concerns only the P1‐N170 complex and posterior alpha activity, without arguing that other components or rhythms (e.g., hippocampal theta or motor beta) reflect oculomotor action, although hippocampal theta has been recently shown to be modulated by saccadic eye movements during memory‐guided navigation (Zubair et al. 2026). For reasons that will become apparent below (see also Appendix S1), this conjecture may generalize across sensory domains and relevant psychological constructs.

Most non‐invasive event‐related electro‐ and magnetoencephalographic visual experiments performed since Bergers' seminal discovery have one feature in common. Independent of the research questions and psychological constructs under study, a monochrome background with a fixation target is utilized to record and model a baseline condition. A pervasive assumption is that such a baseline is neutral with respect to the stimulus and any experimental manipulation and hence qualifies as the background activity against which time and time‐frequency data are expressed and subsequently modeled and interpreted. The tacit assumption is that maintaining fixation during baseline is a psychophysiological state (providing associated data) irrelevant to the interpretation of task‐induced data. This assumption is premature; it leads to the misinterpretation, and its rejection will enable a complementary view. The problem with the assumption is that it foregrounds the existence of a truly resting (actionless) condition, during which the literal and metaphorical eye of the participant is resting, and does not provide any useful interpretation of the task data beyond serving as a baseline. Based on the evidence outlined below, I propose that the “fixation baseline” model should be replaced by a framework that recognizes fixation itself as an active oculomotor state with measurable physiological consequences. Rejecting this model does not mean discarding all prior interpretations, but it does require that they be re‐evaluated in light of active visual control during periods previously considered “rest.”

**FIGURE 1:**
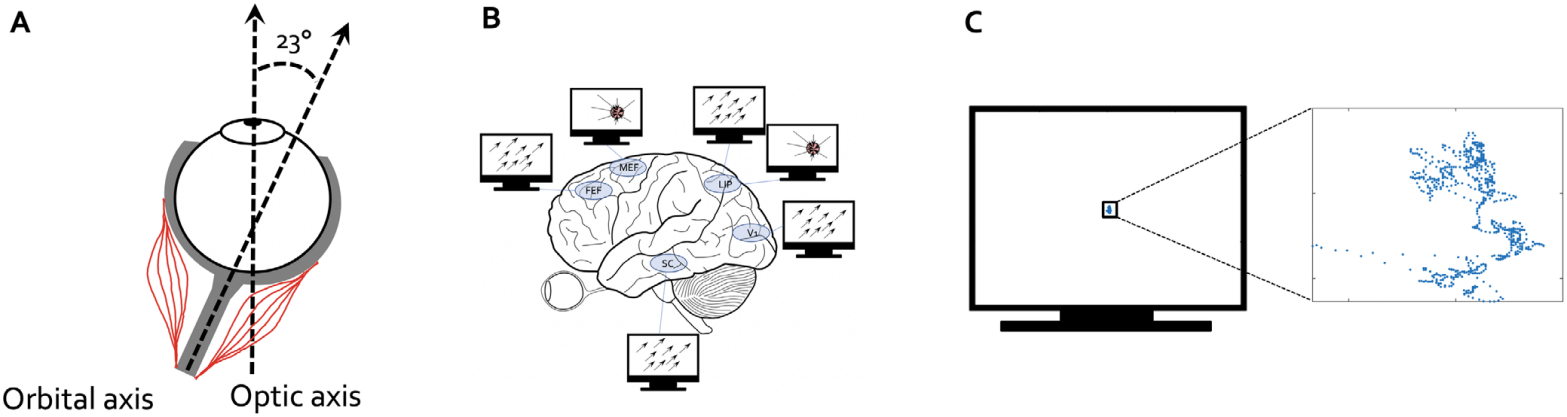
The default state of the eye is movement. (A) Ocular muscles support the continued maintenance of eye position along the optic axis (during the awake state and in the absence of other eye movements such as saccades, vergence, and pursuit). (B) Experiments in non‐human primates utilizing microelectrode stimulation of pyramidal cells in deep cortical and SC layers confirm a coding operation directing the eye along a vector of a fixed size and direction independent of current eye position. This vector code depends on a temporal window of ~100 ms independent of duration or frequency of the electric stimulation. Neurons in the parietal cortex (e.g., lateral intraparietal area LIP in the non‐human primate) and the medial eye field (MEF in the non‐human primate, or the premotor cortex in humans) exhibit also motor‐fields: Independent of the current gaze position, the eye is brought into a particular position on the visual display upon electric stimulation of the corresponding neurons. (C) Maintenance of fixation during cognitive experiments entails miniature, yet preserved ocular action. It is an action typically not noticed in EOG and below a predefined eye movement (e.g., micro‐ or macro‐saccade) threshold but equally engaging and requiring cortical circuit control involved in the manifestation of A and B.

The eye is in constant motion (Martinez‐Conde et al. 2008, 2004; Schiller and Tehovnik 2001). During the awake daytime, the eye is aligned along the so‐called optical axis (Figure 1). However, this position is not resting. Miniature eye muscle contractions maintain a fixation position by continuous movements around a given target (Martinez‐Conde et al. 2008, 2004). These fixational eye movements are instantiated and controlled by the same circuit controlling other types of ocular action such as vergence, pursuit, and saccadic eye movements. The neurophysiological basis of vector coding, microelectrode stimulation, cooling, and ablation experiments in the superior colliculus (SC) and cortical areas is reviewed in more detail in Appendix S1. Its core implication is retained here: invasive electrophysiology in non‐human primates has demonstrated that saccade initiation and fixation control operate within a temporal window of approximately ~100–120 ms (Robinson 1972; Schiller and Stryker 1972; Schiller and Tehovnik 2001), consistent with the fastest saccadic reaction times observed in humans (Kingstone and Klein 1993; Schiller et al. 1987; Schiller and Tehovnik 2001) and peak neuronal bursting in oculomotor structures (Stine et al. 2023; Zhu et al. 2024).

Such intricate neuronal relationships are worth pondering but help little in the interpretation of non‐invasively recorded event‐related potentials and fields. At best, they provide grounds for discussion and interpretation that could easily lead to speculation. And yet, the ~100 ms duration of a P1‐N170 complex is often casually interpreted by researchers as the “neural underpinning” of perception, attention, distractor suppression, and social cognition (e.g., face processing) without eyebrows being raised.

The fact of the matter is, though, that the ~100 ms full‐width half maximum of the P1‐N170 complex is closer to the observations concerning oculomotor action. First, neuronal populations in the cortex utilize a 100 ms temporal window to initiate an eye movement; second, these neurons produce a collective rhythm (i.e., alpha oscillation) with a 100 ms duty cycle, which in turn affects their output and therefore eye movement; and, third, the fastest saccadic reaction time is ~100 ms.

Below, a series of empirical observations are evaluated, derived in the context of widely accepted and utilized psychophysiological experiments such as passive viewing of face stimuli and complex visual scenes, listening to speech, reading, and eye movements under closed eyelids to support the conclusion that ERP components and alpha oscillations often primarily reflect oculomotor action and the neural control systems that support it. Behavior within the scope discussed in the present article is controlled by oculomotor action. The present article does not deny that ERP components and alpha oscillations covary with cognitive states. Rather, it proposes that much of this covariation is indirect. Specifically, some prevailing interpretations are incorrect in assuming a direct mapping between EEG signals and cognitive operations; they are partly correct in identifying reliable associations between EEG signals and task demands; and they are incomplete in failing to account for the oculomotor control mechanisms that systematically mediate these associations. Thus, the present contention is that some interpretations of EEG phenomena do not attend sufficiently to relevant neural control systems driving behavior, do not understand that oculomotor action largely sculpts perceptual sampling, and that these phenomena occur as part of a cybernetic control system. Thus, such interpretations do not accurately attribute the direct role of some EEG phenomena to the correct node or level in the control systems.

## 2 Materials and Methods

### 2.1 Participants

A total of 197 participants were recruited across the three human group studies reported here: the face perception experiment, the picture viewing experiment, and the word reading experiment. These studies were not preregistered. The sample size was limited by resource constraints, such as time constraints related to the duration of the courses within which the experiments were conducted, as well as budget limitations. As argued by Lakens (Lakens 2022), the potential limitations are acknowledged here, given the practical constraints preventing the achievement of an ideal sample size based on power analysis. Participants were recruited from the local university and through community advertisements. The sample comprised 126 female and 41 male participants, with an age range of 18 to 72 years (M = 26.3 years). In the face perception experiment, 55 participants took part (43 female; age range: 20–45 years; M = 25.9 years). In the picture viewing experiment, 84 participated (65 female; age range: 18–44 years; M = 24.7 years). In the word reading experiment, 28 volunteered (18 female; age range: 18–72 years; M = 31.9 years). In the eye movements under closed eyelids experiment, 30 participants took part (8 male; mean age = 21.0 years, SD = 1.73). Prior to participation, all participants provided written informed consent in accordance with the Declaration of Helsinki. All participants reported normal or corrected‐to‐normal vision and no history of neurological or psychiatric disorders. The studies were approved by the local ethics committee at the host institution. Participants received either monetary compensation or course credit for their participation.

Additionally, publicly available data were used. Details regarding the procedures for the story listening experiment (featured in Figure 14) can be found in (Armeni et al. 2022), information about the resting‐state data and procedures is available in (Popov, Gips, et al. 2023; Popov, Trondle, et al. 2023), and details on the microsleep data are available in (Hertig‐Godeschalk et al. 2019; Skorucak et al. 2019).

### 2.2 Acquisition Setup and Procedures

In all experiments, visual scenes, faces, or words were displayed on a SyncMaster P2770HD monitor with 1920 × 1080 pixel resolution. Participants were seated in front of the monitor at an approximate distance of 70 cm, positioned in a chin rest. Stimuli were presented using PsychoPy (Peirce et al. 2019) on a full‐screen gray background.

EEG data were acquired using the mBrainTrain Smarting device, a wireless 24‐channel EEG system. The system utilizes semi‐dry/saline‐based Ag/AgCl electrodes embedded in a cap, adhering to the International 10–20 System of electrode placement. Electrodes were referenced online to the FCz electrode, with AFz serving as the ground. Impedance for each electrode was maintained below 10 kΩ. Data were acquired at a 250 Hz sampling rate and converted offline to an average reference for further analysis.

Eye‐tracking data were acquired using a Tobii Pro Fusion eye tracker, with real‐time gaze data transmitted via the Lab Streaming Layer (LSL) protocol. The eye tracker captured left and right gaze points and pupil diameter, streaming data at a 250 Hz sampling rate. Triggers marking stimulus onset were sent via LSL using a separate stream for synchronization of the EEG and eye tracking streams.

### 2.3 Task Design

**FIGURE 2:**
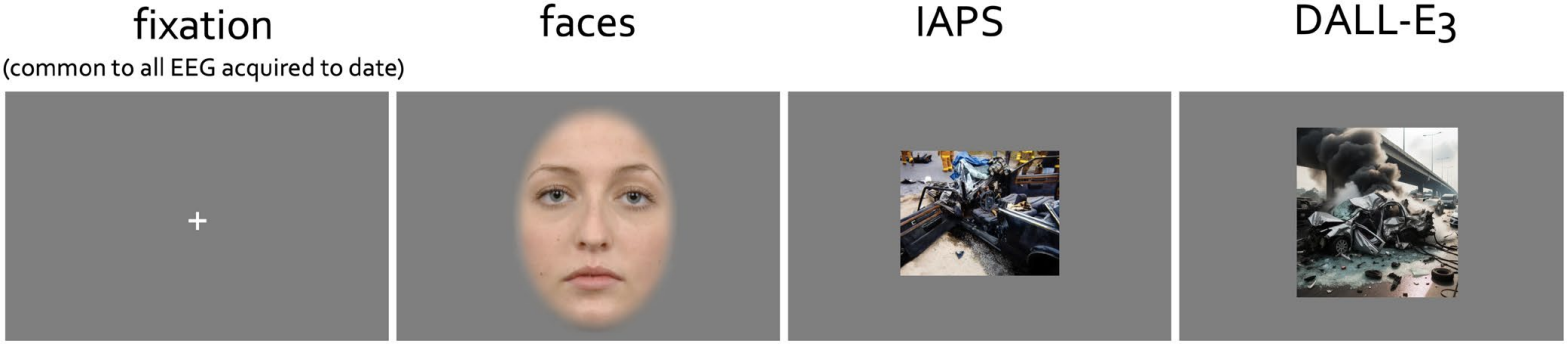
Experiments in human non‐invasive psychophysiology often start with visual fixation on a minimal stimulus. It is used as a baseline and a contrast to all subsequent analyses. In the present narrative examples utilizing human face stimuli, emotionally arousing and neutral images from the IAPS database as well as images generated by artificial intelligence (DALL‐E3) are reported. The duration of fixation viewing across experiments was at minimum 1 s, viewing duration of faces 5 s and of images 1 s. The face image is a stimulus example from the Radboud Faces Database (RaFD, www.rafd.nl).

**FIGURE 3:**
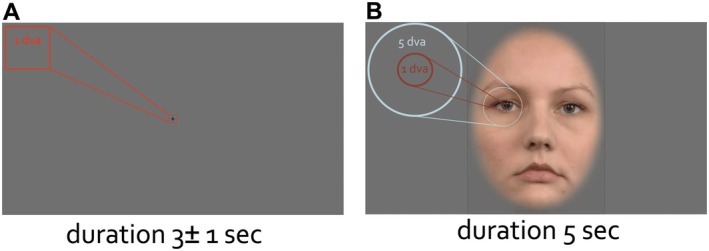
The size of the stimuli at scale. Fixation cross (A) and the face stimuli (B) at the original scale. Participants were positioned at 70 cm distance from the computer screen. The duration of the fixation viewing was at minimum 2 s and face viewing 5 s. The face image is a stimulus example from the Radboud Faces Database (RaFD, www.rafd.nl).

The face task was adopted from previous reports (e.g., Popov et al. 2013, 2014). Briefly, prior to the beginning of the experiment, a fixation cross appeared on the screen for 5 s before the first trial presentation. Each trial consisted of a 5 s video presentation of a face morphing from neutral to a fearful or happy expression of the same actor, or to another neutral expression of a different actor. Participants viewed a randomized sequence of the videos, each belonging to one of three emotion‐based conditions, with 120 trials (40 per condition) presented. Each trial followed the sequence of a baseline fixation cross displayed for 3 s (±0.5 s jitter), followed by the video presentation for 5 s. The experiment continued until all videos had been presented. Valence and arousal aspects of the design have been evaluated elsewhere (Popov et al. 2013, 2014; Popova et al. 2014) and are not further analyzed here. The face images were derived from the Radboud Faces Database (RaFD, www.rafd.nl) including the stimulus examples illustrated here (e.g., Figures 2 and 3).

The picture viewing experiment consisted of multiple trials, with each trial structured as follows. Each trial began with the presentation of a fixation cross (+) on a black background for 5 s before the first stimulus appeared. Participants viewed images categorized into three experimental conditions: 100 pleasant, 100 unpleasant, and 100 neutral. Within the pleasant and unpleasant conditions, half of the images were sourced from the International Affective Picture System (IAPS) database, and the other half were pleasant and unpleasant images generated by OpenAI's DALL‐E 3. All 100 neutral images were from the IAPS database. This manipulation allowed for the examination of LPP effects elicited by images that neither have been systematically rated and evaluated with respect to valence and arousal nor are clearly identifiable as artificially generated. Each image was displayed at the center of the screen for 1 s, followed by a 1 s baseline with a ±0.5 s jitter. Images were randomly shuffled for each session to ensure counterbalancing. In the pleasant and unpleasant condition 50 images were randomly chosen for each subject.^1^

In the word reading experiment, participants completed a passive word reading task designed to elicit natural reading‐related eye movements and associated neural activity. All participants were fluent readers of both English (left‐to‐right script) and Persian/Farsi (right‐to‐left script), with Persian as their native language. Stimuli consisted of visually presented single words in English and Persian. The English word set included nouns, verbs, and adjectives, and the Persian word set consisted predominantly of abstract nouns and adjectival forms. All stimuli were lexical items (i.e., not pseudowords) and were selected to span a broad range of semantic content without requiring explicit affective evaluation. Words were centrally presented, ensuring comparable retinal stimulation across languages and conditions. Participants were instructed to maintain fixation at the center of the screen and to silently read each word as it appeared, without making any overt response. No secondary task or behavioral judgment was required. Each trial began with the presentation of a fixation cross at the center of the screen for 2 s with ±1 s temporal jitter, followed by the presentation of a single word for 1 s. Trials were presented in a pseudorandomized order, and English and Persian words were interleaved across the session. Horizontal and vertical gaze position were continuously recorded throughout the task. Gaze data (see below) were analyzed to characterize reading‐related eye movements. Time‐resolved gaze position was computed relative to stimulus onset, and two‐dimensional histograms of gaze direction were constructed separately for English and Persian word reading. These measures provided quantification of lateralized gaze behavior associated with left‐to‐right versus right‐to‐left reading. By allowing unconstrained eye movements and comparing languages with opposite reading directions, the paradigm enabled the investigation of how early visual and slow cortical potentials relate to the termination of fixation and the initiation of reading‐related eye movements, as well as the lateralization of alpha‐band activity in visual cortex.

To evaluate the present proposal, an eye‐closure and oculomotor control experiment was conducted to examine whether posterior alpha‐band activity reflects tonic oculomotor stabilization rather than eye closure or the absence of visual input per se. The experiment systematically dissociated eye state (open vs. closed) from oculomotor behavior (fixation vs. voluntary eye movements), while holding trial structure constant across conditions. Participants were positioned in a dimly lit room with their head stabilized using a chin rest to minimize head movements and maintain a constant viewing geometry. The experiment comprised 60 trials, each consisting of a 4 s baseline fixation interval followed by a 6 s task interval. Four task conditions were tested (eyes open‐fixation, eyes open‐free eye movements, eyes closed‐fixation, and eyes closed‐free eye movements), resulting in 15 trials per condition per participant. Condition order was pseudo‐randomized such that immediate repetitions were avoided. Instructions for both the baseline and the subsequent task phase were delivered via speakers. During the baseline interval, a central fixation symbol was displayed. In the eyes‐open fixation condition, participants maintained central fixation throughout the task interval. In the eyes‐open movement condition, participants were instructed to move their eyes freely while keeping their eyes open, without constraints on direction, amplitude, or sequence of movements. In the eyes‐closed fixation condition, participants closed their eyes and were instructed to maintain a steady gaze position without moving their eyes. In the eyes‐closed movement condition, participants closed their eyes and executed free voluntary eye movements in the absence of visual input, again without constraints on direction or amplitude. Across all movement conditions, eye movements were self‐paced and unconstrained.

### 2.4 Data Processing and Analysis

Offline data analyses were performed using the FieldTrip (Oostenveld et al. 2011) open‐source toolbox for neuroelectric data analysis. Continuous EEG and eye‐tracking data were segmented around the events of interest (stimulus onsets) into epochs, including a 2 s baseline prior to each trial's stimulus onset.

A finite impulse response (FIR) band‐pass filter (1–45 Hz) was applied to the raw data before artifact rejection. Independent component analysis (ICA) was then used to remove oculomotor and cardiac artifacts. Identified components corresponding to these artifacts were removed, and the cleaned ICA components were projected back into the raw, unfiltered data. After ICA correction, a low‐pass filter (45 Hz) was applied to the reconstructed EEG data, ensuring minimal distortion by 50 Hz mains artifact while preserving low‐frequency neural signals.

EEG data were further inspected for bad channels, which were interpolated using the distance option: electrode locations were aligned to the MNI standard brain using a head model, and neighboring electrodes within 100 mm were identified for interpolation. If any channels were missing from the dataset due to hardware or signal quality issues, they were interpolated based on available neighboring channels using FieldTrip's ft_channelrepair function.

For time‐frequency analysis, power estimates were computed using convolution with a Hanning taper and a fixed window length of 0.5 s, resulting in a 2 Hz frequency resolution for the range 2–40 Hz. The window was stepped every 50 ms across the epoch, covering the range of −2 to 5 s (in the face experiment) and −1 to 1 s (in visual scenes and reading experiments) around stimulus onset.

Power estimates were averaged across trials for each experimental condition separately. A baseline correction was applied using the ‐Inf to −0.25 s pre‐stimulus interval, transforming the power values into decibel (dB) change relative to baseline.

The 1/f aperiodic component was removed from each trial using the *specparam* algorithm, which provides parametrization and visualization of periodic components in the continuous data distinguished from aperiodic activity (Donoghue et al. 2020). Similar time‐frequency analyses were applied to the story listening and resting state/microsleep data, with adjustments made to the epoch length to match the specific temporal dynamics of each condition. The same convolution method was used, ensuring consistency across analyses.

### 2.5 Gaze Data Processing

The raw eye‐tracking data were analyzed by computing 2D gaze density heat maps and eye velocity estimates, providing a comprehensive characterization of visual scanning behavior. For each time point in the respective dataset, gaze positions along the horizontal (x) and vertical (y) axes were selected. To compute gaze density, the data were binned into a 1000 × 1000 pixel grid, and a 2D histogram was created using MATLAB's *histcounts2* function. The raw binned gaze data were then smoothed using a Gaussian filter (*imgaussfilt* with a smoothing factor of 5) to generate a continuous heat map representing gaze density over time. Following 2D transformation, the gaze heat maps were converted into a FieldTrip‐compatible structure with: smoothed gaze density values stored in *powspctrm*, akin to spectral power in time‐frequency analyses, and horizontal positions (time) and vertical positions (freq) as axes in the coordinate grid, ensuring compatibility with FieldTrip's statistical and visualization functions. This transformation allows gaze density data to be analyzed using the same statistical approaches as time‐frequency EEG/MEG data, facilitating direct comparisons between gaze behavior and neural activity.

### 2.6 Eye Velocity Estimation

To examine eye movement dynamics, instantaneous gaze velocity was computed based on the horizontal and vertical eye position signals. The raw data were extracted and smoothed using a 100 ms moving average window (movmean over 25 samples). Velocity was estimated by computing the temporal derivative of smoothed gaze position, scaled by the sampling rate. The resulting absolute velocity values were stored in a FieldTrip‐compatible structure. These preprocessing steps facilitated the integration of gaze position, gaze density, and eye movement velocity into a unified pipeline for statistical comparisons between eye‐tracking metrics and neural oscillations across experimental conditions.

### 2.7 Gaze Dispersion

Horizontal and vertical gaze coordinates were extracted for each trial and analyzed separately during the baseline fixation period and the task viewing period. Samples affected by signal loss (e.g., due to blinks) and gaze positions near the screen borders (±5% margin) were excluded. To reduce blink‐ and tracking‐related artifacts, inter‐sample gaze displacements exceeding 20% of the screen extent were rejected. For each trial, gaze dispersion was defined as the square root of the summed variances of horizontal and vertical gaze position and subsequently z‐scored, providing a measure of the spatial spread of gaze around its mean location. For across subject analyses, dispersion values were averaged across trials within each participant and condition, yielding subject‐level estimates used in subsequent analyses.

### 2.8 Microsleep Episode (MSE) Identification and Scoring

An openly available dataset (Skorucak et al. 2019) was analyzed to examine changes in alpha‐band power associated with eyelid closure during expert‐scored microsleep episodes (MSEs). Microsleep episodes (MSEs) were identified using expert annotations provided with the openly available dataset and were not rescored in the present study. MSEs were originally scored by experienced human raters following established high‐resolution wake–sleep scoring criteria. Episodes were defined as transient sleep‐like events of 1–15 s duration, characterized by a clear slowing of the EEG with dominance of theta activity (4–8 Hz) replacing posterior alpha activity (8–12 Hz), most prominently observed over occipital derivations. In addition to EEG criteria, episodes were required to be accompanied by eye closure of at least 80%, as determined from synchronized face videography, and were typically preceded by reduced or absent eye movements. Scoring was performed using simultaneous inspection of EEG, electrooculography (EOG), and video recordings, and ambiguous segments were resolved by expert consensus. This definition corresponds to microsleep episodes occurring at the transition between wakefulness and stage N1 sleep and has been shown to yield reliable inter‐scorer agreement and high detection performance in subsequent validation analyses (Skorucak et al. 2019).

### 2.9 Statistical Analysis

Statistical significance was assessed using the cluster‐based permutation framework to control for multiple comparisons (Maris and Oostenveld 2007). This method accounts for dependencies across electrodes, time points, and (where applicable) frequency by identifying spatiotemporal clusters of significant effects. A two‐tailed alpha threshold of 0.05 was applied, and statistical significance was determined using 500 permutations.

Relationships between posterior alpha power and gaze dispersion were assessed at the single‐trial level using linear mixed‐effects models with random intercepts for subjects. Condition (baseline fixation vs. task viewing), gaze dispersion (modeled as a continuous trial‐level predictor), and their interaction were included as fixed effects. This approach allowed dissociation of state‐dependent effects (baseline vs. task) from trial‐by‐trial covariation between oculomotor behavior and neural activity while appropriately accounting for repeated measurements within participants.

For evaluation of between‐participant effects, alpha power and gaze dispersion were first averaged across trials within each participant and condition. These participant‐level means were entered into a repeated‐measures analysis of variance with the within‐subject factors *State* (baseline, task) and *Measure* (alpha power, gaze dispersion). This complementary analysis tested whether the two measures exhibited opposing state‐dependent modulations at the participant level.

### 2.10 Data and Code Availability

All data and the code necessary to reproduce present results are available at [https://osf.io/q4mez/].

## 3 Results

In the following, complementary considerations supported by empirical observations are outlined. The mix of results and discussion sections, although atypical for research papers, is intentional to help the reader contextualize how past considerations motivate a re‐evaluation and a complementary view of the results presented.

Reiterating the premise, the default state of the eye is movement, and the purpose of action, including eye movement, is to control perception. During awake states, the eyeball in orbit is aligned along the optical axis, maintained in this position by continuous and ongoing eye muscle activity (Figure 1A) (Wright 2006). Eye movement is supported by vector coding operations of cortical and subcortical (SC) neurons (Figure 1B) (Schiller and Tehovnik 2001), and persists during periods of fixation (Figure 1C) (Martinez‐Conde et al. 2004).

Under this premise, ERP components and oscillations are examined first in the context of passive viewing of faces experiment (Figure 2). After a period of fixation lasting 2–3 s, a dynamic face stimulus, gradually changing expression, is presented for 5 s (details reported in (Popov et al. 2013)) but not necessary for the present discussion. Relevant here is that participants were tasked with maintaining fixation for some duration before evaluating a face stimulus. Participants sat at a distance of approximately 70 cm from the visual display. Given the size of the face stimuli, the fixation cross spanned approximately 1 degree of visual angle (dva) (Figure 3A), and the eyes and mouth sections spanned 5 dva (Figure 3B). These dva values were chosen given that compromised foveal vision results in difficulties in interpreting changes in facial expression (Lerner et al. 2006; Seiple et al. 2013). From the observer's perspective, compromised foveal vision makes it difficult to notice, infer, and label changes in facial expressions. For instance, if only part of the nose falls within foveal vision at 170 ms post face onset, interpreting an aberrant N170 as a marker of social cognition impairment in autism is questionable. The key features needed to infer socially relevant cues, such as the eyes, eyebrows, and mouth, have not yet been explored.

The arrangement of the fixation cross relative to the size of the visual display and the participant's distance was consistent across all experiments reported here.

**FIGURE 4:**
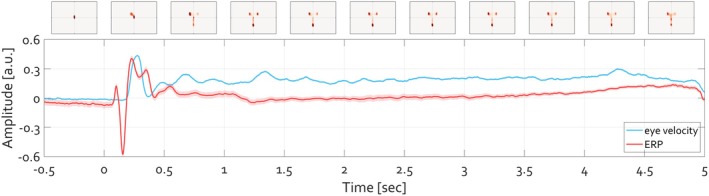
Time course of ERP (red) and eye velocity (blue). After initial fixation interval of 2 s (last 0.5 s shown) a face stimulus was presented for 5 s. The ERP averaged over occipital electrodes O1 and O2 is plotted in red. The shading around that tracing shows the standard error of mean (SEM) across trials (N_trials_ = 2023). The velocity of the eye deviating from the experimentally defined fixation location is illustrated in blue, with the shading corresponding to its SEM. The units (arbitrary unit a.u.) of ERP amplitude and eye velocity are range‐corrected within‐subject in order to facilitate comparison. The position of gaze per 500 ms time windows is illustrated at top, with heatmaps indicate the histogram of eye gaze position‐ the stronger the red color, the more frequent the location of gaze at the respective position.

First, a single participant viewed the presentation of faces (120 trials) over 19 recording sessions, resulting in a total of N_trials = 2023 after artifact correction. Figure 4 illustrates the time course of the ERP averaged across occipital electrodes O1 and O2, along with the time course of eye velocity extracted from continuously recorded eye‐tracking data. Additionally, the gaze position aggregated across 500 ms intervals is depicted at the top of the corresponding time series.

Descriptively, during the baseline fixation interval, the eye remains positioned at the fixation location defined by the experimenter, with minimal deviation as indicated by the variance of the eye velocity (Figure 4 blue line). Following face presentation, a transient ERP (Figure 4, red line) and an increase in eye movement speed are apparent, continuing throughout the trial. The heatmaps at the top of Figure 4 summarize the 2D histogram of gaze locations visited by the eye averaged over 500 ms intervals. Most gaze locations are centered on the fixation during baseline, whereas during the trial gaze lands predominantly on the eyes and mouth in the face stimulus (Figure 4, top panels).

**FIGURE 5:**
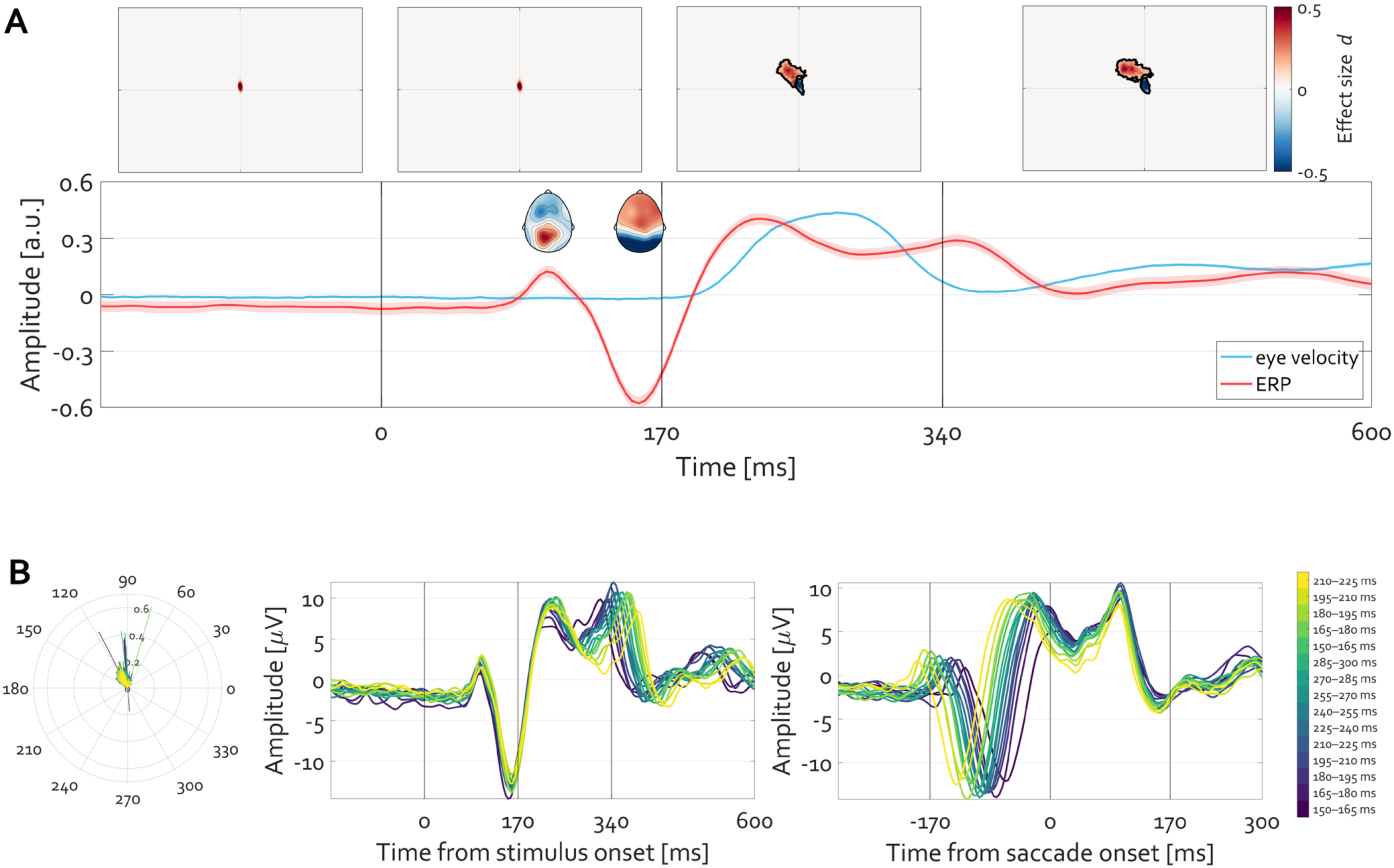
Initiation of eye movement toward face‐specific features of the image follows the P1‐N170ERP complex. (A) The time courses are identical to those reported in Figure 4. The inset topographies illustrate the corresponding topography of P1 and N170, respectively. A statistically significant (cluster permutation test corrected for multiple comparisons, *p* < 0.05) deviation in gaze position is observed after the N170, indicating termination of fixation toward, in this case, the left eye of the actor in the stimulus (red color in the heatmap denoting positive Cohen's *d* effect size). The aggregated position of gaze for the time interval 0 to 170 ms is nearly identical to that extracted from the baseline (−170 to 0 ms). (B) Left: Polar plot illustrating the direction and amplitude of the first saccade, color‐coded by saccade latency bin. Middle: Stimulus‐locked ERPs averaged separately for each latency bin reveal a systematic shift in the timing of the P1‐N170 complex, with earlier saccades associated with earlier ERP peaks and later saccades with later peaks. Right: When the same data are re‐aligned to saccade onset, ERP waveforms converge across bins, indicating that variability in stimulus‐locked ERP latency primarily reflects variability in the timing of subsequent saccade initiation.

One can zoom in on the first 600 ms after the stimulus presentation to closely evaluate the time series (Figure 5A). This examination reveals two insights. First, the change in eyeball rotation within the orbit occurs only after the P1‐N170 complex. In fact, the negativity aligns with the latency of N170 (Feuerriegel et al. 2015). Second, the heatmaps quantifying the 2D histogram of gaze positions during the latency period of 0 to 170 ms are nearly identical to those during the baseline period (−170 to 0 ms). Thus, the shift in gaze position away from fixation (blue color in the heatmaps in Figure 5) toward face features (red color in heatmaps in Figure 5) becomes apparent only after the N170 component. Given the boundaries of foveal and parafoveal vision (Figure 3), at the time of what is commonly interpreted as the “face‐selective” N170, it is implausible that the eyes have already foveally encoded social‐cognition‐relevant features such as variations in eye and mouth parts.

Figure 5B extends this analysis by explicitly accounting for trial‐to‐trial variability in the timing of the first saccade following stimulus onset. Given that saccade initiation from fixation does not occur at a fixed latency across trials, trials were sorted according to the onset latency of the first saccade within the 150–300 ms window following stimulus presentation and grouped into latency bins from early to late. This procedure facilitates examination of neural activity as a function of subsequent oculomotor behavior, rather than averaging trial EEG across heterogeneous saccade timings. When EEG signals are time‐locked to stimulus onset and averaged separately for each saccade‐latency bin, a systematic temporal structure emerges. While the early P1‐N170 complex remains relatively stable across bins, later components shift monotonically with saccade onset latency. Trials associated with earlier saccades show earlier post‐N170 activity, whereas trials associated with later saccades show corresponding delays. This pattern is evident at posterior electrodes (O1/O2), where the visual components are maximal, and is preserved across bins spanning the full range of saccade latencies.

Crucially, when the same data are re‐expressed relative to saccade onset (saccade‐locked ERPs), the waveforms align tightly across bins, with almost none of the temporal variability observed in the stimulus‐locked averages. In this representation, the negative deflection typically identified as the N170 consistently precedes saccade onset by approximately 100–150 ms, irrespective of when the saccade occurs relative to stimulus onset. This alignment indicates that the apparent variability in stimulus‐locked ERPs primarily reflects differences in saccade timing, rather than variability in early sensory processing. The saccade‐locked representation thus reveals a tight coupling between the N170 and the initiation of eye movements that is not evident in the stimulus‐locked averages.

Together, these results demonstrate a robust temporal coupling between the P1‐N170 complex and the initiation of the first saccade away from fixation. Importantly, this coupling is observed before any measurable change in gaze position or eye velocity, indicating that the neural activity indexed by the P1‐N170 complex precedes the execution of the eye movement itself.

The EEG scalp topography around the P1 and N170 latency is the basis for inference about the underlying cortical generators and is interpreted as the electrophysiological basis of mechanisms implementing psychological constructs such as early visual perception and feature encoding. However, based on the findings just reviewed, this interpretation must derive from factors (e.g., mental pattern completion) other than a mere reflection of light radiating in straight lines from low‐level features on the screen. I.e., N170 cannot be a downstream manifestation of face perception as is commonly assumed. At a minimum the transition of features in the displayed face stimulus would require that these features be actively visited by the observer's eye. They were not.

**FIGURE 6:**
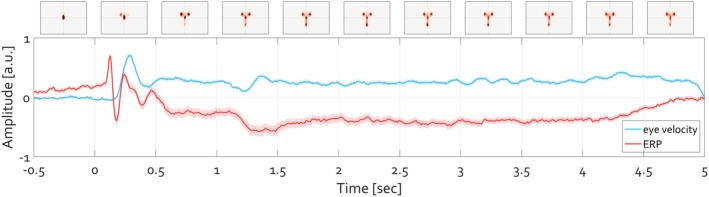
Grand average (N_group_ = 55) of the time course of ERP and eye velocity. As in Figure 4, the velocity of the eye deviating from the experimentally defined fixation location is illustrated in blue, with the shading corresponding to its SEM. The units (arbitrary unit a.u.) of ERP amplitude and eye velocity are range‐corrected within‐subject in order to facilitate comparison. The position of gaze per 500 ms time windows is illustrated at top, with heatmaps indicate the histogram of eye gaze position‐ the stronger the red color, the more frequent the location of gaze at the respective position.

**FIGURE 7:**
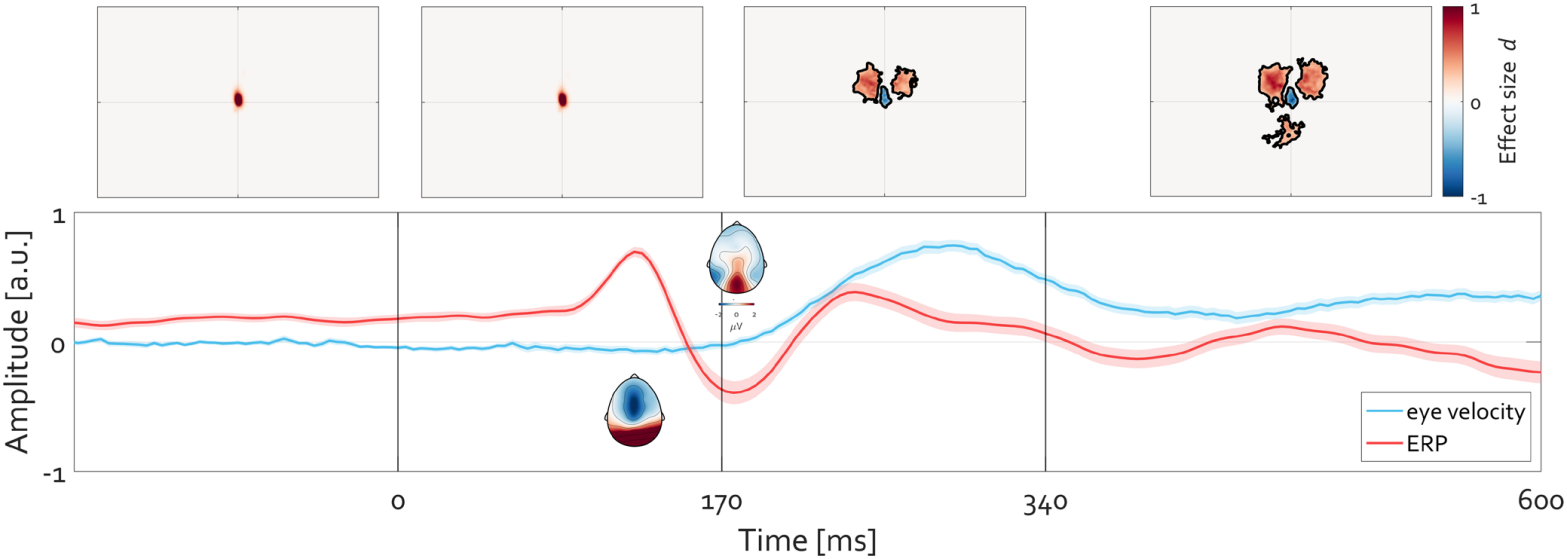
Grand‐average illustration of the initiation of eye movements. On group level (N_group_ = 55), eye movements toward face‐specific features of the image follow after the P1‐N170 ERP complex. Features of the illustration are identical to those of Figure 5A but illustrate the group average rather than a single participant.

This conclusion was replicated at the group level. N_group_ = 55 individuals performed the same task (Figures 6 and 7). Latencies of the early transient ERP components were very similar to those observed in the single individual. Again, a transient increase in eye velocity followed N170 (Figure 6). During the fixation period, the heatmaps depicting the 2D histogram of gaze locations were centered around the center of the visual display and then tracked the contours of a face throughout the trial (Figure 6, top panels). Zooming in on the first 600 ms replicated that, before the P1‐N170 complex, eye movements did not visit the facial features of the actor. Locations corresponding to eye and mouth features were visited by the participant's eyes only evident after the N170 (Figure 7). The topographic difference of the N170 component in the single individual case (Figure 5) versus the group (Figure 7) is not material to this conclusion, as the difference is likely due to variability in position of the standard 10–20 electrode placement relative to variability in the underlying gyrification and anatomy (in the group data) vs. the relatively low variability in the single participant case. Applying the same saccade‐latency‐based analysis at the group level reveals the same temporal relationship between the P1‐N170 complex and subsequent saccade initiation, although with attenuated separation between latency bins due to reduced signal‐to‐noise ratios associated with fewer trials per bin (Figure S1).

Beyond ERP waveforms, the oculomotor account of human alpha oscillations (Popov et al. 2021) emphasizes a relationship between the power modulation of alpha oscillations and the direction of gaze, as well as eye exploration more broadly. Empirical evidence supports the conclusion that higher exploration through eye movements correlates with a stronger decrease in alpha amplitude and vice versa (Popov, Gips, et al. 2023; Popov and Staudigl 2023).

**FIGURE 8:**
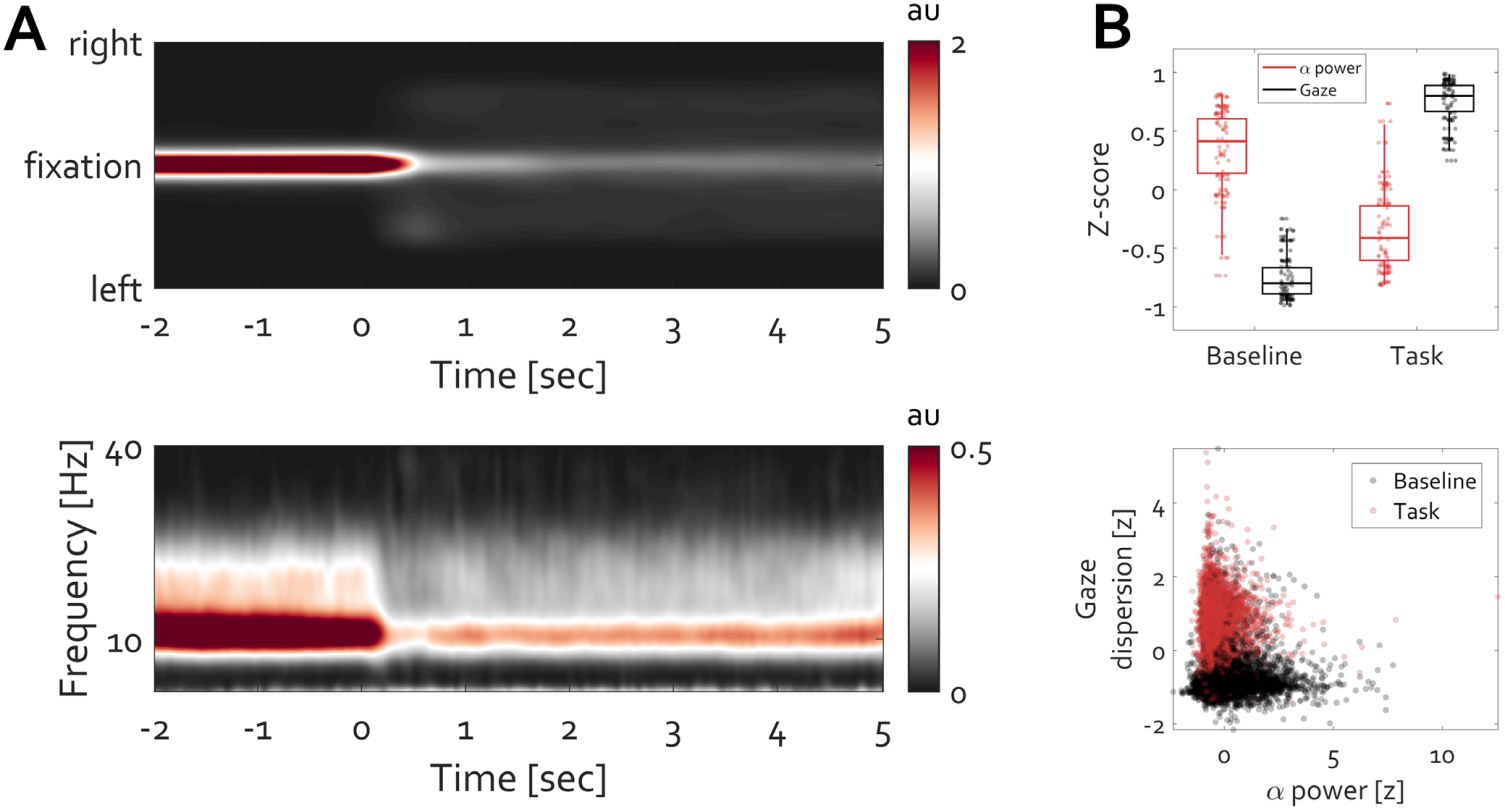
Occipital alpha power modulation and maintenance of fixation are related. (A) Time‐resolved histogram *(top)* of gaze density around fixation. The red color highlights increased density of gaze positioning at fixation. Corresponding time course *(bottom)* of frequency‐resolved activity at electrode POz. Warm colors indicate the strength of oscillatory power. The time course of power modulation corresponds closely to the time course of fixation maintenance. Departure from fixation toward exploring the face stimulus is associated with reduction in alpha power in line with previous reports (Popov, Gips et al. 2023; Popov and Staudigl 2023). (B) Box plots (top) of posterior alpha power and gaze dispersion during baseline fixation and face viewing. Each dot represents the mean across trials for a single participant. **C**orresponding distribution *(bottom)* of gaze dispersion as a function of single‐trial alpha power.

To evaluate the generalizability of this relationship in the context of face viewing, the 2D histogram of gaze positions was computed aggregating over consecutive 500 ms intervals, and a slice approximately ±5° vertical dva around the central fixation was extracted. A sliding window (500 ms) was used to visualize the time‐resolved maintenance and departure from fixation (Figure 8). When juxtaposed with the time‐frequency‐resolved activity averaged over occipital electrodes (O1, O2), the relationship between increased alpha power and fixation maintenance becomes evident (Figure 8A). Gaze behavior and occipital alpha power showed robust condition‐related differences, with task execution associated with reduced alpha power and increased gaze dispersion relative to baseline (Figure 8B). A 2 × 2 repeated‐measures ANOVA with factors State (Baseline, Task) and Measure (Alpha power, Gaze dispersion) tested whether alpha power and gaze dispersion exhibited differential modulation across states in the face‐viewing task. As expected, given range correction of each measure, the main effect of Measure was not significant. A main effect of State (*F*(1,54) = 54.03, *p* < 10^−8^) indicated overall differences between baseline and task periods (Figure 8B). Critically, a State × Measure interaction (*F*(1,54) = 454.42, *p* < 10^−27^) confirmed that alpha power and gaze dispersion were modulated in opposite directions across states. These results indicate a robust state‐dependent dissociation between neural and oculomotor measures during face viewing. To assess whether alpha power and gaze dispersion were coupled at the trial level within these states, a linear mixed‐effects model predicting single‐trial alpha power was fitted with gaze dispersion, State, and their interaction as fixed effects, and random intercepts for subjects. Consistent with the state‐level analysis, this model revealed a strong main effect of State, but neither a main effect of gaze dispersion nor a gaze‐by‐state interaction reached significance (both *p* > 0.84), indicating that trial‐by‐trial fluctuations in gaze dispersion did not linearly predict alpha power within either state.

Descriptively, maintenance of eye position (against the default state) on a baseline fixation target coincides with higher occipital alpha amplitude than with eye exploration during face viewing. As will become clear shortly, leaving this at a descriptive value for now brings a benefit by considering stimuli lacking face features first.

The relationship between eye exploration and evoked brain potentials and oscillations was evaluated in the context of viewing complex scenes. Images from the IAPS database, including 100 neutral, 50 pleasant, and 50 unpleasant images with high arousal ratings, as well as 50 pleasant and 50 unpleasant images generated by artificial intelligence using DALL‐E3, were utilized (image IDs and ratings of IAPS images as well as the AI images used are accessible via https://osf.io/q4mez/). Participants viewed a total of 300 images presented pseudo‐randomly for 1 s each. The baseline period was jittered between 1 and 2 s, during which participants were instructed to maintain central fixation.

**FIGURE 9:**
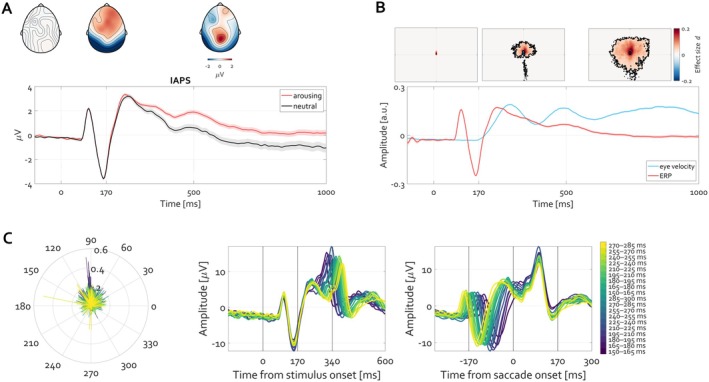
Initiation of eye movement toward features of a visual scene follows after the P1‐N170 ERP complex. (A) ERPs illustrating the initial P1‐N170 complex followed by a slow potential commonly referred to as the late positive potential (LPP), commonly larger for arousing than neutral images. The topo maps above the waveforms illustrate the corresponding topography during the baseline, N170, and the difference (arousing minus neutral) for the LPP latency 400–1000 ms post‐stimulus onset. (B) ERPs pooled across both conditions and the corresponding eye velocity. Shading denotes SEM across trials. The heatmaps above the waveforms illustrate the 2D histogram of gaze positions from stimulus onset until 170 ms after (left), the statistical contrast baseline (−170 to 0 ms) vs. 170–500 ms, and the statistical contrast baseline vs. 500–1000 ms post visual scene onset. Statistical testing was carried out with cluster permutation tests corrected for multiple comparisons (*p* < 0.05) expressed in units of Cohen's *d* effect size. (C) Left: Polar plot illustrating the direction and amplitude of the first saccade, color‐coded by saccade latency bin. Middle: Stimulus‐locked ERPs averaged separately for each saccade latency bin reveal a systematic shift in the timing of the P1‐N170 complex, with earlier saccades associated with earlier ERP peaks and later saccades with later peaks. Right: When the same data are re‐aligned to saccade onset, ERPs peak around 90 ms, i.e., converge across bins, indicating that variability in stimulus‐locked ERP latency primarily reflects variability in the timing of subsequent saccade initiation.

The single participant evaluated in Figure 5 also viewed these images (300 in total from the IAPS database only) across 24 sessions, resulting in N__trials_ = 5496 after artifact correction. Figure 9A illustrates the ERP averaged across parieto‐occipital electrodes (‘P3’, ‘Pz’, ‘P4’, ‘POz’). Following the initial P1‐N170 complex, a positive slow wave known as the late positive potential (LPP) was observed, which was stronger for arousing images compared to neutral ones (pleasant and unpleasant conditions combined). The topographies depict the distribution of activity during baseline, at the N170 latency, and the difference in topography (arousing minus neutral) during the LPP latency from 400 to 1000 ms post‐stimulus (often defined and examined as a difference wave hence the subtraction) (Cuthbert et al. 2000). This evaluation confirms the well‐documented hedonic arousal effects in the ERP literature (Hajcak and Foti 2020; Schupp et al. 2000).

Examining the time course of eye velocity (Figure 9B), a relationship nearly identical to that observed during face viewing was present. The temporal progression of the P1‐N170 complex was followed by an increase in eye velocity. Heatmaps confirmed that the single individual examined above terminated fixation instructions and began exploring details in the visual scene, yet again only after the N170 latency, very similar to viewing faces (e.g., Figure 5). Applying the same saccade‐latency‐based analysis yielded a closely comparable pattern of results (Figure 9C). Trials were again sorted according to the latency of the first saccade following stimulus onset, and stimulus‐locked ERPs were computed within latency bins. As observed for face stimuli, a systematic temporal structure emerged across bins. While the early P1‐N170 complex remained relatively stable, later ERP components shifted with saccade onset latency, such that bins associated with later saccades exhibited correspondingly delayed post‐N170 activity. Importantly, when the same data are re‐expressed relative to the onset of the first saccade (saccade‐locked ERPs, Figure 9C, right), this apparent latency variability is markedly reduced. In the saccade‐locked representation, the negative deflection typically identified as the N170 aligns closely across bins and consistently precedes saccade onset by approximately 100–150 ms, regardless of the timing of the eye movement relative to stimulus onset.

The presence of this temporal relationship across both face and IAPS stimuli indicates that the close coupling between the P1‐N170 complex and saccade initiation is not restricted to face perception. It may reflect a more general characteristic of active visual viewing under conditions in which fixation is maintained prior to stimulus onset and eye movements are initiated shortly thereafter. Thus, the latency of the P1‐N170 complex is not just correlated with stimulus processing: it is temporally locked to the decision to move and foveate—an action‐perception coupling.

**FIGURE 10:**
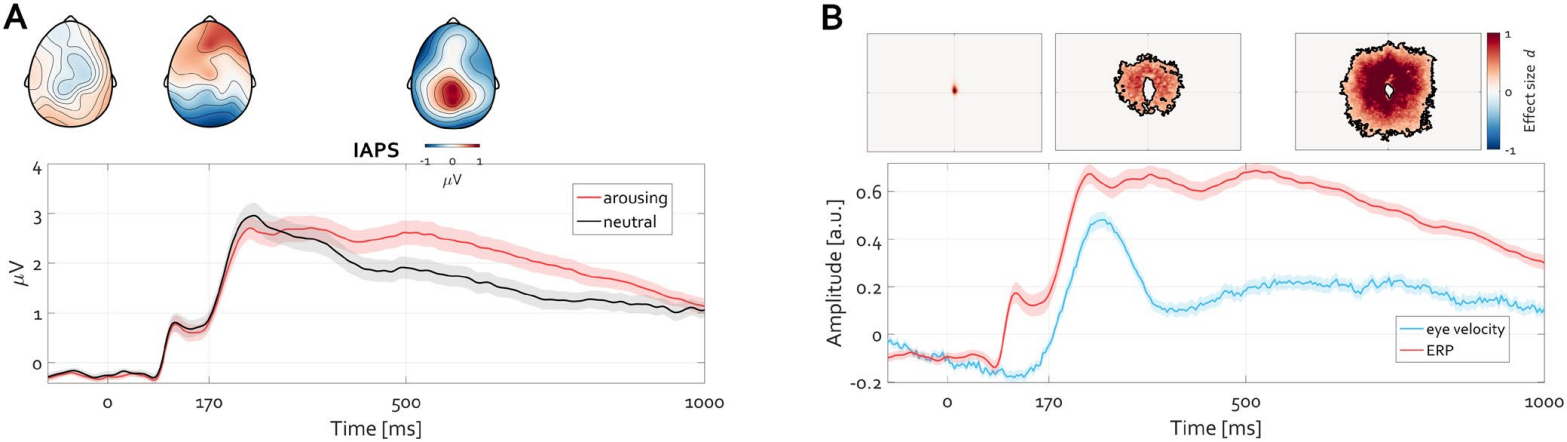
Grand average illustration of the initiation of eye movements following scene presentation. (A) Grand‐average ERP illustrating the initial P1‐N170 complex followed by a slow potential commonly referred to as the late positive potential (LPP‐ stronger for arousing compared to neutral images). The inset grand‐average topographies illustrate the corresponding topography during the baseline, N170, and the difference (arousing minus neutral) for the LPP latency 400–1000 ms post‐stimulus onset. (B) On group level (N_group_ = 85), eye movements toward image features follow after the P1‐N170 ERP complex. The line colors and design of the illustration are identical to Figure 9 but illustrate a group result rather than a single participant.

This relationship was confirmed at the group level (N__group_ = 85). Figure 10A illustrates grand‐average activity across all participants and parieto‐occipital electrodes. The grand‐average time course of eye velocity evolved in parallel with that of the ERP, with the P1‐N170 complex preceding the onset of fixation termination and subsequent image exploration, as indicated by the heatmap contrasts (Figure 10B). Thus, the time courses of the ERP and eye velocity show a clear relationship within individual trials and across participants in both tasks, face and affective picture viewing alike.

The temporal lag between the P1‐N170 complex and the onset of changes in eye velocity is nearly identical during face and picture viewing. However, it remains unclear to what extent the continued exploration behavior (e.g., sustained eye velocity and variability in gaze heatmap compared to fixation) unequivocally relates to the manifestation of slow cortical potentials. This aspect was evaluated next.

**FIGURE 11:**
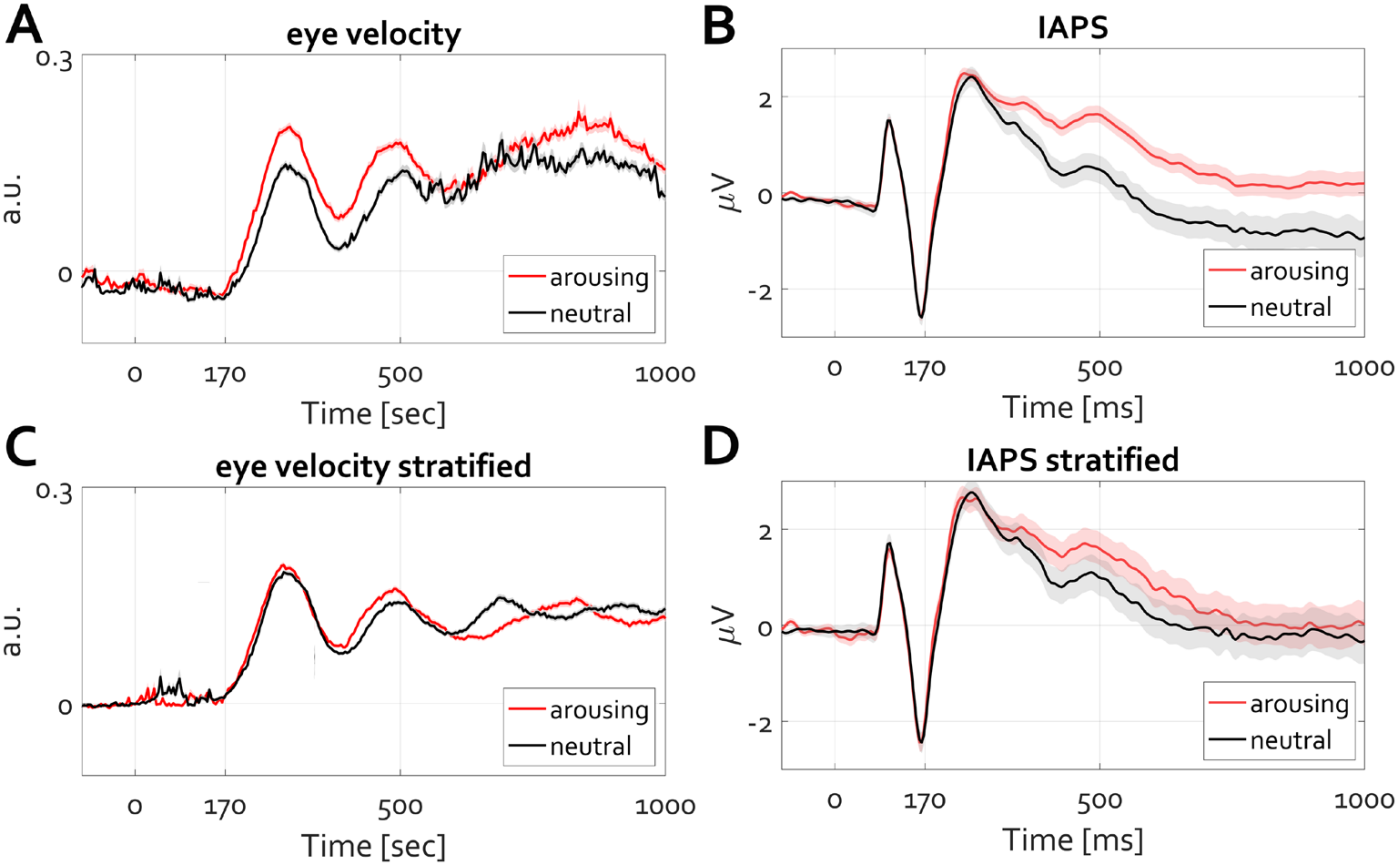
Eliminating condition differences within trials in eye velocity attenuates condition differences within trials in LPP. (A) Eye velocity time course for the arousing (red line) and neutral (black line) conditions. Shading denotes the SEM across trials. A significant condition difference between eye velocity amplitude, larger for arousing than for neutral conditions, is evident. (B) Similar to (A) but illustrating LPP. (C) Eye velocity time course of stratified data highlighting no condition difference in eye velocity (e.g., means across the epoch do not differ). (D) ERP average for the same trials in C demonstrating the absence of condition differences in LPP.

Under the hypothesis that condition differences in eye velocity are independent of differences in slow cortical potentials (commonly assumed but contrary to what this article proposes), the following consideration could be taken into account first. For each participant or trial, the time course of eye velocity (e.g., 400 to 1000 ms) can be averaged to a single value per participant/trial. This results in two distributions of eye velocity (one per condition, arousing and neutral), as depicted in Figure 11A, showing a significant condition difference within a single individual (cluster permutation test, *p* < 0.05). If one could eliminate these condition differences in eye velocity, then under the null hypothesis (advocated here) condition differences in slow cortical potentials (here, LPP) should remain unaffected. To evaluate this alternative, a procedure known as distribution stratification (https://www.fieldtriptoolbox.org/example/stratify/) can be applied. It involves removing values from non‐overlapping parts of the distributions, thereby creating fully overlapping distributions and nullifying condition differences. Although this reduces participant/trial numbers per condition, it prompts the question of whether eliminating eye velocity differences also eliminates LPP differences.

**FIGURE 12:**
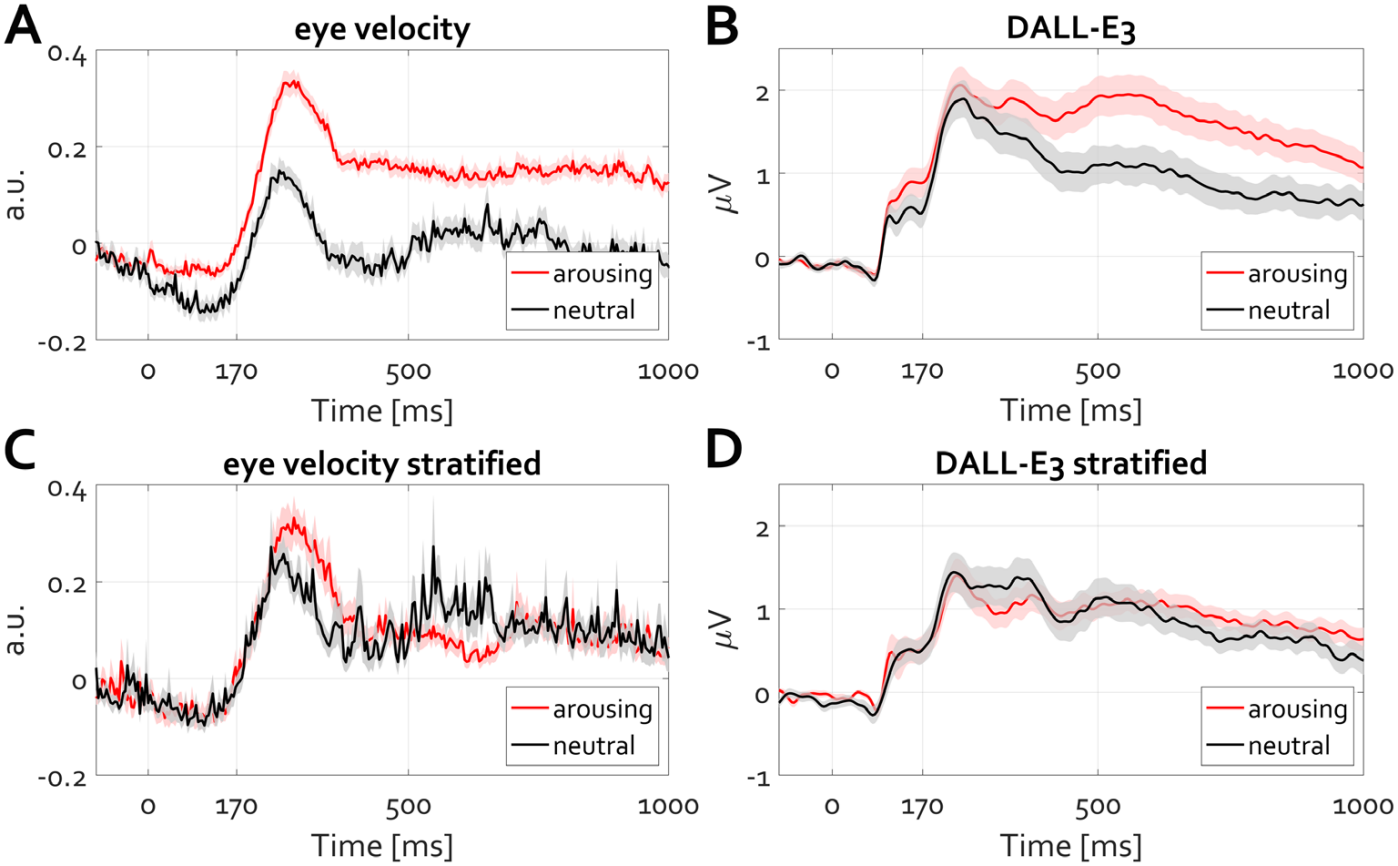
Eliminating group‐level condition differences in eye velocity attenuates group‐level condition differences in LPP. Stratification of the group‐level (*N* = 84) distributions for eye velocity eliminates group‐level (*N* = 25) condition differences in LPP. Figure design and outline identical to Figure 11 but for arousing images generated by artificial intelligence.

The increase in eye velocity following the P1‐N170 complex is notably stronger and more sustained for arousing scenes than for neutral scenes (Figure 11A, cluster permutation test, *p* < 0.05), as are the condition differences in slow cortical potential (Figure 11B, cluster permutation test, *p* < 0.05). Stratifying the distributions of eye velocity by averaging velocity values across the epoch and selecting trials with overlapping distributions diminish condition differences in eye velocity over time (Figure 11C). Although the procedure allows some of the latencies in the time courses to still exhibit significant condition differences, across the entire epochs the means do not differ, consistent with the null hypothesis. For the remaining trials, that is the stratified trials per condition for which there is no condition difference in eye movements, the ERP time courses are shown in Figure 11D. The effect size of condition differences in slow cortical potential is markedly reduced, with overlapping error values suggesting a potential challenge to falsify the null hypothesis, typically in affective psychophysiology, that emotional arousal does not affect cortical responses to affective stimuli. This conclusion, contradicting the predominant view, applies also to group data stratification acquired as a response to artificially generated images, which were neither validated nor rated for arousal and valence. All AI images were easily spotted by the participants for obvious reasons apparent to interested readers (https://osf.io/q4mez/). The primary driver of the LPP effect in AI‐generated images was the mere fact that termination of fixation is followed by eye exploration to evaluate the image (e.g., heatmaps in Figure 10B). In both cases, that is, Figures 11 and 12 the loss of LPP difference supports the view that slow cortical potentials, such as the LPP, covary with increased eye exploration, as measured by the rise in eye velocity from the fixation baseline.

The informative value of analyzing time‐frequency event‐related modulation of ongoing oscillatory activity, although less frequently discussed than in the attention and working memory literature, has gained momentum in affective neuroscience (Codispoti et al. 2023; Flösch et al. 2024; Popov et al. 2012; Schubring et al. 2020; Schubring and Schupp 2019, 2020). The interpretation is that high‐arousing stimulus material is associated with more reduction in alpha/beta power than for neutral material, indicating cortical excitation and engagement of motivational circuits. Less clear, however, is how and why the reduction in alpha/beta power “reflects cortical excitability associated with the engagement of the motivational systems,” as concluded in a recent review on alpha‐band oscillations in emotion (Codispoti et al. 2023). Is this different from, for example, spatial or covert attention, and what does “excitability” actually excite? It cannot be the “motivational system” itself, as the term refers to a psychological construct in its purest form and by definition cannot be reduced to a constellation of excited neurons.

**FIGURE 13:**
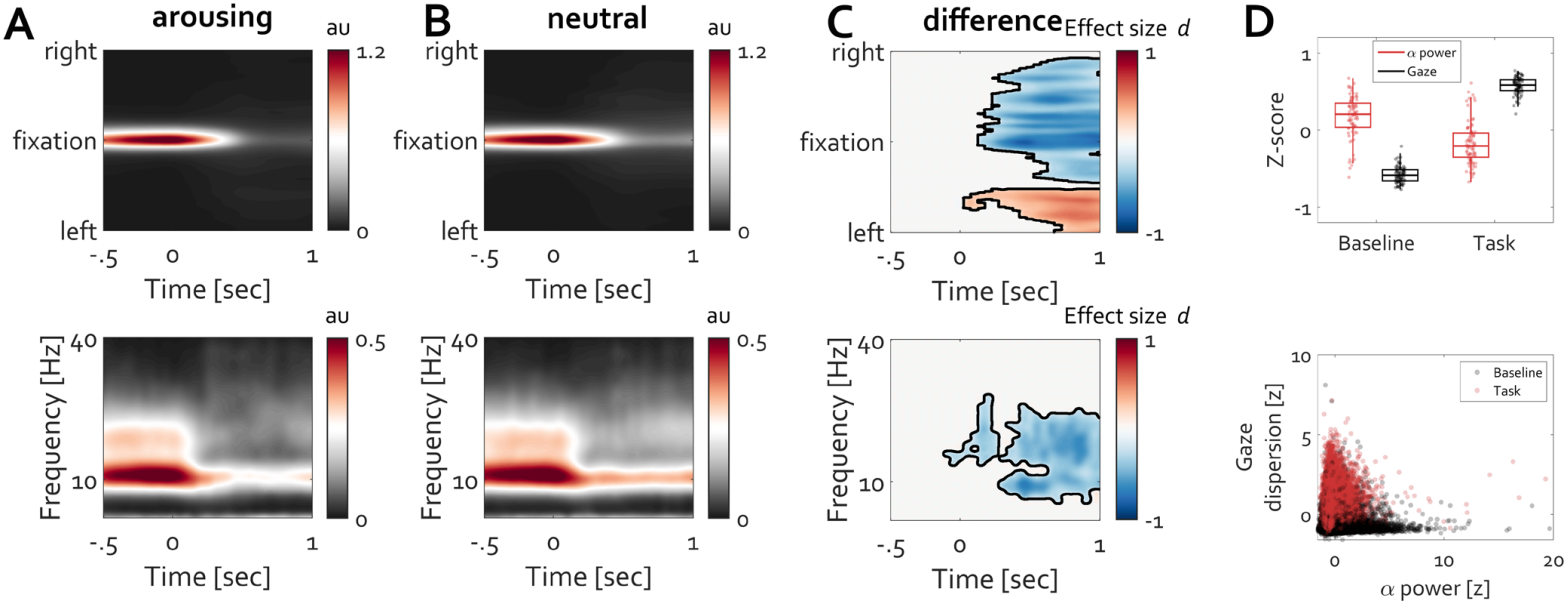
Occipital alpha power modulation and maintenance of fixation are related and inform departure from fixation during image exploration. (A) Time‐resolved histogram *(top)* of gaze density around fixation for arousing images from the IAPS database. The red color highlights the increased density of gaze positioning at fixation. Corresponding time course *(bottom)* of frequency‐resolved activity averaged over electrodes O1 and O2. Warm colors indicate the strength of oscillatory power (in arbitrary units). The primary relevant finding is that the time course of power modulation corresponds to the time course of fixation maintenance. Departure from fixation toward exploring the face stimulus is associated with a reduction in alpha power. (B) Identical to A but for neutral images from the IAPS database. (C) Condition difference (arousing minus neutral) in gaze density *(top)* and alpha power *(bottom)*. Confirmation of the typical reduction in alpha‐beta power yet in association with more exploration for arousing than for neutral stimuli. Cold colors indicate more alpha suppression for arousing then for neutral and vice versa in units of Cohen's *d* effect size. Black contours denote clusters on the basis of which statistically reliable condition differences were identified (cluster‐based permutation test, *p* < 0.05). (D) Box plots (*top*) of posterior alpha power (red) and gaze dispersion (black) during baseline fixation and image exploration. Each dot represents the mean across trials for a single participant. **C**orresponding distribution *(bottom)* of gaze dispersion as a function of single‐trial alpha power.

The present perspective suggests a complementary view. Similar to the contexts of face perception or previously reported spatial attention and working memory (Liu et al. 2022, 2023; Popov, Gips, et al. 2023), as well as episodic memory formation (Popov and Staudigl 2023), the reduction in alpha power aligns with the temporal occurrence of departure from fixation and ongoing image exploration (Figure 13).

During the viewing of both arousing (Figure 13A) and neutral (Figure 13B) images, fixation location is maintained during the prestimulus baseline. Subsequently, exploration of the image using eye movements is initiated, resulting in a decrease in occipital alpha power (Figure 13A,B, bottom). Neutral images are rated neutral not least due to their lower complexity, hence prompting less exploration by eye movements (Figure 13C, top, cluster permutation test, *p* < 0.05). Consequently, there is more reduction in alpha power for more complex arousing images than for less complex neutral images (Figure 13C, bottom, cluster permutation test, *p* < 0.05). This finding is nearly identical to that for covert attention and episodic memory formation (e.g., figure 1B in (Popov and Staudigl 2023)). Here, a 2 × 2 repeated‐measures ANOVA with factors State (Baseline, Task) and Measure (Alpha power, Gaze dispersion) tested whether alpha power and gaze dispersion exhibited differential modulation across states. As expected, given range correction of each measure, the main effect of Measure was not significant. A main effect of State (*F*(1,83) = 161.26, *p* < 10^−20^) confirmed overall differences between baseline and task periods. A State × Measure interaction (*F*(1,83) = 580.51, *p* < 10^−38^) indicated that alpha power and gaze dispersion were modulated in opposite directions across states. Like the results in Figure 8, these results demonstrate a robust state‐dependent dissociation between neural and oculomotor indices. To assess whether alpha power and gaze dispersion were coupled at the trial level, a linear mixed‐effects model was fitted with single‐trial alpha power as the dependent variable, Gaze dispersion, Condition (baseline vs. task), and their interaction as fixed effects, and random intercepts for subjects. This analysis revealed a strong main effect of Condition (*p* < 10^−150^) and a significant Gaze × Condition interaction (*p* < 10^−50^), indicating that the relationship between gaze dispersion and alpha power differed across behavioral states. Specifically, greater gaze dispersion was associated with reduced alpha power during baseline fixation, whereas this trial‐by‐trial relationship was significantly attenuated during task viewing. Random intercept variance was negligible following within‐subject normalization, indicating that the observed effects were driven primarily by within‐subject covariation rather than between‐subject differences.

**FIGURE 14:**
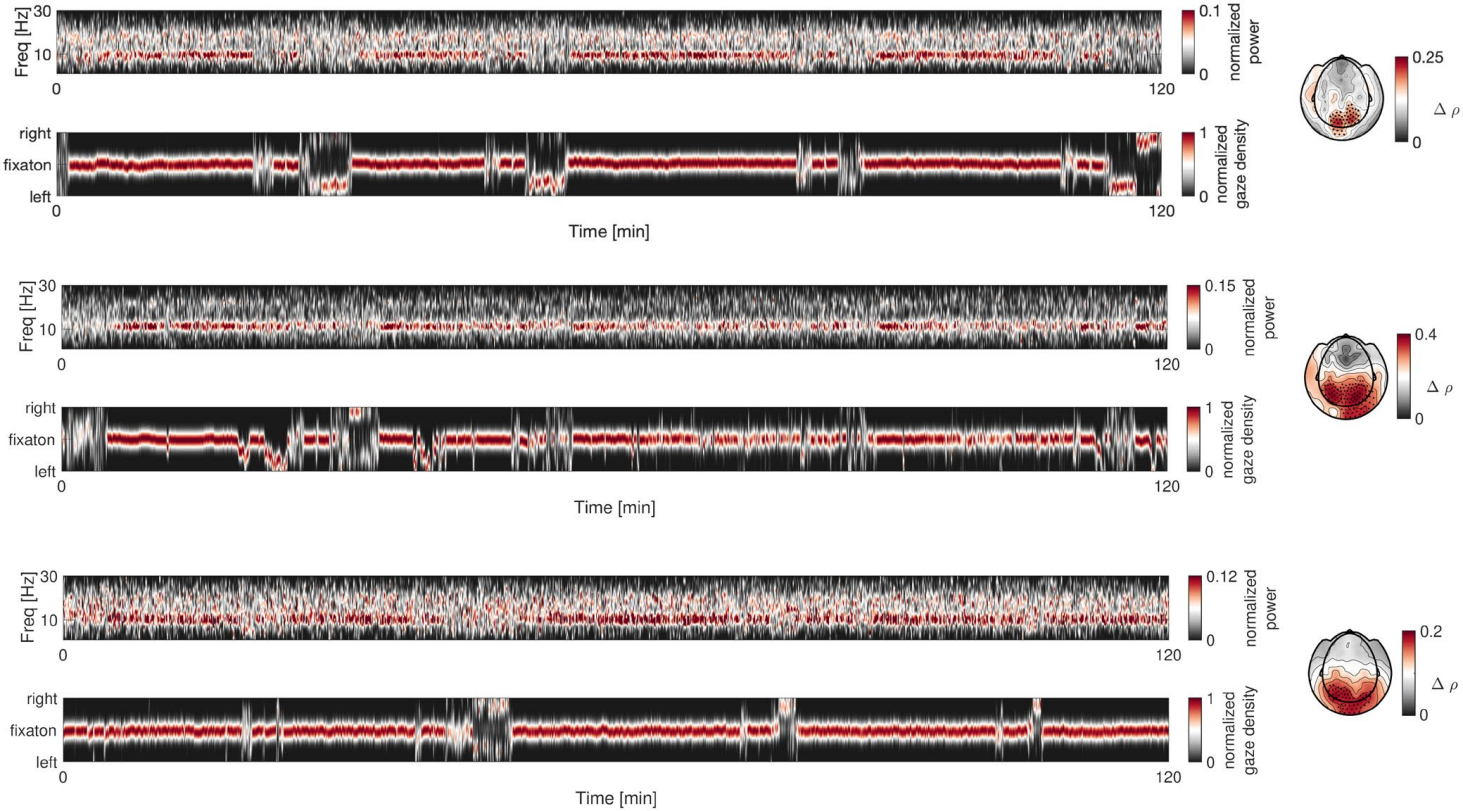
Maintaining gaze direction at fixation displays variations that parallel the modulation of alpha power in the visual cortex across a longer temporal scale. Two hours from a 10‐h dataset are illustrated for each of three participants (top pair of traces, middle pair, bottom pair). Their corresponding time‐frequency spectra and fixation maintenance at the point of fixation are depicted, with the *x*‐axis representing time and the *y*‐axis representing frequency or fixation direction (up for rightward bias, down for leftward). The scalp topographies at right illustrate tests of the correlations (cluster permutation test, *p* < 0.05) between alpha power and variability in gaze density throughout the entire 10 h, contrasted against surrogate data obtained by circularly shifting the time series 500 times.

It is tempting to interpret this power modulation of alpha activity in association with eye exploration within the context of a motivational construct anchored along the approach‐avoidance axis of behavioral disposition. This association is ongoing, present throughout the awake state, as shown in the next analysis (publicly available data; Armeni et al. 2022) of 10 h of recordings of participants listening to audiobooks (Figure 14). Whereas the relationship between alpha power and fixation behavior can be quantified at the trial level in event‐related paradigms, the analysis presented here addresses a state‐dependent coupling that unfolds over extended timescales, necessitating a continuous, time‐resolved approach rather than trial‐wise comparisons. Participants were tasked with listening to stories for 1 h per session over 10 sessions during magnetoencephalography (MEG) and simultaneous eye tracking. Participant‐specific head casts enabled repositioning within the MEG helmet to minimize head positioning errors between sessions to acquire 10 h of data. At random intervals (from the participant's perspective), written text appeared on the visual screen: questions about the narrative details and ensuring attention to the auditory narrative. Figure 14 illustrates the spectral energy of the MEG signal along with the corresponding time‐resolved maintenance of gaze position at central fixation. Termination of the latter corresponds to the occurrence of story‐related questions, necessitating consistent eye movement. Similar to the above results during face and image processing, the time course of variation in posterior alpha activity aligned with maintenance of and departure from fixation. This pattern is evident for all three available participants and was manifested near posterior‐occipital sensors throughout the 10 h (topographies in Figure 14, cluster permutation test, *p* < 0.05).

Several experiments narrated above support the present proposal that EEG components in common psychophysiological experiments relate to cognition indirectly through their more direct relationship with oculomotor action. However, relying solely on the motivational construct in affective picture viewing and face perception does not suffice to infer the asserted specificity of EEG‐derived spectral measures in these contexts. Instead, ocular action and occipital alpha power modulation appear entangled, highlighting the intertwined nature of gaze direction control and occipital alpha oscillations (Popov et al. 2021). The necessity to move the eye in a particular direction along the text reveals the manifestation of this entanglement. Consequent upon this latter observation, reading direction (rightward as in English or leftward as in Farsi, Arabic, and Hebrew) should be dissociable through both gaze direction and occipital alpha power, a hypothesis evaluated next.

**FIGURE 15:**
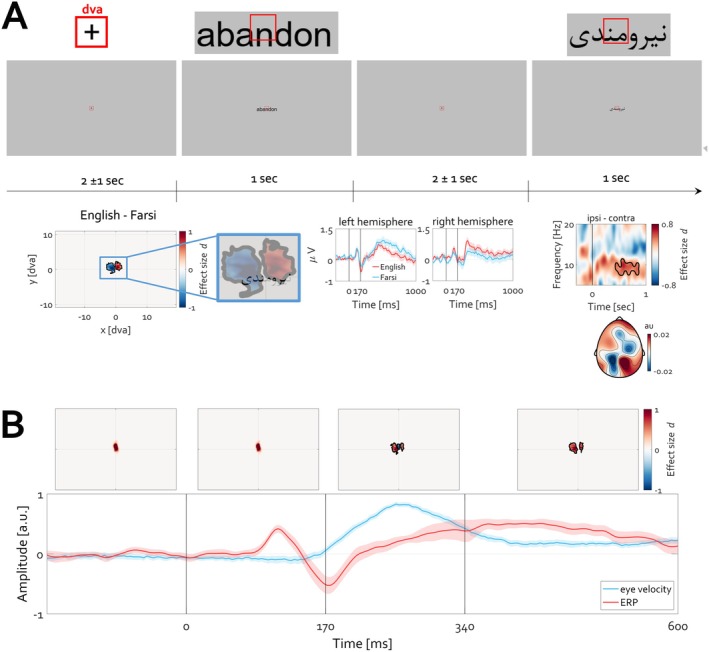
Slow brain potentials and alpha oscillations vary with reading direction. (A) Participants (*N* = 28) capable of reading English and Farsi (mother tongue) words were tasked with silently reading words (pseudorandomly and centrally presented). Following the fixation period of 2 ± 1 s duration, words were presented for 1 s. No task was involved except reading the words. Insets at the top illustrate examples of the size of the words relative to 1 dva. The time course of the experiment illustrated below the insets is at the true spatial viewing scale during acquisition. The contrast between the 2D histograms of gaze direction during English minus Farsi word reading revealed a clear lateralization of reading direction. ERPs averaged across a set of left (T7 C3 P7 P3 M1) and right (T8 C4 P8 P4 M2) electrodes confirmed a clear difference in the amplitude of slow cortical potentials (cluster permutation test, *p* < 0.05). Alpha power was lateralized, with an occipital topography indicating that reading English words entails rightward eye gaze direction and contralateral left decrease in alpha power and vice versa. The contrast ipsi‐ minus contra‐ lateral to reading direction (O1 vs. O2) revealed a significant condition difference illustrated in the time‐frequency spectrogram (the black outline highlights a cluster of time‐frequency activity supporting the rejection of the null hypothesis that alpha power does not depend on reading direction, cluster permutation test, *p* < 0.05). (B) Initiation of eye movement required for reading follows after the P1‐N170 ERP complex. The time courses are identical to those reported in, e.g., Figure 7. A statistically significant deviation in gaze position is observed after N170, indicating termination of fixation (cluster permutation test corrected for multiple comparisons, *p* < 0.05) toward the letters in the word. Warm colors indicate an increase and cold colors a decrease in Cohen's *d* effect size. English and Farsi conditions were pooled. The aggregated position of gaze for the time interval 0 to 170 ms is nearly identical to that extracted from the baseline (−170 to 0). The change in gaze position required for reading happens thereafter and coincides with the slow cortical potential in a way similar to that for faces, IAPS, and DALL‐E3 images.

Participants proficient in reading English (rightward, English skills sufficient to pursue studies at the local university) and Farsi (leftward, their native language) viewed 200 randomly presented words (100 per category, Figure 15A, top). The list of words can be accessed here (https://osf.io/q4mez/). Time‐ and time‐frequency‐domain analyses confirmed the prediction that reading direction entails consistent gaze bias and contralateral modulation of alpha oscillations and slow cortical potentials (Figure 15A, bottom). Also during reading, initiation of eye movement followed the P1‐N170 ERP complex, similar to what was observed for faces and complex images (e.g., Figures 5, 7, 9, and 11).

As a final line of evidence, how does the ubiquitous increase of alpha power during closed eyes relate to the presented set of observations that EEG components in common psychophysiological experiments relate to cognition indirectly through their more direct relationship with oculomotor action?

**FIGURE 16:**
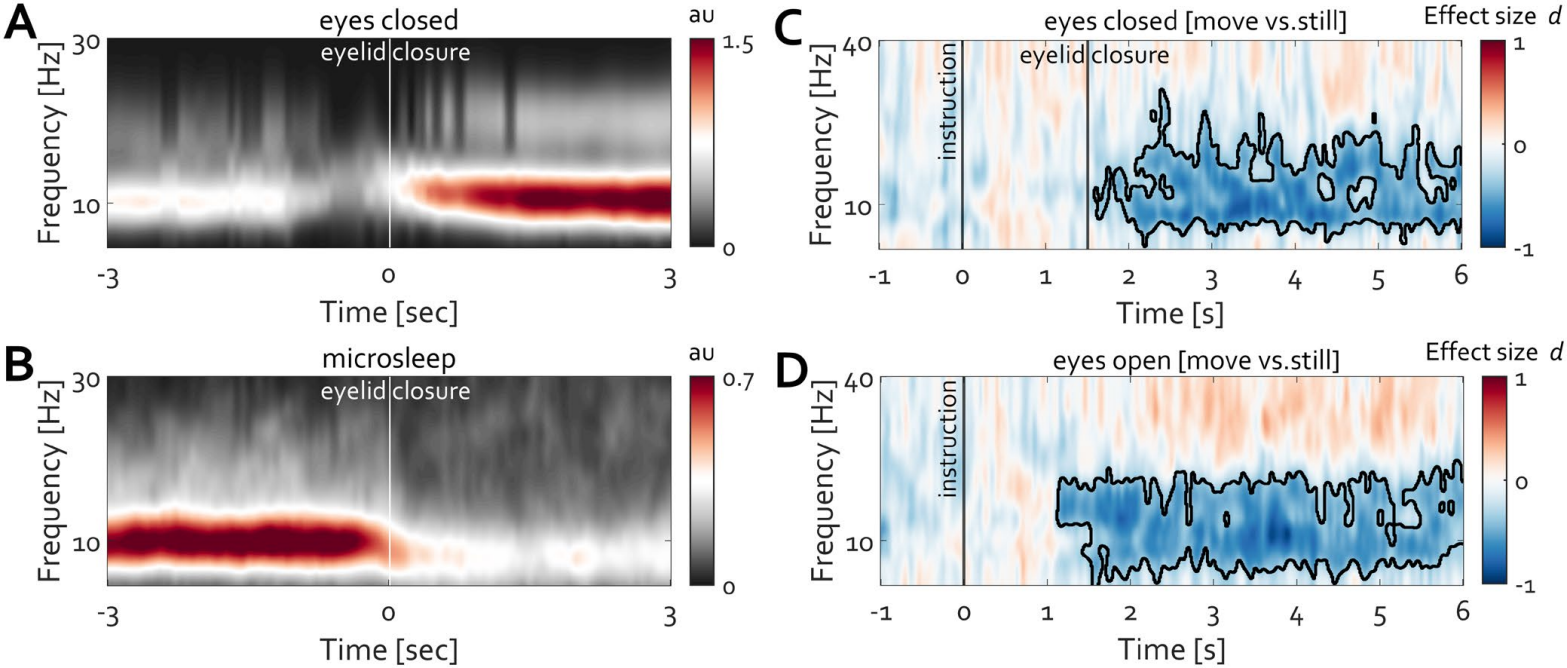
Eyelid closure results in distinct qualitative changes in alpha amplitude modulation. (A) During wakefulness, closing the eyelids leads to an amplitude increase. (B) In contrast, eyelid closure coinciding with the start of a microsleep event diminishes the amplitude of alpha activity. (C, D) Condition difference (move minus still) in posterior alpha power for eyes closed (**C**) and eyes open (**D**). In both eye states, voluntary eye movements are associated with a less alpha power than during fixation. Warm colors indicate an increase and cold colors a decrease in Cohen's *d* effect size. Black contours denote clusters on the basis of which statistically reliable condition differences were identified (cluster‐based permutation test, *p* < 0.05).

Re‐analysis of openly available data evaluating EEG during the eyes‐closed state *N* = 188 (Popov, Gips, et al. 2023; Popov, Trondle, et al. 2023), as well as around microsleep events *N* = 33 (Skorucak et al. 2019), suggests a profound difference despite the identical event (eyelid closure, Figure 16). Instructed closing the eyes increases occipital alpha oscillations (Figure 16A, averaged over electrode locations O1 and O2). The same event, eyelid closure, results in the opposite modulation around a microsleep event (Figure 16B), providing a complementary view of the control of the eyeball in orbit along the optic axis. During awake eyelid closure, the position is actively maintained under closed eyelids. From first‐person experience, the struggle to maintain position along the optical axis before microsleep events is continuously increasing. Subsequent eyelid closure terminates this control, hence the reduction in the rhythm. To directly address whether posterior alpha activity reflects eye closure or, instead, the control of eye position, a control experiment (*N* = 30) orthogonally manipulating eye state (open vs. closed) and oculomotor behavior (fixation vs. free eye movements) was conducted. Under closed eyelids, allowing unconstrained eye movements led to a pronounced reduction in alpha power relative to maintaining stillness (Figure 16C). A similar effect was observed with eyes open, with comparable effect size (Figure 16D). Thus, across both eye states, posterior alpha amplitude was systematically higher during fixation and lower during eye movements.

These findings indicate that alpha oscillations primarily index tonic oculomotor stabilization rather than visual input or eyelid closure per se. Accordingly, the classic eyes‐closed alpha increase reflects the sustained maintenance of eye position along the optic axis during wakefulness, whereas its attenuation during microsleep and voluntary eye movements reflects the release of this control.

## 4 Discussion

The central premise of the present narrative is that a primary purpose of overt action is the control of perception. Concerning vision, the default state of the human eye is movement. Fixation intervals prior to stimulus presentation, treated as neutral baselines independent of the context and sensory modality studied, are a common feature in nearly all psychophysiological experiments involving non‐invasive electrical and magnetic neurophysiology. This task requirement in effect poses a difficult task for the human eye and requires control against its default state. The present report examined a variety of such experiments involving constructs such as social cognition, emotion, reading, and attention. Results foreground the association between both (a) ERP waveform components and power modulation of ongoing alpha oscillations and (b) termination and maintenance of fixation, respectively. As outlined above, the P1‐N170 complex is used here to refer to what is typically known as the N170 in face processing literature and as the N1 in visual attention contexts, including those in which emotion is probed. Traditionally, the use of “P1” and “N170” terms implies distinct meanings. Using the term “P1‐N170 complex” in the present work subsumes them, foregrounding the conclusion that, independent of the study context and its interpretation, the complex informs the experimenter about the impending termination of fixation and the engagement of oculomotor control, rendering problematic the routine reliance on putatively neutral prestimulus baselines in psychophysiological research.

Termination of fixation and increase in eye velocity were found consistently following the manifestation of the P1‐N170 complex across all tasks examined: passive viewing of faces, complex scenes, and reading. Slow sustained cortical potentials were associated with more (and more sustained) exploration of the presented face (e.g., Figures 4, 5, 6, 7), visual scene (e.g., Figures 9, 10, 11, 12), or word (Figure 15), highlighting their close relationship with ongoing oculomotor action enabling elaborate perception and evaluation. Importantly, classic P1‐N170 components observed in passive fixation paradigms are fully compatible with this account, because fixation itself constitutes an actively maintained oculomotor state, and stimulus onset engages oculomotor control and competition processes even when overt eye movements are ultimately withheld.

A critical implication of the present findings concerns how the P1‐N170 complex should be interpreted in relation to eye movements. The present account does not predict that the P1‐N170 complex should be absent when overt eye movements are suppressed. Rather, it predicts that the timing of the P1‐N170 complex should systematically covary with the timing of subsequent saccade initiation. This prediction was directly tested by sorting trials according to the latency of the first saccade following stimulus onset and examining ERPs both stimulus‐locked and saccade‐locked. In stimulus‐locked averages, the latency of the P1‐N170 complex shifted monotonically with saccade onset latency, whereas in saccade‐locked averages, ERP waveforms aligned tightly across bins, with the N170 consistently preceding saccade onset by approximately 100–150 ms. This pattern indicates that much of the apparent variability in stimulus‐locked ERP timing reflects variability in the timing of forthcoming eye movements, rather than independent fluctuations in sensory processing latency.

### 4.1 Visual ERP Components Are Motor‐Visual

The existing and new evidence reviewed here leads to three conclusions about the P1/N170 complex and potentially about other ERP phenomena: (1) direct cognitive attributions of visual ERP components are incorrect as primary explanations; (2) such interpretations remain partly valid as descriptions of downstream effects; and (3) the predominant view in the literature is incomplete without recognizing oculomotor control as the principal driver. The face N170 is arguably just a variant of N1 commonly observed in attention contexts. The N170 component is considered a manifestation of face processing in experiments utilizing face stimuli (Bentin et al. 1996) but is considered an N1 (often assumed to be distinct in terminology and in functional significance) despite also occurring around 170 ms as an attention‐selective component in relevant attention literature (Capilla et al. 2016; Luck 1995; Vogel and Luck 2000). Present results suggest a complementary view of these phenomena and their inferred meaning. The P1‐N170 complex emerges following the instruction to maintain fixation where, upon stimulus presentation independent of the context (face, complex scene, or word), a motor‐visual command is initiated, the result of which is an increase in eye velocity. Although observed topography may vary due to other, temporally overlapping processes and ERP manifestations, it is the fixation instruction common to all of these tasks that provides a common explanation of why and how P1‐N170 modulation is reliably observed across cognitive tasks and constructs studied. Notably, the early timing of the N170 relative to foveation raises questions about its interpretation as a purely stimulus‐driven response. Converging evidence shows that P1‐N170 amplitude and topography are systematically modulated by saccade parameters, including size and direction, and may be more closely linked to oculomotor dynamics than to fixation per se (Amme et al. 2024; Degno and Liversedge 2020; Dimigen and Ehinger 2021; Keren et al. 2010). This perspective suggests that the well‐known enhancement of N170 for faces may, at least in part, reflect the stereotyped scanpaths associated with face viewing rather than stimulus category alone (Dimigen and Ehinger 2021; Johnston et al. 2014; Spiering and Dimigen 2024; Yarbus 2013). A more detailed control‐system interpretation of the P1‐N170 complex, its retinotopic organization, and its relation to fixation‐related potentials and scanpath structure, is provided in Appendix S1.

Consideration of the timing and conduction delays along the early visual pathway provides a mechanistic account for the observed effects (for detailed retinal and subcortical processing, see Appendix S1). Converging evidence from simultaneous ERG‐MEG recordings and fast optical imaging suggests that retinal signals reach primary visual cortex (V1) at approximately 70–80 ms following stimulus onset (Gratton and Fabiani 2010; Westner and Dalal 2019; Westner et al. 2021). From this point on, pyramidal cells in V1 are capable of sending signals downstream along the proposed circuit of saccade generation (Schiller and Tehovnik 2001), including the superior colliculus, brainstem nuclei, and ultimately the eye muscles. Given that the transmission and execution of this motor command requires an additional ~100 ms before an eye movement is observed, one derives a latency of ~170–180 ms, prior to which eye movements following stimulus onset appear unlikely. This estimate is in line with the present results and supports the observation that early evoked responses, besides being visual/perceptive, are also motor in nature—directing both attention and gaze toward active exploration of the environment. Put simply, observer‐generated action is in charge of perception.

### 4.2 Amplitude Modulation of the Late Positive Potential (LPP) Varies With the Intensity of Exploration via Oculomotor Action

To reiterate, the purpose of overt action/behavior is the control of perception. According to the bio‐informational theory of emotional imagery (Lang 1979), the perceptual response itself results from an efferent motor program as a prototype for overt behavior. The theory was a foundational view instrumental in the development of databases such as the International Affective Picture System (IAPS) for the systematic study of emotion, including but not limited to EEG. Consequently, the LPP prompted by IAPS stimuli became among the more widely studied phenomena in affective psychophysiology. The LPP is routinely considered an important electrophysiological manifestation of affective stimulus processing. The presentation of an emotionally arousing visual scene “indicate[s] selective processing of emotional stimuli, reflecting activation of motivational systems in the brain” (Cuthbert et al. 2000). This is a strong statement often interpreted as a solid fact, publicized in textbooks (Gross 2007) and even in clinical trial research as an indicator of and basis for inferences about cognitive‐behavioral treatment efficacy (Garland et al. 2015; Greimel et al. 2020; Zsigo et al. 2024). ERPs are interpreted readily (and in effect directly) as reflecting “cognitive” phenomena (e.g., face and/or affective processing). I argue that this interpretation is incomplete: these components are more parsimoniously explained as manifestations of oculomotor control actions, with cognitive effects arising secondarily. Thus, although common cognitive interpretations may capture downstream consequences, they misattribute the primary generator. Present results suggest a mechanism for how and why the LPP is a robust and replicable metric. Complementary simulations illustrate how oculomotor action may give rise to LPP‐like effects (see Appendix S1). Assuming only (1) a fixed P1‐N170 complex preceding each saccade and (2) variability in exploration rate (high vs. low), the model reproduces, in cross‐trial averages, a canonical stimulus‐locked ERP followed by a sustained slow component whose amplitude scales with the density of oculomotor action. Thus, enhanced LPP amplitudes emerge naturally from increased exploratory eye movements, without invoking sustained, affect‐specific or affect‐enhanced neural responses, reinforcing the interpretation that the LPP reflects oculomotor‐mediated evaluation of visual scenes.

Consequently, emotional stimuli motivate oculomotor exploration (Lang 1979), and eliminating condition differences in eye movements markedly reduces LPP differences at both individual and group levels (e.g., Figures 11 and 12), to the extent that the initially rejected null hypothesis is no longer falsifiable. Moreover, comparable LPP effects are observed for both standardized real‐world images (e.g., IAPS) and artificial images that are neither validated nor perceived as real. This suggests that LPP modulation reflects the extent of oculomotor exploration engaged during stimulus evaluation, rather than the intrinsic affective properties of the stimuli per se, even if such exploration may be triggered by the propositional structure of threat or relevance (Lang 1979).

Hence, the present complementary view is that LPP responses indeed relate to emotion but via their primary relation to eye exploration. Ongoing eye exploration is not specific to affective images but is also present in the case of faces and word reading, where they also co‐vary with slow cortical potentials (e.g., Figures 5, 6, and 15). In support of this conclusion, Kurki and colleagues provide compelling evidence of retinotopic modulation in slow sustained cortical fields (Kurki et al. 2022), mirroring findings in the context of reading presented here. The critical observation is: whether the reading process necessitates eye movements to the left or right, whether an attention probe in the left or right visual field triggers corresponding eye movements, or whether a complex visual scene triggers exploration via eye movements, the phenomenon remains consistent. Common to all scenarios, slow cortical potentials are observed to align with the direction and duration of oculomotor action.

### 4.3 A Complementary View of Alpha Oscillations

The frequency of reports highlighting a relationship between the direction and variability of eye movements and power modulation of alpha oscillations is increasing (Balestrieri et al. 2024; Cruz et al. 2024; Kornrumpf et al. 2017; Liu et al. 2022, 2023; Popov, Gips, et al. 2023; Popov et al. 2021; Popov and Staudigl 2023). These studies consistently report a bias in the direction and variability of microsaccades contralateral to the scalp‐manifested modulation of alpha power. Interpretations and conclusions vary within the scope and boundaries of the construct studied in a given study. Yet, at minimum, the directional bias of microsaccades appears entangled with contralateral alpha power modulation. That is, either informs the state of the other. Based on the results presented here, this bidirectional relationship appears continuous and ongoing: reduction in eye movement to maintain fixation prompts high alpha amplitudes and vice versa. Alpha power reduction is always associated with an increase in eye movements over baseline. This relationship is observed in all cases studied here, independent of task and context, and is evident on different temporal scales—seconds (e.g., Figures 8 and 13), minutes (e.g., figure 3 in Popov and Staudigl 2023), and hours (e.g., Figure 14). What is going on? Can this be understood not just correlationally but mechanistically?

Maintaining fixation requires motor control, which can only be orchestrated by the brain. Hence, it is worth speculating about the signals and mechanisms that enable this control. The present narrative argues that alpha oscillations play a critical role in this process. As mentioned earlier (see also Appendix S1), neurons within the layered structures of the SC, V1, and parietal cortex are equipped with vector‐coding firing capability (Schiller and Tehovnik 2001). This means that the activation of a neuron associated with a specific position will always direct the eye to that position, irrespective of the initial starting point. Consequently, all possible positions are encoded by different neurons in each of these structures, enabling the full degrees of freedom required to explore the environment (within the constraints of morphology, field of view, etc.). Theoretically, when vectors are uniformly distributed across a circular space, each direction is equally likely. For every vector pointing in one direction, there is an equal and opposite vector pointing in the opposite direction. The vector sum of all such pairs cancels out. Given the premise of this narrative—that the default state of the eye is movement—it follows that no single neuron exists solely to maintain fixation at rest, but instead to program a specific direction of ocular movement as demonstrated for example by Schiller et al. (1974) and Schiller and Tehovnik (2001). Yet mammals do fixate, utilizing small fixational eye movements around a given target. If the neurons directing eye movement toward particular directions are uniformly distributed across the visual field and contribute equally to the net vector, their summed activity would cancel out, resulting in a zero vector. This implies no net movement, which could correspond to a neutral, forward‐facing gaze along the optic axis.

A candidate mechanism by which this neuronal recruitment is achieved in the brain is alpha oscillations and their amplitude variation observed in all evaluations examined to date. The amplitude variation and the associated phase of opportunity for neuronal discharge have been reported and discussed in the context of alpha oscillations several times (Chapeton et al. 2019; Haegens et al. 2011; Huang et al. 2021; Popov and Szyszka 2020; Saalmann et al. 2012). Thus, based on present results, one can speculate that an increase in alpha activity, with considerable volume conduction across occipital and parietal cortices, is an emergent event arising from the collective agency of neurons sensitive to different visual field locations, with the phase of the emergent rhythm exceeding the signal‐to‐noise ratio level to an extent that in turn biases their firing properties. The consequence of this is the alignment of gaze along the optic axis. As the occurrence of behaviorally relevant events within the visual field is theoretically unpredictable, in the sense that any location within the visual field is equally possible, positioning the eye along the optic axis provides the best possible subsequent reaction and accuracy in movement toward an upcoming event; that is, it has the most degrees of freedom.

Present data (Figures 8, 13, 14, and 16; and, I suggest, all existing data utilizing fixation during baseline) support this conjecture: periods of fixation during the baseline are always associated with increased alpha amplitude, due to the fixation requirement, and with decreased alpha amplitude during subsequent eye exploration. I am aware of no exception in healthy human non‐invasive psychophysiology. The prevailing interpretation is that the parieto‐occipital cortex is actively engaged in anticipation. But how so? By initiating oculomotor action—that is the mechanism that has been missing in this literature. The generative simulations presented in Figures S4 and S5 provide converging support for this conjecture by demonstrating that increased alpha‐band activity can emerge directly from the dynamics of fixation control, without invoking an explicit oscillatory generator or task‐specific anticipatory mechanism. In the simulation, fixation was modeled as an actively maintained oculomotor state, implemented through repeated, directionally balanced control signals whose net vector average stabilized gaze along the optic axis. Although these control events canceled in the time‐locked ERP due to their symmetric temporal distribution, their regular recurrence produced structured power in the alpha band in the time‐frequency domain. Importantly, this alpha‐band structure emerged solely from the temporal statistics of fixation‐related control signals and disappeared when fixation was released, and exploratory eye movements commenced.

Thus, the simulation shows that alpha enhancement during baseline fixation can also be understood as an emergent consequence of neural‐population‐level oculomotor control aimed at maintaining maximal readiness, rather than as a distinct, stimulus‐independent rhythm serving anticipation per se. This mechanistic account aligns with the empirical observation that alpha amplitude is highest during enforced fixation and attenuated during exploration, and it reframes alpha activity as an index of active perceptual stabilization in the service of future action.

### 4.4 Revisiting Eyes‐Closed Alpha Oscillations: An Oculomotor Stabilization Account

In 1932 Edgar Douglas Adrian reported the presence of alpha oscillations in both insect and human brains, which led him to initially refer to Hans Berger's discovery 8 years prior as the Berger rhythm. Berger declined that honor, and today it is universally known as the alpha oscillation (alpha as the highest observable amplitude, beta as the next highest, and so forth, under certain conditions). The most important conclusion drawn, which dominates current thinking, is that light and its absence are the primary conditions for the emergence of this rhythm, as light off in the insect case and eyelid closure in the human case prompt an increase in alpha power. That interpretation dominates current understanding, resulting in other descriptions such as the “idling rhythm,” serving functional inhibition of cortical circuits and maintaining cerebral connectivity during eyes‐closed rest. Aside from the fact that these interpretations can be derived even in the absence of data, recent observations discuss the presence of a dominant alpha‐like rhythm in the insect brain (honeybee) in relation to sensory action (similar to eye movement control but in the form of sensory antenna movement) (Popov and Szyszka 2020). Amputation of the sensory antenna abolishes this rhythm (e.g., figure 6d in Popov et al. 2021). In humans, congenitally blind individuals who lack visual oculomotor demands and visually guided eye‐movement control show a strongly reduced or abolished posterior alpha rhythm compared to normally sighted healthy controls (Ossandón et al. 2023). That is, not light but action abolishes the rhythm; not darkness but inaction enhances the rhythm.

It follows from this argument that the most compelling case of an increase in alpha oscillations, under closed eyelids, should actually maintain the eye position along the optical axis. In an awake vigilant state, eyelid closure prompts an increase in alpha power with a parieto‐occipital scalp topography (Adrian and Matthews 1934). From first‐person experience, the eyes and gaze direction are aligned along the optic axis upon eyelid opening, but the mechanisms by which eye position under closed eyelids is maintained are a matter of ongoing research (Allik et al. 1981; Ben Barak‐Dror et al. 2024; Bergamin et al. 2002; Iwasaki et al. 2005; Kirchner et al. 2022). The general agreement is related to Bell's phenomenon, a reflex movement associated with a slight upward and outward movement of the eyes that occurs when the eyelids are closed. The present narrative and results presented in Figure 16 favor the conjecture that alpha power increase during closed eyelids supports the maintenance of eye position along the optic axis, ensuring the readiness to act and see upon eyelid opening. That is, starting from the best possible position with a maximum degree of freedom, given uncertainty about the imminent optic flow. This starting position is straight ahead for the same reason detailed above. Conversely, when struggling to maintain gaze direction along the optical axis, as is the case in situations leading up to microsleep events, the alpha power increase is observed before eyelid closure and not after it, as the latter no longer entails active gaze control maintenance (Figure 16). The results presented in Figure 16C provide evidence that alpha oscillations play a central role in this stabilization process. Specifically, alpha power decreases when eye movements are executed under closed eyelids, mirroring the well‐established alpha suppression observed during eye movements with eyes open. This is further evidence that alpha oscillations are sensitive to oculomotor activity per se, rather than to visual input alone.

In short, the three qualitatively different states of fixation maintenance (during baseline periods of cognitive tasks, maintenance of eye position along the optic axis during awake closed eyelid state, and before microsleep events) are supported by the same cortical phenomenon: an increase in alpha power temporally reduces the degrees of freedom of gaze‐direction‐selective neuronal firing in the oculomotor system.

### 4.5 Consequences of Misinterpreting Electrophysiology in Human Cognitive Neuroscience

The implications of the present framework are conceptual, methodological, and clinical. If prominent EEG components primarily index sensorimotor control states—particularly oculomotor action—rather than cognitive operations per se, then this reorientation has consequences for how experiments are designed, how electrophysiological findings are interpreted, and how such measures are translated into “biomarkers” and clinical intervention targets.

As a conceptual implication, adopting an oculomotor‐mediated framework does not invalidate the extensive body of cognitive EEG research, but it does bring a reinterpretation of its mechanistic claims. Classic effects, such as those interpreted as the face sensitivity of the N170, emotional arousal modulation of the LPP, or top‐down attention control of alpha oscillations, remain robust phenomena. What changes is their explanatory status: rather than reflecting the direct implementation of the cognitive or emotional processes under study, these EEG effects are understood more parsimoniously to index transitions in sensorimotor control that are necessitated by task demands and stimulus properties. Furthermore, treating fixation as a baseline reference embeds unexamined assumptions about actionlessness into time‐ and frequency‐domain analyses. Thus, reliance on baselines must be reconceptualized as reliance on active control states. Adopting such a view retains the empirical value of established EEG findings, but their role shifts from evidence for function‐specific cognitive modules to indicators of how action constrains and organizes perception.

The temptation to interpret ERP components as direct “biomarkers” of complex cognitive *functions—such* as social cognition or emotional *processing—rests* on the assumption that these components uniquely reflect those functions, with important clinical implications. The present framework challenges this assumption by showing that the same electrophysiological signatures are tightly coupled to oculomotor behavior across diverse tasks. As a result, clinical or other interventions targeting these signals may have effects that extend well beyond the cognitive domain that they are assumed to index. Thus, putative endophenotypes (Miller and Rockstroh 2013) must be evaluated with respect to the full sensorimotor system they engage, not only the psychological construct they are proposed to represent. *More broadly, the present framework invites a shift in how EEG is conceptualized within cognitive neuroscience. Rather than serving primarily as a window into abstract cognitive operations, EEG is especially well suited to capturing the temporal organization of sensorimotor control—how action, perception, and internal state are coordinated over time*. EEG signals might therefore be a primary tool for studying the dynamics of control systems that enable cognition, rather than a direct readout of cognition itself.

In line with recent dynamical accounts that treat perception and action as jointly emerging from attractor‐based regulation of sensory input (Spaak 2026), the present contention suggests that prominent EEG components primarily index sensorimotor control states, with cognitive interpretations arising secondarily. Recognizing this does not diminish the relevance of electrophysiology for cognitive neuroscience or clinical application; it sharpens it. By aligning interpretation with mechanism, electrophysiology can more precisely inform theory, experimental design, and translational application (Miller 1996, 2010).

The dominant discourse in modern cognitive, affective, and clinical neuroscience assumes that we know how psychology/biology causation works when we do not … there are serious intellectual, clinical, and policy costs to pretending we do know … crucial scientific and clinical progress will be stymied as long as we frame psychology, biology, and their relationship in currently dominant ways. (Miller 2010, p.716)

In this piece Miller argued for the illuminating power of studying biological phenomena for understanding psychological phenomena, but argued against the reducibility of the latter to the former. The piece foregrounded and called for more awareness of possible misinterpretation of their relationship(s) to reduce serious consequences for public policies and clinical practice. As an example, the N170 ERP component was recently characterized as the “first psychiatric biomarker” in the Biomarker Qualification Program of the United States Food and Drug Administration and on that basis was selected for use in the “enrichment of clinical drug development trials” in children diagnosed with autism spectrum disorder.^2^ Thus, an ERP component has been judged to be an objective measure of social functioning [for a recent discussion see (Floris et al. 2025; Mason et al. 2022)]. The latter is arguably one of the more complex cognitive constructs we study. But social cognition cannot happen so quickly after face stimulus onset. One important implication of the present perspective is that developing a chemical that will target modulation of the latency and/or amplitude of N170 in children with autism spectrum disorder (in hopes, for example, of improving their attention to and interpretation of others' facial expressions) will potentially *also* impact (for better or worse) their reading development (e.g., Figure 15), exploration of the environment (e.g., Figures 9 and 13), and social cognition skills (e.g., Figures 5, 7, and 8). Common rationales for, interpretations of, and reliance on results of drug development and clinical practice/treatment efficacy studies (Kala et al. 2021) are at best premature as bases for routine clinical intervention because the assumed and/or assessed scope of their effects is often too narrow. Evaluating effects just on target symptoms and assessing foreseeable unwanted side effects sample too little of the potential impact space of such interventions. A broader conceptualization of the relevant mechanisms is needed.

As developed above, the occurrence of the P1‐N170 complex informs the experimenter about the termination of fixation during baseline and the direction of the eye toward aspects of the stimulus. The former is not “cognition” itself but the necessary ocular action required to visually explore the latter. This active visual exploration is reflected in the presence of later ERP components and in power modulation of alpha oscillations. Consequently, action, overt behavior, can be understood as having the purpose of controlling perception (Powers 1973), as an overarching principle about what the organism is trying to accomplish.

In closing, I submit the complementary view that, in non‐invasive visual electrophysiology (and likely in other contexts), ERP waveforms and oscillations primarily reflect oculomotor or similar perception‐input‐control actions whose purpose is to control perceptual input, with perception emerging as the consequence of these actions.

## Author Contributions

**Tzvetan Popov:** conceptualization, investigation, funding acquisition, writing – original draft, methodology, validation, visualization, writing – review and editing, software, formal analysis, project administration, data curation, supervision, resources.

## Funding

This work was supported by the Schweizerischer Nationalfonds zur Förderung der Wissenschaftlichen Forschung (SNF) Grant 105314_207580 awarded to TP.

## Conflicts of Interest

The author declares no conflicts of interest.

## Supporting information


**Figure S1:** Group‐level relationship between P1‐N170 timing and saccade onset latency. (A) Face viewing task; (B) IAPS picture viewing task. For both tasks, trials were sorted into bins according to the onset of the first saccade following stimulus onset within the 150–300 ms latency window (color‐coded from early to late saccade latencies). Left panels show stimulus‐locked ERPs averaged within each saccade‐latency bin (electrodes P7, P8, O1, O2 average reference montage), demonstrating a systematic shift in the timing of the P1‐N170 complex as a function of saccade onset latency. Right panels show the same data re‐aligned to saccade onset (saccade‐locked ERPs), revealing convergence of ERP waveforms across bins, with the negative deflection preceding saccade onset by approximately 100–150 ms. For both stimulus classes, this pattern replicates at the group level the single‐subject findings shown in Figure 5 (faces) and Figure 9 (IAPS) in the main text, albeit with reduced separation between bins due to fewer trials in the faces task than in the IAPS task and to lower trial counts for some participants.
**Figure S2:** Stimulus‐locked and saccade‐locked ERPs reveal distinct temporal and topographic signatures of visual–oculomotor processing during passive viewing. Single participant data used in Figure 9 in the main paper. Note different y axes. (A) ERP time course locked to stimulus onset (averaged across 5496 trials), illustrating the canonical P1‐N170 complex followed by a sustained slow potential. The scalp topographies above and below the waveform depict the spatial distribution of the P1 and N170 components, respectively, highlighting their characteristic posterior‐occipital dominance. (B) ERP time course locked to first saccade onset during the 1 s viewing window (average across the 12,382 saccades that occurred in the same 5496 trials), revealing a saccadic‐spike potential (SP) tightly aligned to eye‐movement execution, followed by the P1‐N170 complex. The corresponding scalp topographies illustrate the distribution of the SP at saccade onset, distinct from the post‐saccade‐onset P1 and N170 topographies, which have spatial distributions similar to those of the corresponding topographies when locked to stimulus onset.
**Figure S3:** Temporal sequencing of ERP components within successive saccade cycles during free viewing. Single participant data used in Figure 9 in the main paper N_trials_ = 5496. Note differences in y‐axis scales. (A) Left: Grand‐average ERPs from the same free‐viewing trials aligned to different reference events. When locked to stimulus onset (top), a classic P1‐N170 complex is observed. When the same data are re‐aligned to the onset of the first saccade (middle) and the second saccade (bottom), a comparable P1‐N170‐like response re‐emerges relative to each saccade, despite identical underlying neural data. Notably, when ERPs are aligned to the onset of the first saccade, the stimulus‐locked P1‐N170 complex is displaced into the pre‐saccadic baseline, consistent with the interpretation that stimulus‐locked timing reflects overlap with oculomotor preparatory processes. Right: Distributions of first‐ and second‐saccade latencies relative to stimulus onset. Black dots denote the saccade events included at each analysis stage, and dashed lines indicate the corresponding temporal selection windows, illustrating trial‐to‐trial variability and the nested timing of successive eye movements. (B) ERPs binned by second‐saccade latency with three different temporal alignments. When ERPs are aligned to stimulus onset (top), the P1‐N170 complex appears largely time‐locked to stimulus onset, as expected for conventional stimulus‐locked averaging, and systematic latency differences across second‐saccade bins are not readily apparent. When the same data are aligned to the first saccade onset (middle), a systematic latency shift of the P1‐N170 complex emerges across bins defined by the timing of the *subsequent* (second) saccade. Earlier second saccades are associated with earlier ERP peaks and later second saccades with delayed peaks, replicating the temporal nesting pattern observed when binning by first‐saccade latency (i.e., Figure 9C of the main manuscript). This indicates that residual variability in the ERP reflects preparation for upcoming eye movements rather than the immediately preceding saccade. When ERPs are aligned to the corresponding second saccade onset (bottom), latency differences collapse into the baseline, and post second saccade waveforms converge tightly across bins. This convergence indicates a consistent temporal relationship between the P1‐N170 response and the initiation of each saccade, irrespective of absolute stimulus time.
**Figure S4:** Simulated emergence of ERP components and sustained slow potentials from oculomotor action. (A) Stimulus‐locked ERPs generated by the simulation, shown for increasing numbers of trials (top to bottom: 300, 1000, 5000). A canonical P1‐N170 complex is visible following stimulus onset (vertical line at 0 s; N170 peak at ~170 ms), followed by a sustained slow component that gradually decays as saccade rate decreases over the course of stimulus viewing. As the number of trials increases, the sustained component becomes progressively smoother due to improved signal‐to‐noise ratio, without any change in model assumptions. (B) Condition‐specific stimulus‐locked ERPs for low‐exploration (e.g., neutral; blue) and high‐exploration (e.g., arousing; red) trials, along with their difference waveform (gray). Differences between conditions emerge exclusively in the magnitude of sustained post‐stimulus activity, reflecting increased temporal overlap of preparatory P1‐N170 responses for higher saccade rates. The resulting difference waveform closely resembles a late positive potential (LPP) or a P300‐like component. Vertical lines indicate stimulus onset (0 s) and N170 latency (~170 ms). Stimulus offset/return to fixation at 2 s for all illustrated time courses.
**Figure S5:** Simulated emergence of ERP components and alpha oscillations from oculomotor action. (A) Graphical illustration of the simulation procedure. (B) Condition‐specific stimulus‐locked ERPs (average over 1000 trials) for low‐exploration (e.g., blue) and high‐exploration (e.g., red) trials, along with their difference waveform (gray). Similar to Figure S4. (C) Time–frequency representation of simulated EEG power showing elevated activity in the alpha band (8–10 Hz) during periods of fixation. Alpha‐band structure emerges from the temporal regularity of fixation‐related oculomotor control signals maintaining gaze along the optic axis, and is attenuated during stimulus‐driven exploratory viewing. No oscillatory source was imposed; spectral structure arises solely from the timing statistics of repeated fixation control events.

## Data Availability

The data that support the findings of this study are openly available in osf.io at https://osf.io/q4mez/, reference number q4mez.

## Endnotes

^1^
Image IDs from the IAPS data base were as follows. Pleasant: 1710, 1720, 1722, 1811, 2045, 2058, 2075, 2155, 2160, 2209, 2216, 2345, 2346, 4006, 4007, 4090, 4180, 4220, 4225, 4250, 4255, 4290, 4310, 4320, 4490, 4505, 4520, 4525, 4530, 4533, 4542, 4597, 4599, 4601, 4606, 4607, 4608, 4609, 4610, 4611, 4612, 4619, 4623, 4625, 4626, 4641, 4645, 4651, 4652, 4653, 4656, 4658, 4659, 4664, 4666, 4668, 4670, 4680, 4687, 4690, 4693, 4694, 4695, 4698, 4800, 5470, 5600, 5621, 5623, 5626, 5628, 5629, 5660, 5700, 5825, 7330, 7405, 7499, 8001, 8021, 8030, 8031, 8040, 8041, 8158, 8179, 8180, 8185, 8186, 8190, 8200, 8210, 8260, 8370, 8400, 8420, 8490, 8492, 8496, 8500. Unpleasant: 1033, 1050, 1051, 1052, 1120, 1200, 1201, 1205, 1300, 1301, 1525, 1930, 1932, 2095, 2345.1, 2661, 2730, 2811, 3001, 3010, 3030, 3051, 3059, 3061, 3071, 3080, 3100, 3101, 3102, 3103, 3110, 3120, 3130, 3140, 3180, 3181, 3185, 3195, 3212, 3213, 3215, 3220, 3225, 3266, 3301, 3350, 3530, 3550, 6212, 6213, 6231, 6250, 6260, 6312, 6313, 6315, 6350, 6510, 6520, 6540, 6550, 6560, 6563, 6570, 6571, 8230, 9040, 9042, 9043, 9075, 9160, 9185, 9253, 9301, 9325, 9340, 9410, 9412, 9413, 9414, 9421, 9423, 9430, 9495, 9570, 9571, 9590, 9592, 9594, 9596, 9599, 9622, 9635.1, 9900, 9901, 9902, 9904, 9910, 9920, 9921. Neutral: 2002, 2102, 2191, 2200, 2210, 2214, 2273, 2305, 2308, 2357, 2377, 2381, 2383, 2390, 2393, 2396, 2397, 2411, 2480, 2484, 2493, 2495, 2499, 2512, 2516, 2570, 2595, 2749, 2840, 2870, 2880, 2890, 4233, 5471, 5500, 5520, 5530, 5533, 5534, 5731, 5740, 6150, 7000, 7002, 7003, 7004, 7006, 7009, 7010, 7011, 7012, 7014, 7016, 7017, 7019, 7025, 7026, 7032, 7034, 7035, 7038, 7041, 7043, 7045, 7052, 7053, 7055, 7056, 7057, 7059, 7061, 7062, 7080, 7090, 7100, 7130, 7150, 7175, 7179, 7185, 7186, 7187, 7217, 7233, 7235, 7255, 7287, 7491, 7493, 7512, 7513, 7547, 7550, 7595, 7705, 7710, 7950, 8312, 9210, 9700.

^2^

https://fda.report/media/127494/DDTBMQ000083+FNIH+N170+to+upright+human+faces+final‐OCR.pdf.
