# Supplementary material for: Misinterpreting Electrophysiology in Human Cognitive Neuroscience
Source: Psychophysiology. 2026 Apr 21;63(4):e70303. doi: 10.1111/psyp.70303 (PMC13100568; doi:10.1111/psyp.70303)
Supplement: Supplementary file 1 — Figure S1: Group‐level relationship between P1‐N170 timing and saccade onset latency. (A) Face viewing task; (B) IAPS picture viewing task. For both tasks, trials were sorted into bins according to the onset of the first saccade following stimulus onset within the 150–300 ms latency window (color‐coded from early to late saccade latencies). Left panels show stimulus‐locked ERPs averaged within each saccade‐latency bin (electrodes P7, P8, O1, O2 average reference montage), demonstrating a systematic shift in the timing of the P1‐N170 complex as a function of saccade onset latency. Right panels show the same data re‐aligned to saccade onset (saccade‐locked ERPs), revealing convergence of ERP waveforms across bins, with the negative deflection preceding saccade onset by approximately 100–150 ms. For both stimulus classes, this pattern replicates at the group level the single‐subject findings shown in Figure 5 (faces) and Figure 9 (IAPS) in the main text, albeit with reduced separation between bins due to fewer trials in the faces task than in the IAPS task and to lower trial counts for some participants. Figure S2: Stimulus‐locked and saccade‐locked ERPs reveal distinct temporal and topographic signatures of visual–oculomotor processing during passive viewing. Single participant data used in Figure 9 in the main paper. Note different y axes. (A) ERP time course locked to stimulus onset (averaged across 5496 trials), illustrating the canonical P1‐N170 complex followed by a sustained slow potential. The scalp topographies above and below the waveform depict the spatial distribution of the P1 and N170 components, respectively, highlighting their characteristic posterior‐occipital dominance. (B) ERP time course locked to first saccade onset during the 1 s viewing window (average across the 12,382 saccades that occurred in the same 5496 trials), revealing a saccadic‐spike potential (SP) tightly aligned to eye‐movement execution, followed by the P1‐N170 complex. The correspo [file PSYP-63-e70303-s001.zip › Supplemental_Material_R2_no_trackchanges.docx]

**Supplemental Information**

**Supplemental Introduction**

*Action as a Prerequisite for Visual Inference*

The mere radiation of light from an object toward the retina when both the world (e.g., bar stimulus) and the retina are static does not result in a cortical response. It is the relative motion that elicits a measurable pattern. In the seminal discovery by Hubel and Wiesel in anesthetized and paralyzed cats (hence no eye movements), single-cell firing was not recorded before “Suddenly, just as we inserted one of our glass slides into the ophthalmoscope, the cell seemed to come to life and began to fire impulses like a machine gun. It took a while to discover that the firing had nothing to do with the small opaque spot-the cell was responding to the fine moving shadow cast by the edge of the glass slide as we inserted it into the slot” ((Hubel & Wiesel, 2004), p. 61). An experimental approach utilizing “hand-held search stimuli” ((Movshon & Newsome, 1996), p. 7734) or a reversal beam ophthalmoscope that involves movements due to the nature of the reversal beam (Van Essen, Newsome, & Maunsell, 1984) uses a moving beam of light to illuminate different parts of the retina. It is the movement that allows for dynamic examination of the retina, enhancing the ability to observe details and identify abnormalities, given full anesthesia and a paralyzed participant. This strategy paved the way to record visual cells sensitive to edges, motion, and color properties of a stimulus in the anesthetized and paralyzed primate by moving and/or changing the occurrence of the stimulus over the targeted retinal position and the corresponding receptive field (e.g., (Essen & Zeki, 1978; Majaj, Carandini, & Movshon, 2007; Maunsell & Van Essen, 1987; Movshon & Newsome, 1996; Van Essen et al., 1984)). Contemporary technology might replace hand-held search movement and/or ophthalmoscope, but the requirement of relative action to infer vision remains. Why and how is this action relevant for the interpretation of data acquired in non-invasive human psychophysiology?

*Saccade Initiation and Fixation Control Operate within a Temporal Window of ~100-120 ms*

The world has to move for us to see. And when the world is (quasi) stationary, the eyes have to move for us to see. Fortunately, the eye is in constant motion (Martinez-Conde et al., 2008; Martinez-Conde, Macknik, & Hubel, 2004; Peter H. Schiller & Edward J. Tehovnik, 2001). During the awake daytime, the eye is aligned along the so-called optical axis, which is 23º nasally from the orbital axis (Figure 1 in the main paper), the latter being the principal direction of eyeball orientation in the absence of eye muscle activity. Thus, the position that would qualify as true rest is 23º away from central fixation. From empirical, first-person experience, the position of the eye during the awake state is straight ahead 0º on the optical axis, always ready to evaluate the environment. However, this position is not resting. Miniature eye muscle contractions maintain a fixation position by continuous movements around a given target (Figure 1C, main paper) (Martinez-Conde et al., 2008; Martinez-Conde et al., 2004). These fixational eye movements are instantiated and controlled by the same circuit controlling other types of ocular action such as vergence, pursuit, and saccadic eye movements. The circuit provides eye muscle control enabled by neurons in the brain stem nuclei, superior colliculus (SC), and cortex. Except for the brain stem, the coding operation of deep-layer neurons in SC and cortex foregrounds an intricate relationship. Effects of stimulation of deep pyramidal neurons by a microelectrode in SC, visual, parietal, or frontal cortex converge on the observation that independent of where the current eye position is, the same vector saccade is produced, with identical vector direction and amplitude (Figure 1) (P. H. Schiller & E. J. Tehovnik, 2001). Neither the rate nor the frequency of the microelectrode stimulation determines the initiation of the saccade. Instead, a window of approximately ~100-120 ms acts as a “reader mechanism” within the temporal boundary of which a single saccade with the same size and direction is produced (Figure 1B, main paper). Two saccades within ~200 ms, 3 within ~300 ms, and so forth (Robinson, 1972; Schiller & Stryker, 1972). This discovery has been termed a vector code or vector coding operation of the neurons and is distributed across the cortex as well as the SC (P. H. Schiller & E. J. Tehovnik, 2001). It aligns with the fact that the fastest movement in the human body is the saccade reaction time, which is experimentally confirmed to be no faster than ~100 ms (Kingstone & Klein, 1993; Schiller, Sandell, & Maunsell, 1987). The maximum bursting rate of SC, frontal eye fields (FEF), and parietal cortex neurons occurs at ~100 ms after stimulus presentation and aligns with the reaction time of the corresponding saccade (Stine, Trautmann, Jeurissen, & Shadlen, 2023; Zhu, Zhou, Constantinidis, Salinas, & Stanford, 2024). Cooling of deep-layer pyramidal neurons in the primary visual cortex (V1) eliminates saccades toward a stimulus, and temperature restoration restores the saccadic behavior identical to that before cooling (Schiller, Stryker, Cynader, & Berman, 1974). Similarly, cooling the FEF introduces a failure to maintain fixation on a target, while cooling SC is associated with an increase in saccade reaction time and reduction in saccadic amplitude (Keating & Gooley, 1988). Ablation of just two structures, SC and FEF, eliminates all visually guided, that is stimulus-induced, eye movements (Schiller & Tehovnik, 2005) and truncates the participant’s overall ocular range (Keating & Gooley, 1988). This type of eye movement control, i.e. maintaining fixation and directing eye position toward a target, is an overarching requirement built into experimental designs using fixation maintenance as a baseline in human non-invasive psychophysiology. Invasive electrophysiology in non-human primates has shown that these eye movements follow a ~100 ms temporal code. How and why is this experimental observation relevant to the interpretation of data in non-invasive human psychophysiology?

In the absence of a microelectrode “pacemaker”, an oscillation with a duty cycle of ~100 ms could, in principle, substitute for the microelectrode stimulation and the temporal window discovery mentioned above. The collective dynamic of pyramidal neurons residing in the cortical deep layers (e.g., the local field potential LFP) has been associated with oscillations in the alpha 8-14 Hz frequency range. That is, it is conceivable that an ongoing duty cycle brings enough neurons together such that their net firing aligns with a given principle direction and directs the saccade toward an object, much like the microelectrode stimulation does. Such cooperation is a requirement because the ongoing maintenance of the brain's internal dynamic interferes with single-neuron input-output integration and subsequent firing, which is artificially overcome during microelectrode stimulation.

Taken together, these observations converge on a common principle: visual inference is not a passive consequence of retinal input but depends fundamentally on action. At the physiological level, relative motion- whether externally imposed or internally generated via eye movements- is required to elicit and structure cortical responses. At the systems level, this action is implemented through tightly regulated oculomotor dynamics operating within a characteristic ~100 ms temporal window, linking neural activity, saccade initiation, and fixation control. The implications is that the signals measured in non-invasive human psychophysiology are inseparable from the underlying oculomotor processes that generate and structure them.

**Supplemental Results**

Building on this implication, the following supplementary analyses and simulations examine the temporal coupling between the P1-N170 complex and oculomotor action across stimulus-locked and saccade-locked reference frames and across multiple levels of analysis (single-subject, group-level, within-trial sequencing, and simulation), testing whether the observed electrophysiological signals can be explained by the temporal structure of oculomotor dynamics.

Supplementary Figure S1 demonstrates that the relationship between the timing of the P1-N170 complex and subsequent saccade initiation observed at the single-subject level is also present at the group level for both face viewing (Figure S1 A) and affective picture viewing (Figure S2 B). When ERPs are time-locked to stimulus onset, the latency of the P1-N170 complex varies systematically with the latency of the first saccade, such that later saccades are associated with later ERP peaks. In contrast, when the same data are aligned to saccade onset, ERP waveforms converge across latency bins, indicating a consistent temporal relationship between the P1-N170 complex and the initiation of eye movements. The attenuation of bin separation relative to single-subject analyses reflects reduced signal-to-noise ratio resulting from fewer trials per bin at the group level, rather than a qualitative difference in the underlying temporal coupling.


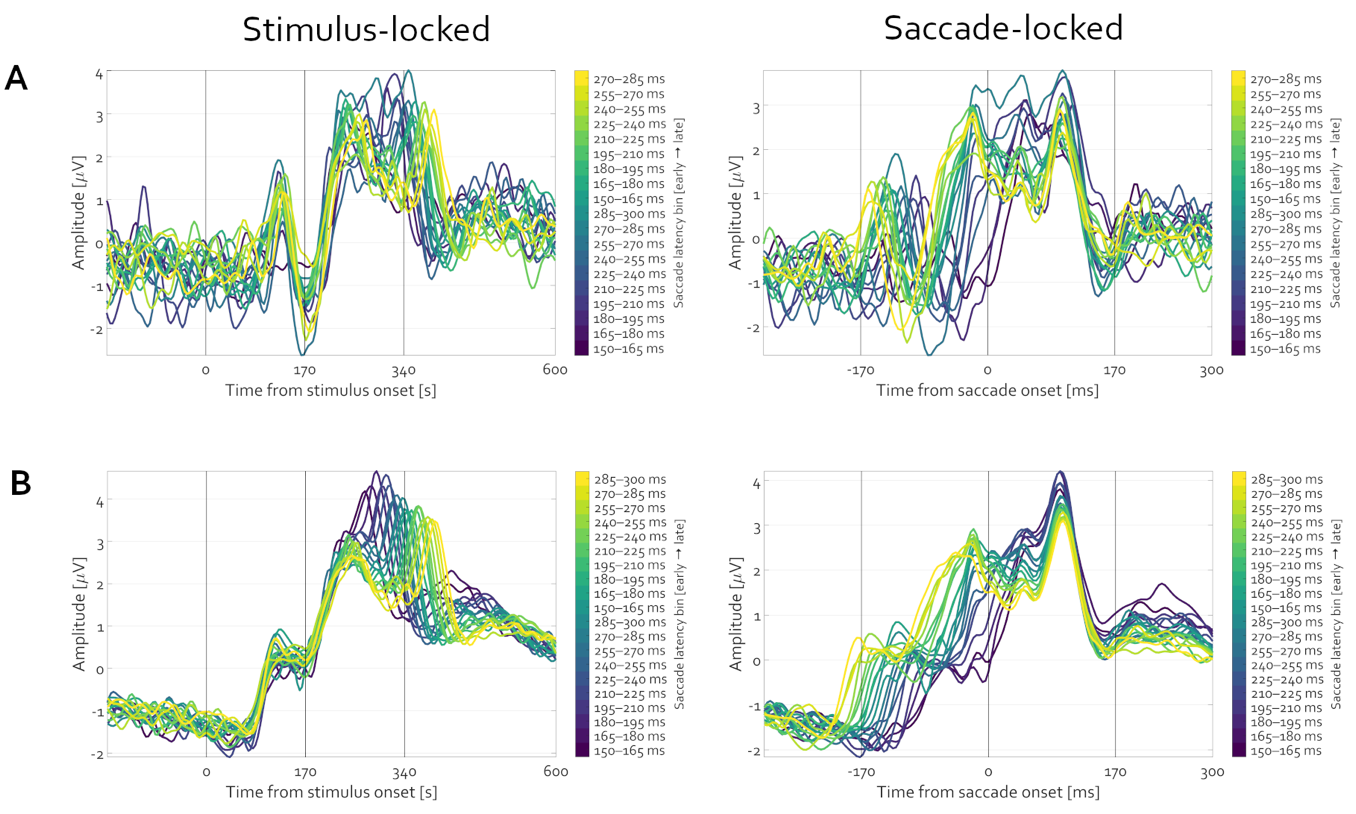


**Figure S1:** **Group-level relationship between P1-N170 timing and saccade onset latency.**

**A**- Face viewing task; **B**- IAPS picture viewing task. For both tasks, trials were sorted into bins according to the onset of the first saccade following stimulus onset within the 150-300ms latency window (color-coded from early to late saccade latencies). Left panels show stimulus-locked ERPs averaged within each saccade-latency bin (electrodes P7,P8,O1,O2 average reference montage), demonstrating a systematic shift in the timing of the P1-N170 complex as a function of saccade onset latency. Right panels show the same data re-aligned to saccade onset (saccade-locked ERPs), revealing convergence of ERP waveforms across bins, with the negative deflection preceding saccade onset by approximately 100-150 ms. For both stimulus classes, this pattern replicates at the group level the single-subject findings shown in Figure 5 (faces) and Figure 9 (IAPS) in the main text, albeit with reduced separation between bins due to fewer trials in the faces task than in the IAPS task and to lower trial counts for some participants.

### ***Relationship between P1-N170 Timing and Eye-movement Initiation during Free Viewing***

An eye movement follows rather than precedes the P1-N170 complex. If, as suggested by the results of the present work, the P1-N170 complex indexes processes related to eye-movement execution- as reflected in the increase in eye velocity following the P1-N170 waveform- it is conceivable that, in free-viewing paradigms, the P1-N170 complex would not be apparent when ERPs are aligned to saccade onset. As demonstrated in earlier work, there is a clear absence of a P1-N170 component in the pre-saccadic baseline of saccade-locked averages (e.g. (Amme et al., 2024; Dimigen & Ehinger, 2021)). Consequently, the presence of a P1-N170 component in stimulus-locked averages cannot be taken as evidence that it inevitably indexes or is needed for eye-movement execution.

To address why a clear P1-N170 response is nonetheless observed in stimulus-locked averages, but not in saccade-locked baselines, the data used for Figure 9 were analyzed with multiple temporal alignments: locked to stimulus onset and locked to successive saccades occurring within the 1-s viewing interval (single participant data as used in Figure 9 in the main paper N_trials_ = 5496). Figure S2 demonstrates that the apparent absence of a P1-N170 component in saccade-locked averages arises from temporal averaging across saccades with variable latencies. As shown in the nesting analyses of these empirical data (Figure S3) and in the accompanying simulations (Figure S4, S5), repeated P1-N170 complexes preceding successive saccades overlap in time and are averaged out when alignment does not respect their preparatory timing. Thus, the flat pre-saccadic baseline observed in earlier work is not indicative of an absence of P1–N170 activity, but rather potentially reflects the loss of temporal structure due to misalignment.


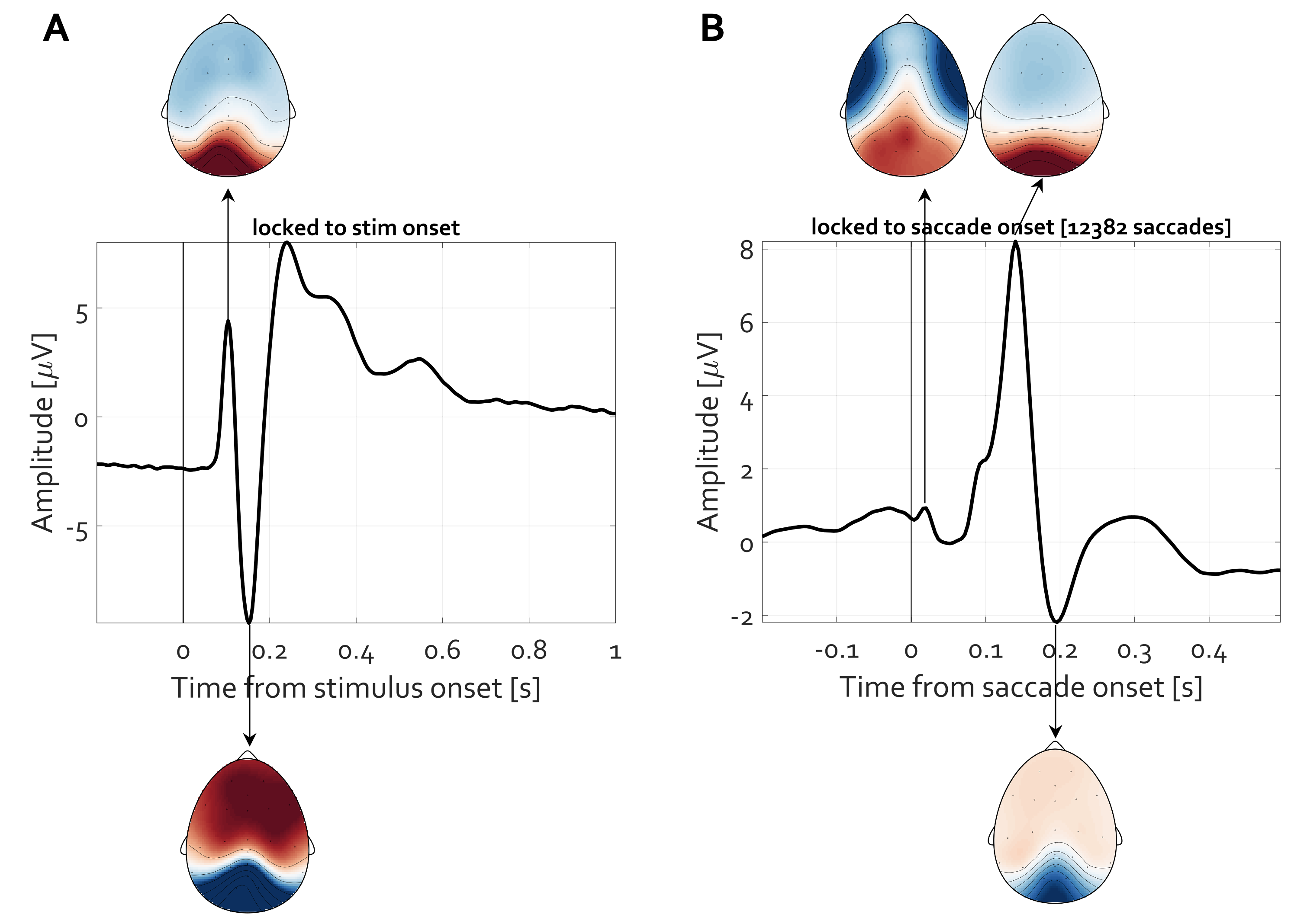


**Figure S2: Stimulus-locked and saccade-locked ERPs reveal distinct temporal and topographic signatures of visual–oculomotor processing during passive viewing.**

Single participant data used in Figure 9 in the main paper. Note different y axes. **A-** ERP time course locked to stimulus onset(averaged across 5496 trials), illustrating the canonical P1-N170 complex followed by a sustained slow potential. The scalp topographies above and below the waveform depict the spatial distribution of the P1 and N170 components, respectively, highlighting their characteristic posterior-occipital dominance. **B-** ERP time course locked to first saccade onset during the 1 s viewing window (average across the 12382 saccades that occurred in the same 5496 trials), revealing a saccadic-spike potential (SP) tightly aligned to eye-movement execution, followed by the P1-N170 complex. The corresponding scalp topographies illustrate the distribution of the SP at saccade onset, distinct from the post-saccade-onset P1 and N170 topographies, which have spatial distributions similar to those of the corresponding topographies when locked to stimulus onset.

*Sequencing of P1-N170 Complexes during Free Viewing*

In stimulus-locked analyses, participants initiate their first saccade toward the presented stimulus at different latencies following stimulus onset. Accordingly, the temporal alignment of oculomotor preparation relative to stimulus onset varies across trials. As a consequence, stimulus-locked averaging collapses across this variability, producing a seemingly well-defined P1-N170 despite substantial trial-to-trial differences in the timing of the underlying processes. Thus, the presence of a P1-N170 complex in stimulus-locked ERPs under free viewing should not be interpreted to mean that this complex is independent of eye-movement initiation. Rather, it reflects the aggregation of temporally heterogeneous visuomotor events into a common stimulus-locked reference frame.

If the timing of the P1-N170 complex is systematically coupled to oculomotor action, as proposed here the same temporal relationship should not be limited to the first saccade following stimulus onset but should generalize to subsequent saccades occurring during ongoing visual exploration. That is, each forthcoming saccade should be preceded by a P1-N170-like response the latency of which timing covaries with the latency of that saccade. In principle, this sequence structure could be examined for the third, fourth, or later saccades within a trial. In practice, however, each additional level of nesting substantially reduces the number of available trials, rendering deeper iterations statistically unreliable.

Supplementary Figure S3 directly addresses this issue by explicitly dissociating stimulus-locked and saccade-locked representations of the same data. Using the same single-participant data in Figure S2, after identifying multiple saccade onset times within the 1-s viewing window trials were sorted according to the latency of the second saccade following stimulus onset. Trials were then binned into quantiles spanning early to late second-saccade initiation within a latency range of 150-300 ms. Five bins were extracted with the following number of trials per bin: bin 1 n=131, bin 2 n=152, bin 3 n=127, bin 4 n=152, bin 5 n=147. The selection procedure and the resulting ERP waveforms are illustrated in Figure S3A.

When ERPs are aligned to stimulus onset and averaged across trials (Figure S3B, top panel), the resulting waveform closely resembles the classic P1-N170 complex. In this stimulus-locked representation, variability in the timing of saccade initiation across trials is not directly visible, because trials with early and late saccades are collapsed into a common reference frame. As a consequence, the stimulus-locked ERP appears temporally well defined, despite substantial trial-to-trial variability in the timing of subsequent eye movements. However, when ERPs are aligned to the first saccade onset (Figure S3B, middle panel), two key observations emerge. First, the classic stimulus-locked P1-N170 complex is shifted into the pre-saccadic baseline period. This shift reflects the fact that the evoked activity is no longer temporally aligned across trials, once the reference frame is moved from stimulus onset to the first saccade onset. Second, a prominent P1-N170-like deflection appears again *after* the first saccade onset. Superficially, this post-saccadic component could be interpreted as being triggered by the execution of the saccade itself or by the onset of a fixation following the saccade.

But further re-aligning the data to the onset timing of the upcoming (second) saccade reveals a nested relationship. Figure S3B (middle panel) illustrates that the post-saccadic P1-N170-like activity observed in the first-saccade-locked representation is not time-locked to the execution of the current saccade. Instead, its latency systematically covaries with the timing of the *next* eye movement. Specifically, trials associated with earlier second saccades exhibit earlier post-saccadic ERP peaks, whereas trials with later second saccades show correspondingly delayed peaks. Critically, when ERPs are aligned directly to the second saccade onset (Figure S3B, bottom panel), this apparent post-saccad response collapses back into the baseline period, and a new, tightly aligned P1-N170 complex emerges that consistently follows the second saccade by approximately 100-170 ms, evidently reflecting the oculomotor activity associated with the upcoming n^th^ saccade. Thus, the component that appears post-saccadic in the first-saccade-locked representation is in fact better understood as reflecting oculomotor preparation for the upcoming eye movement, rather than neural activity triggered by the execution of the current saccade or fixation onset.

Together, these supplemental analyses demonstrate that both stimulus-locked and saccade-locked ERP components can be misleading if interpreted without reference to the timing of forthcoming oculomotor action. The apparent emergence, disappearance, or shifting of the P1-N170 complex across reference frames reflects changes in temporal alignment, not the generation of distinct neural events.


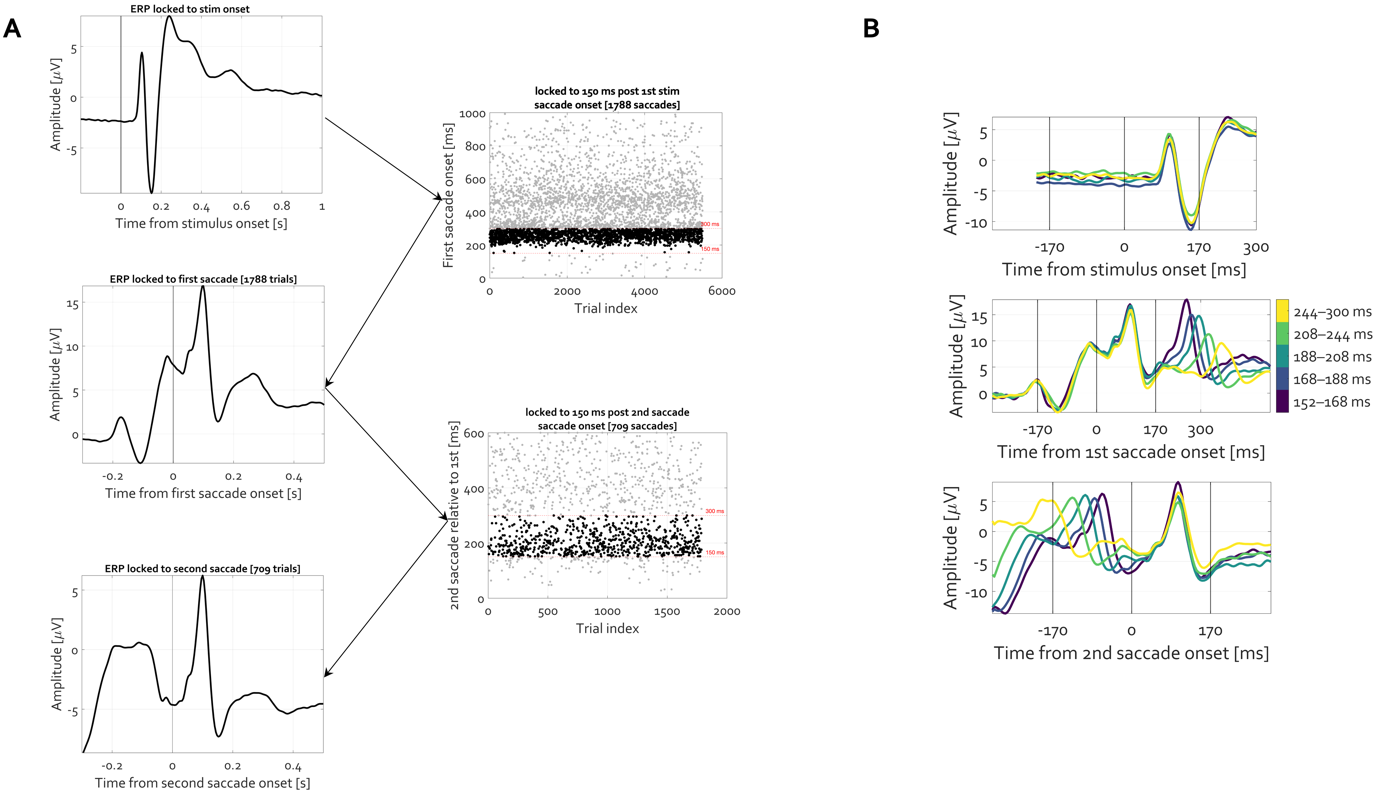


**Figure S3: Temporal sequencing of ERP components within successive saccade cycles during free viewing.**

Single participant data used in Figure 9 in the main paper N_trials_ = 5496. Note differences in y-axis scales. **A-** Left: Grand-average ERPs from the same free-viewing trials aligned to different reference events. When locked to stimulus onset (top), a classic P1-N170 complex is observed. When the same data are re-aligned to the onset of the first saccade (middle) and the second saccade (bottom), a comparable P1-N170-like response re-emerges relative to each saccade, despite identical underlying neural data. Notably, when ERPs are aligned to the onset of the first saccade, the stimulus-locked P1-N170 complex is displaced into the pre-saccadic baseline, consistent with the interpretation that stimulus-locked timing reflects overlap with oculomotor preparatory processes. Right: Distributions of first- and second-saccade latencies relative to stimulus onset. Black dots denote the saccade events included at each analysis stage, and dashed lines indicate the corresponding temporal selection windows, illustrating trial-to-trial variability and the nested timing of successive eye movements. **B-** ERPs binned by second-saccade latency with three different temporal alignments. When ERPs are aligned to stimulus onset (top), the P1-N170 complex appears largely time-locked to stimulus onset, as expected for conventional stimulus-locked averaging, and systematic latency differences across second-saccade bins are not readily apparent. When the same data are aligned to the first saccade onset (middle), a systematic latency shift of the P1-N170 complex emerges across bins defined by the timing of the *subsequent* (second) saccade. Earlier second saccades are associated with earlier ERP peaks and later second saccades with delayed peaks, replicating the temporal nesting pattern observed when binning by first-saccade latency (i.e. Figure 9C of the main manuscript). This indicates that residual variability in the ERP reflects preparation for upcoming eye movements rather than the immediately preceding saccade. When ERPs are aligned to the corresponding second saccade onset (bottom), latency differences collapse into the baseline, and post second saccade waveforms converge tightly across bins. This convergence indicates a consistent temporal relationship between the P1-N170 response and the initiation of each saccade, irrespective of absolute stimulus time.

## *Simulation of ERP components, sustained slow potentials and alpha oscillations from oculomotor action*

To clarify how classical ERP components and slower stimulus-locked potentials can arise from nested oculomotor dynamics under free viewing, a minimal generative simulation of EEG activity was conducted (Figure S4). This simulation was based on a small set of assumptions motivated by the main findings in the present work and the nested P1-N170 dynamics (e.g., Figure 5B (face exploration), Figure 9C (IAPS image free viewing) and Figure S3 above). The first assumption is that a single canonical P1-N170 waveform underlies both fixation-locked and saccade-related activity. Second, each saccade is preceded by a fixed visuomotor preparation interval, where trial-to-trial variability arises only from the timing and number of saccades. Third, no additional ERP components, cognitive processes, or slow-wave generators are introduced and/or assumed. The goal of this simulation was to test whether well-known ERP phenomena could emerge from the temporal nesting of repeated oculomotor preparation signals, without invoking additional component-specific generators.

Simulated EEG data consisted of single-channel trials, sampled at 250 Hz. Each trial spanned 3.5 s, comprising a pre-stimulus fixation baseline of .5 s, a 2 s stimulus presentation interval, and a post-stimulus return to fixation (1 s). Varying trial number, versions with 300, 1000 or 5000 trials were generated (300 matches the group and 5000 the single individual results presented in the main paper). Across all simulations, variability was introduced **only through the timing and rate of saccades (e.g., 3-4 Hz)**. The waveform associated with each visuomotor event was fixed and identical across trials and conditions. All evoked activity in the simulation was generated from a single canonical waveform kernel intended to approximate the P1-N170 complex. This kernel was defined as the sum of two Gaussian functions, peaking at 120 ms (P1) and 170 ms (N170), respectively. These latencies were chosen to match typical human ERP timing, and the kernel shape and amplitude were held constant throughout the simulation. **No late components, slow potentials, or condition-specific ERP generators were introduced**. At stimulus onset, a single instance of the P1-N170 kernel was added to every trial, reflecting the initial sensory-visuomotor response under enforced fixation. All subsequent effects emerged from temporal overlap of this same kernel. Following stimulus onset, saccades were generated stochastically within each trial using a Poisson process. Saccade timing began no earlier than 250 ms after stimulus onset and extended until stimulus offset at 2 s. To approximate natural viewing behavior, the instantaneous saccade rate decayed exponentially over time, reflecting reduced exploration and a return to fixation toward the end of the trial. Each saccade was preceded by an oculomotor preparation interval of 120 ms. Crucially, **each preparatory phase contributed the same P1-N170 kernel**, time-shifted such that its peak preceded the corresponding saccade (motivated by the findings illustrated in Figure S3). Thus, multiple identical responses could occur within a single trial, differing only in their temporal placement.

Using this simulation approach, a clear transient ERP-like P1-N170 component emerged following simulated stimulus onset (Figure S4A), followed by a sustained slow component that gradually decayed over time. This decay reflected a progressive reduction in saccade rate during image viewing, as the informational content available for exploration diminished. Consequently, as exploratory eye movements became less frequent, the amplitude of the emergent slow component also decreased, approaching baseline prior to and during the subsequent refixation period. In addition, the apparent smoothness of the emergent slow component was strongly influenced by signal-to-noise ratio (SNR), as determined by the number of trials included in the average (Figure S4 A). With more trials averaged, the slow component became progressively smoother and more clearly expressed, while its overall temporal profile remained unchanged. Importantly, this increase in smoothness arose solely from averaging and did not require any modification of the underlying generative assumptions, kernel shape, or saccade statistics. Thus, slow cortical potentials resembling classic late components can emerge naturally from the temporal overlap of repeated P1-N170 responses, with their apparent continuity and smoothness reflecting SNR (e.g., trial number within participants and/or larger sample N across participants) rather than a distinct, sustained neural process. This demonstrates that slow stimulus-locked activity can arise as a **statistical consequence of repeated, time-jittered oculomotor action**, rather than reflecting a distinct physiological component.


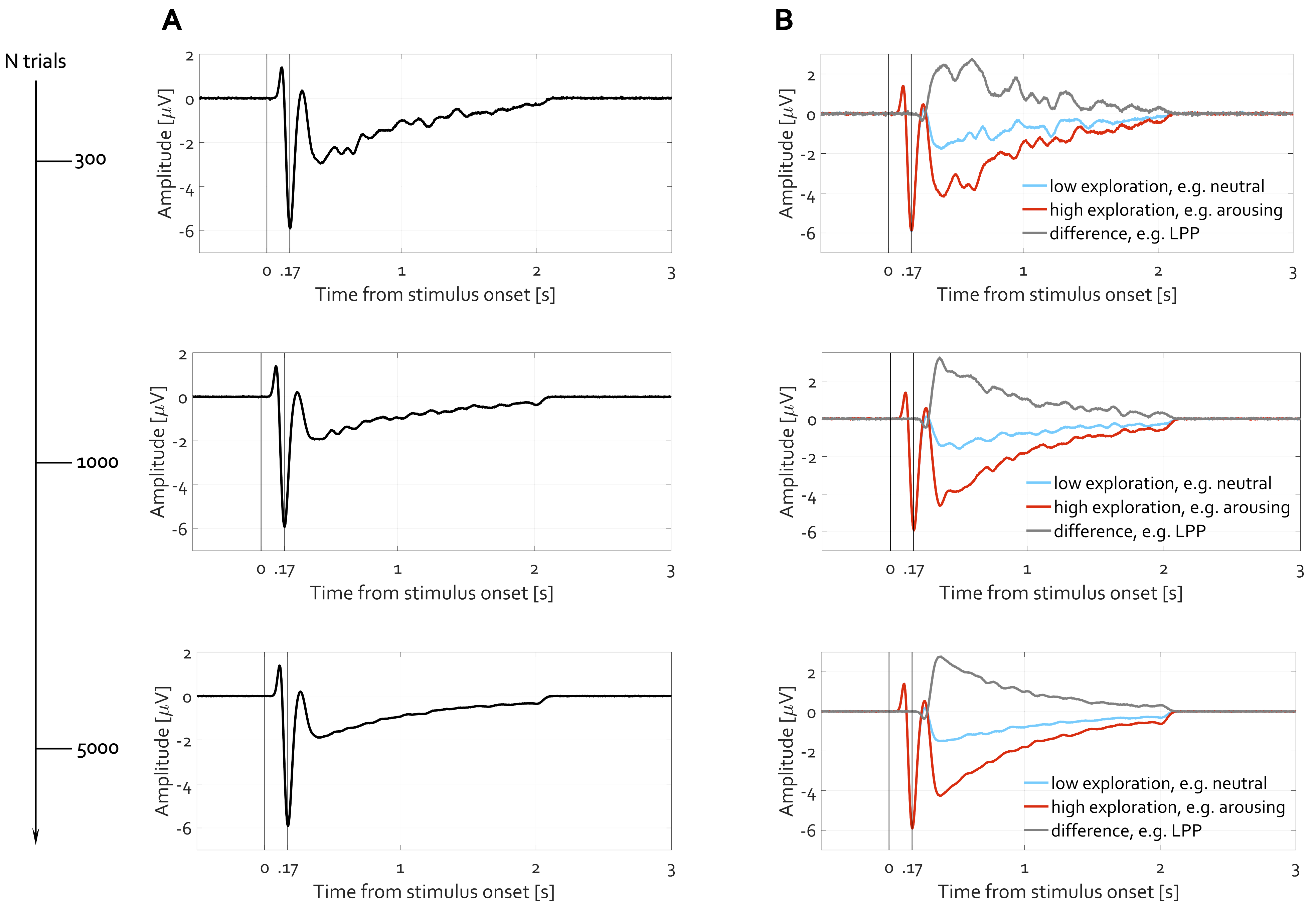


**Figure S4: Simulated emergence of ERP components and sustained slow potentials from oculomotor action.**

**A-** Stimulus-locked ERPs generated by the simulation, shown for increasing numbers of trials (top to bottom: 300, 1000, 5000). A canonical P1-N170 complex is visible following stimulus onset (vertical line at 0 s; N170 peak at ~170 ms), followed by a sustained slow component that gradually decays as saccade rate decreases over the course of stimulus viewing. As the number of trials increases, the sustained component becomes progressively smoother due to improved signal-to-noise ratio, without any change in model assumptions. **B-** Condition-specific stimulus-locked ERPs for low-exploration (e.g., neutral; blue) and high-exploration (e.g., arousing; red) trials, along with their difference waveform (gray). Differences between conditions emerge exclusively in the magnitude of sustained post-stimulus activity, reflecting increased temporal overlap of preparatory P1-N170 responses for higher saccade rates. The resulting difference waveform closely resembles a late positive potential (LPP) or a P300-like component. Vertical lines indicate stimulus onset (0 s) and N170 latency (~170 ms). Stimulus offset/return to fixation at 2 s for all illustrated time courses.

In order to model differences in visual exploration between low-exploration (e.g., “neutral“) and high-exploration (e.g. “arousing“) images, in a second simulation trials were assigned to one of two conditions differing only in their mean saccade rate. Neutral trials were simulated with a lower baseline saccade rate, whereas arousing trials exhibited a higher rate (3-fold increase relative to “neutral” trials). All other parameters-including the waveform kernel, timing, and rate decay-were identical across conditions. Stimulus-locked ERPs were then computed separately for neutral and arousing trials (Figure S4 B). Differences between conditions emerged exclusively in the magnitude of sustained post-stimulus activity, with high-exploration (e.g., “arousing”) trials exhibiting larger slow deflections due to increased temporal overlap of preparatory P1-N170 responses. The resulting difference waveform closely resembles what is traditionally identified as a late positive potential (LPP) or P300-like component.

Building on this initial simulation (Figure S4), a final test was implemented to explicitly incorporate active fixation control and its spectral consequences. In contrast to the simulations shown in Figure S4, which focused exclusively on stimulus-locked and exploration-related oculomotor events, the simulation producing Figure S5 additionally incorporated continuous fixation control both before stimulus onset and during post-stimulus refixation. Fixation was modeled as an actively maintained oculomotor state, implemented as repeated preparatory control events occurring at a characteristic timescale (~100 ms) with temporal jitter. Each fixation-related control event contributed the same canonical P1-N170 kernel used for saccade preparation, time-shifted according to its occurrence. Crucially, during fixation these control events were temporally balanced as a consequence of population vector averaging in the oculomotor system. Fixation was modeled as the continuous generation of small, directionally distributed oculomotor control signals whose net vector sum maintains gaze along the optic axis. Although each control event contributed an identical preparatory P1-N170 waveform, their temporal distribution resulted in cancellation in the time-locked ERP, while giving rise to structured alpha-band power in the time-frequency domain. No oscillatory source, rhythmic generator, or frequency-specific mechanism was introduced. Alpha-band activity emerged solely from the temporal regularity of fixation-related oculomotor control signals and was attenuated during periods of stimulus-driven exploratory viewing when this regularity was disrupted (Figure S5B). Following stimulus onset, the termination of fixation and initiation of exploratory eye movements disrupted this temporal regularity, producing a transient P1-N170 complex (Figure S5A), slow potentials, and a sustained reduction in alpha power during exploration. Importantly, following stimulus offset the re-emergence of alpha reflected the reinstatement of balanced fixation control.

In summary, the present simulation illustrates how stimulus-locked ERPs observed under free viewing can reflect the nesting and superposition of multiple oculomotor preparation events rather than purely stimulus-evoked sensory processing. It further shows that differences between experimental conditions (e.g., arousal) need not imply distinct neural components and may instead arise from systematic differences in oculomotor behavior. Canonical ERP components sustained slow potentials, and alpha-band modulation can jointly arise from the same oculomotor control mechanisms.


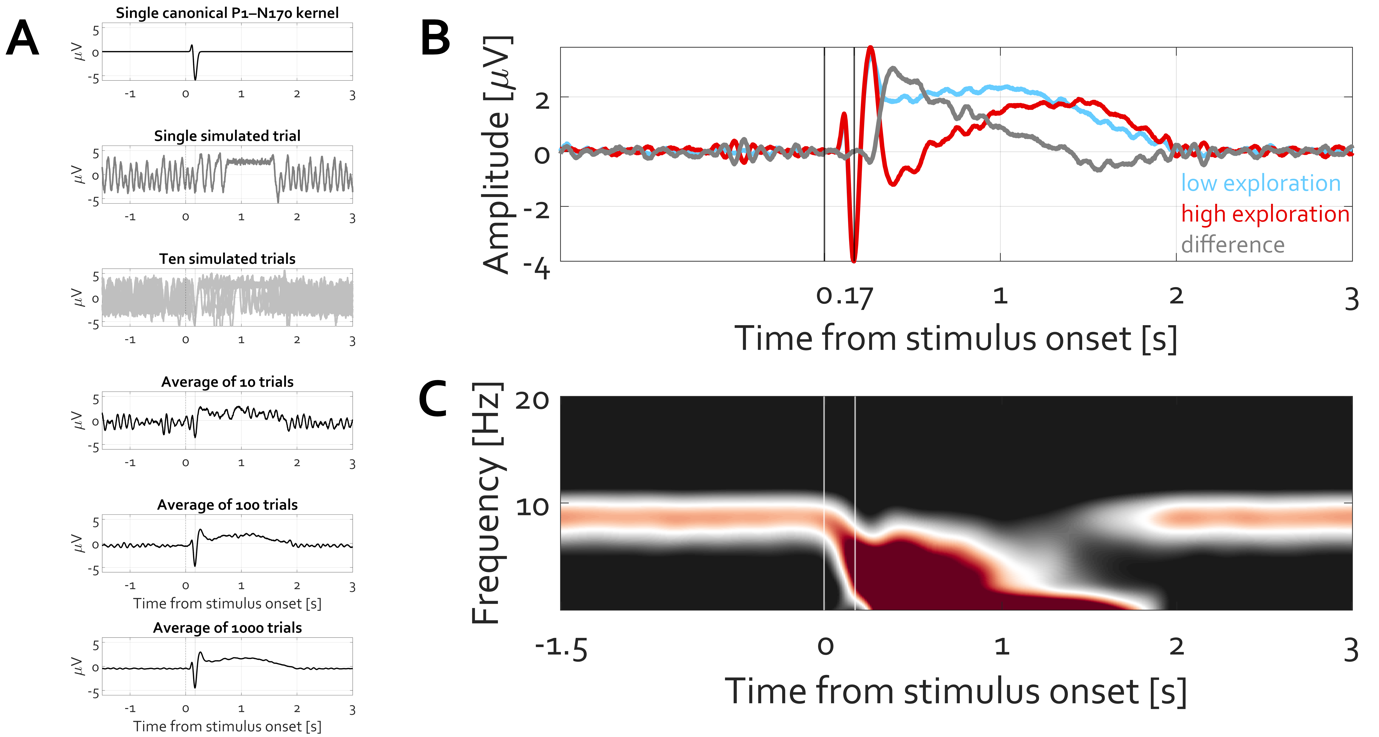


**Figure S5**: **Simulated emergence of ERP components and alpha oscillations from oculomotor action.**

**A-** Graphical illustration of the simulation procedure. **B-** Condition-specific stimulus-locked ERPs (average over 1000 trials) for low-exploration (e.g. blue) and high-exploration (e.g., red) trials, along with their difference waveform (gray). Similar to Figure S4. **C-** Time–frequency representation of simulated EEG power showing elevated activity in the alpha band (8–10 Hz) during periods of fixation. Alpha-band structure emerges from the temporal regularity of fixation-related oculomotor control signals maintaining gaze along the optic axis, and is attenuated during stimulus-driven exploratory viewing. No oscillatory source was imposed; spectral structure arises solely from the timing statistics of repeated fixation control events.

**Supplemental Discussion**

*Timing and conduction delay from Retina to Cortex*

Consideration of the timing and conduction delay starting at the level of the retina and the early entry into V1 gives some hints as to why the overall latency of the P1-N170 complex is around 170ms post stimulus onset. Recent work utilizing state-of-the-art, simultaneous recordings of retinal potentials (electroretinogram, ERG) and MEG provides the most direct clues to date in a human model system (Britta U. Westner & Dalal, 2019; B. U. Westner, Lubell, Jensen, Hokland, & Dalal, 2021). The authors measured two retinal responses, the *a-wave* and *b-wave*, which are ERG components that reflect retinal activity in response to visual stimuli. These waves reflect specific stages of retinal signal processing before the information reaches higher visual areas such as the LGN and V1. The a-wave that peaks at ~25 ms represents the initial hyperpolarization of photoreceptor cells (rods and cones) in response to light, thus very early stages of retinal signal transduction, converting light into electrical signals. The b-wave that peaks at ~80 ms reflects inner retinal activity, primarily from bipolar cells, which process and relay the photoreceptor signals to ganglion cells - a critical stage of signal integration and amplification within the retina. In fact, this is the timing of arrival of the retinal signal in the primary visual cortex reported non-invasively in humans (Britta U. Westner & Dalal, 2019). The latest time point at which V1 was informed about the events occurring on the retina was 77 ms. Converging evidence for this temporal constraint comes from fast optical imaging, which directly measures early cortical activity with millisecond resolution (Gratton & Fabiani, 2010), suggesting that responses localized to primary visual cortex peak at approximately 76 ms following stimulus onset, consistent with the arrival of retinal signals in V1. From this point on, pyramidal cells in V1 are capable of sending a signal down to SC, the brain stem, the eye muscle, and eventually direct the eye in motion toward a particular position in the visual field.

This is the chain of events observed in the non-human primate brain that is described in detail elsewhere (Schiller et al., 1974; Schiller & Tehovnik, 2003). Important here is that saccade initiation by the SC depends on the motor command from V1, i.e. a saccade initiation to terminate the experimentally defined state of fixation maintenance and to explore a face, complex image, word, etc. Thus, after ­~77 ms at the latest, this command is issued downstream along the proposed circuit of saccade generation (Tehovnik, Slocum, Carvey, & Schiller, 2005) - to SC, brainstem nuclei, and eye muscles. The transmission takes at least ~100 ms before an eye tracker records a movement of the eye in orbit [e.g.(Fischer & Ramsperger, 1984; Schiller, Haushofer, & Kendall, 2004; Stine et al., 2023)]. Summing these numbers (~77 + ~100), one derives a latency of ­~177 ms, prior to which eye movement following stimulus onset appears unlikely.

*Saccadic influences on the P1N170 complex and implications for its functional interpretation*

If N170 happens (at least begins) too early for the fovea to have moved to see stimulus features that distinguish a face (e.g., Figures 4-7), how would one account for the general agreement that N170 is enhanced for face stimuli? A recent contribution of exceptional scholarship summarizes the past and current state of the art of eye-tracking research and breaks new ground in offering a comprehensive means of jointly analyzing electrophysiological and eye-tracking data to improve the evaluation of hypotheses in psychophysiological research (Dimigen & Ehinger, 2021). A finding of particular interest for the present narrative is the observation that P1-N170 amplitude is modulated by the size and direction of saccades (e.g., Figure 7 in (Dimigen & Ehinger, 2021)). Specifically, when locked to fixation onset, the preceding saccade size and direction modulate the amplitude and retinotopic topography of the P1-N170 occurring within the first 300 ms after fixation onset. This fixation-related potential (FRP) is an electrophysiological phenomenon of its own, robust, replicable, and widely studied (Degno & Liversedge, 2020).

However, a complementary interpretation of this FRP phenomenon can be offered. Humans typically saccade 3 to 6 times per second. It follows from this that fixation duration typically varies between approximately 150 and 300 ms (Tullis & Albert, 2013). Given an average fixation duration of 264 ms (Dimigen & Ehinger, 2021), the occurrence of P1-N170, rather than being evoked by the fixation, can also signify the cortical activity associated with the motor command preceding the upcoming saccade. That is, the epoch between 0 ms (fixation onset) and 264 ms (average latency of subsequent saccade onset) includes the FRP. One can question whether this FRP is evoked by the fixation, as commonly accepted, or by the initiation of the subsequent eye movement that inevitably follows periods of fixation.

The present narrative argues for the latter (e.g., Figure 5B, Figure 9C, and Figure S1/S3 and simulations). Amplitude variation with preceding saccade size as demonstrated by (Dimigen & Ehinger, 2021) does not necessarily support the assumption that this P1-N170 amplitude variation will also apply to an upcoming saccade. However, evidence also suggests that prior saccade direction influences the direction of future saccades (Anderson, Yadav, & Carpenter, 2008; Jones, Cowper-Smith, & Westwood, 2014). Specifically, this research shows that saccadic reaction times are reduced when the direction of a current saccade matches that of a preceding saccade, a phenomenon that Jones et al. referred to as the "same direction benefit" (SDB). This benefit persists even when intervening saccades occur between two same-direction saccades, indicating that saccade direction history influences future eye movements. Hence, it is conceivable that the retinotopic and amplitude modulation of P1-N170 also informs upcoming saccade direction akin to the retinotopic modulation of the saccadic spike potential (Keren, Yuval-Greenberg, & Deouell, 2010) and alpha oscillatory activity (T. Popov, Gips, Weisz, & Jensen, 2023; Tzvetan Popov, Miller, Rockstroh, Jensen, & Langer, 2021; Quax, Dijkstra, van Staveren, Bosch, & van Gerven, 2019).

In the case of faces, the pattern of eye movements follows the prototypical triangular pattern- left eye, right eye, mouth- repeat. As indicated in the heatmaps (e.g., Figures 4, 6, 7) and known since Yarbus (Yarbus, 2013), this pattern is well-established (Dimigen & Ehinger, 2021; Spiering & Dimigen, 2024). In the case of complex scenes such as houses and IAPS images, the pattern of eye movements does not follow a consistent pattern; the saccades vary considerably in size and direction, without a clear, consistent spatial sequence across trials (e.g., Figures 9, 10). This is evident in the heatmaps for IAPS and house stimuli (Ward, 2017). That is, when viewing a house, eye movements averaged over viewers do not typically result in a pattern such as repeatedly fixating on the left roof, right roof, door, etc. It is thus conceivable that the amplitude of N170 is enhanced during face viewing due to the specific eye movements required. This proposal is supported by the established view that N170 is a robust phenomenon when controlling for stimulus complexity (e.g., (Johnston, Molyneux, & Young, 2014) ). Yet, this stimulus complexity involves large-amplitude saccades picturing the silhouette of a triangle when viewing faces vs. small-amplitude saccades and fixational eye movements when evaluating scenes.

Recent work concludes that it is the saccade onset and not fixation onset that is the behavioral event that triggers and best explains the manifestation of early visual evoked responses such as P1-N170 discussed here (Amme et al., 2024). This proposal contradicts the FRP literature and challenges the notion that a fixation event by itself can elicit P1-N170. Instead, Amme and colleagues noted that saccade duration and therefore amplitude (as described by the saccade “main sequence” (Bahill, Clark, & Stark, 1975)) predict the peak latency of the P1 component. When locked to fixation onset, the authors observed that, the shorter the saccade, the longer the P1 peak latency, and vice versa. In addition, small saccades were associated with smaller P1 amplitude and vice versa. Thus, the amplitude and duration of the oculomotor event are closely linked to the amplitude and latency of P1-N170.

Accepting the premise that the default state of the eye is movement, and rejecting the assumption of fixation as a stationary event independent of continuous, active oculomotor control, these seemingly contradictory findings can be reconciled by considering the possibility that the strategy of time-locking the data on fixation or saccade onset provides a view of the same oculomotor event from two different vantage points, thus both valid observations representing the variability in the electrophysiological data at the rise of P1-N170 when locked to fixation onset and at the decline when locked to saccade onset. In fact, a closer look at both Dimingen and Ehinger arguing for FRP (e.g., Figure 7F in (Dimigen & Ehinger, 2021)) and Amme and colleagues arguing against it (e.g., Figure 1D in (Amme et al., 2024)) reveals fair agreement, in that in both reports - saccade size prior to fixation onset (Dimigen & Ehinger, 2021) or saccade size following the fixation onset (Amme et al., 2024) - the same phenomenon is observed: the smaller the saccade amplitude, the later the P1-N170 peak latencies and the smaller their peak amplitudes. The converse also appears true.

The covariance of oculomotor action with cortical activation in psychophysiology has been noted repeatedly [e.g., (Carl, Açık, König, Engel, & Hipp, 2012; Dimigen, Valsecchi, Sommer, & Kliegl, 2009; Liu, Nobre, & van Ede, 2023; Meyberg, Werkle-Bergner, Sommer, & Dimigen, 2015; Yuval-Greenberg, Tomer, Keren, Nelken, & Deouell, 2008)] including in one of the first reports on visual evoked responses (Figure 2 in (Chapman & Bragdon, 1964)). Recording ERG, EOG, and EEG simultaneously, the 1964 paper concluded that the P1-N170 complex (not referred to as such at that time) was due to the cognitive operation under study rather than to oculomotor action. The latter was found to occur *after*, and not simultaneously with, the ERP complex and was therefore deemed irrelevant to the interpretation of the measured ERP. This conclusion has been widely accepted in psychophysiological research the past 60 years, manifesting in the predominant premise that ERP components reflect the cognitive operations under study. However, oculomotor action is not merely an artifact that can be traditionally addressed through data preprocessing and statistical removal. Instead, it is itself “a genuine cortical activity and not an EEG-specific artifact … also frequently overlaid on magnetoencephalographic and possibly hemodynamic datasets” ((Dimigen et al., 2009), p. 12330). Therefore, misinterpretations and conclusions derived from EEG and MEG data encourage neglect of complementary alternatives and result in misjudgments with potentially serious consequences.

**Supplemental** **References**

Amme, C., Sulewski, P., Spaak, E., Hebart, M. N., König, P., & Kietzmann, T. C. (2024). Saccade onset, not fixation onset, best explains early responses across the human visual cortex during naturalistic vision. *bioRxiv*, 2024.2010.2025.620167. doi:10.1101/2024.10.25.620167

Anderson, A. J., Yadav, H., & Carpenter, R. H. (2008). Directional prediction by the saccadic system. *Curr Biol, 18*(8), 614-618. doi:10.1016/j.cub.2008.03.057

Bahill, A. T., Clark, M. R., & Stark, L. (1975). The main sequence, a tool for studying human eye movements. *Mathematical Biosciences, 24*(3), 191-204. doi:<https://doi.org/10.1016/0025-5564(75)90075-9>

Carl, C., Açık, A., König, P., Engel, A. K., & Hipp, J. F. (2012). The saccadic spike artifact in MEG. *Neuroimage, 59*(2), 1657-1667. doi:<https://doi.org/10.1016/j.neuroimage.2011.09.020>

Chapman, R. M., & Bragdon, H. R. (1964). Evoked Responses to Numerical and Non-Numerical Visual Stimuli while Problem Solving. *Nature, 203*(4950), 1155-1157. doi:10.1038/2031155a0

Degno, F., & Liversedge, S. P. (2020). Eye Movements and Fixation-Related Potentials in Reading: A Review. *Vision (Basel), 4*(1). doi:10.3390/vision4010011

Dimigen, O., & Ehinger, B. V. (2021). Regression-based analysis of combined EEG and eye-tracking data: Theory and applications. *J Vis, 21*(1), 3. doi:10.1167/jov.21.1.3

Dimigen, O., Valsecchi, M., Sommer, W., & Kliegl, R. (2009). Human microsaccade-related visual brain responses. *J Neurosci, 29*(39), 12321-12331. doi:10.1523/jneurosci.0911-09.2009

Essen, D. C., & Zeki, S. M. (1978). The topographic organization of rhesus monkey prestriate cortex. *J Physiol, 277*, 193-226. doi:10.1113/jphysiol.1978.sp012269

Fischer, B., & Ramsperger, E. (1984). Human express saccades: extremely short reaction times of goal directed eye movements. *Experimental Brain Research, 57*(1), 191-195. doi:10.1007/BF00231145

Gratton, G., & Fabiani, M. (2010). Fast optical imaging of human brain function. *Frontiers in Human Neuroscience, Volume 4 - 2010*. doi:10.3389/fnhum.2010.00052

Hubel, D. H., & Wiesel, T. N. (2004). *Brain and Visual Perception: The Story of a 25-year Collaboration*: Oxford University Press.

Johnston, P., Molyneux, R., & Young, A. W. (2014). The N170 observed ‘in the wild’: robust event-related potentials to faces in cluttered dynamic visual scenes. *Social Cognitive and Affective Neuroscience, 10*(7), 938-944. doi:10.1093/scan/nsu136

Jones, S. A., Cowper-Smith, C. D., & Westwood, D. A. (2014). Directional interactions between current and prior saccades. *Front Hum Neurosci, 8*, 872. doi:10.3389/fnhum.2014.00872

Keating, E. G., & Gooley, S. G. (1988). Saccadic disorders caused by cooling the superior colliculus or the frontal eye field, or from combined lesions of both structures. *Brain Res, 438*(1-2), 247-255. doi:10.1016/0006-8993(88)91343-1

Keren, A. S., Yuval-Greenberg, S., & Deouell, L. Y. (2010). Saccadic spike potentials in gamma-band EEG: characterization, detection and suppression. *Neuroimage, 49*(3), 2248-2263. doi:10.1016/j.neuroimage.2009.10.057

Kingstone, A., & Klein, R. M. (1993). What are human express saccades? *Percept Psychophys, 54*(2), 260-273. doi:10.3758/bf03211762

Liu, B., Nobre, A. C., & van Ede, F. (2023). Microsaccades transiently lateralise EEG alpha activity. *Prog Neurobiol, 224*, 102433. doi:10.1016/j.pneurobio.2023.102433

Majaj, N. J., Carandini, M., & Movshon, J. A. (2007). Motion integration by neurons in macaque MT is local, not global. *J Neurosci, 27*(2), 366-370. doi:10.1523/JNEUROSCI.3183-06.2007

Martinez-Conde, S., Krauzlis, R., Miller, J., Morrone, C., Williams, D., & Kowler, E. (2008). Eye movements and the perception of a clear and stable visual world. *J Vis, 8*(14), 1. doi:10.1167/8.14.i

Martinez-Conde, S., Macknik, S. L., & Hubel, D. H. (2004). The role of fixational eye movements in visual perception. *Nat Rev Neurosci, 5*(3), 229-240. doi:10.1038/nrn1348

Maunsell, J. H., & Van Essen, D. C. (1987). Topographic organization of the middle temporal visual area in the macaque monkey: representational biases and the relationship to callosal connections and myeloarchitectonic boundaries. *J Comp Neurol, 266*(4), 535-555. doi:10.1002/cne.902660407

Meyberg, S., Werkle-Bergner, M., Sommer, W., & Dimigen, O. (2015). Microsaccade-related brain potentials signal the focus of visuospatial attention. *Neuroimage, 104*, 79-88. doi:<https://doi.org/10.1016/j.neuroimage.2014.09.065>

Movshon, J. A., & Newsome, W. T. (1996). Visual response properties of striate cortical neurons projecting to area MT in macaque monkeys. *J Neurosci, 16*(23), 7733-7741. doi:10.1523/JNEUROSCI.16-23-07733.1996

Popov, T., Gips, B., Weisz, N., & Jensen, O. (2023). Brain areas associated with visual spatial attention display topographic organization during auditory spatial attention. *Cereb Cortex, 33*(7), 3478-3489. doi:10.1093/cercor/bhac285

Popov, T., Miller, G. A., Rockstroh, B., Jensen, O., & Langer, N. (2021). Alpha oscillations link action to cognition: An oculomotor account of the brain’s dominant rhythm. *bioRxiv*, 2021.2009.2024.461634. doi:10.1101/2021.09.24.461634

Quax, S. C., Dijkstra, N., van Staveren, M. J., Bosch, S. E., & van Gerven, M. A. J. (2019). Eye movements explain decodability during perception and cued attention in MEG. *Neuroimage, 195*, 444-453. doi:10.1016/j.neuroimage.2019.03.069

Robinson, D. A. (1972). Eye movements evoked by collicular stimulation in the alert monkey. *Vision Research, 12*(11), 1795-1808. doi:<https://doi.org/10.1016/0042-6989(72)90070-3>

Schiller, P. H., Haushofer, J., & Kendall, G. (2004). An examination of the variables that affect express saccade generation. *Vis Neurosci, 21*(2), 119-127. doi:10.1017/s0952523804042038

Schiller, P. H., Sandell, J. H., & Maunsell, J. H. (1987). The effect of frontal eye field and superior colliculus lesions on saccadic latencies in the rhesus monkey. *J Neurophysiol, 57*(4), 1033-1049. doi:10.1152/jn.1987.57.4.1033

Schiller, P. H., & Stryker, M. (1972). Single-unit recording and stimulation in superior colliculus of the alert rhesus monkey. *J Neurophysiol, 35*(6), 915-924. doi:10.1152/jn.1972.35.6.915

Schiller, P. H., Stryker, M., Cynader, M., & Berman, N. (1974). Response characteristics of single cells in the monkey superior colliculus following ablation or cooling of visual cortex. *J Neurophysiol, 37*(1), 181-194. doi:10.1152/jn.1974.37.1.181

Schiller, P. H., & Tehovnik, E. J. (2001). Chapter 9 Look and see: how the brain moves your eyes about. In *Progress in Brain Research* (Vol. 134, pp. 127-142): Elsevier.

Schiller, P. H., & Tehovnik, E. J. (2001). Look and see: how the brain moves your eyes about. *Prog Brain Res, 134*, 127-142. doi:10.1016/s0079-6123(01)34010-4

Schiller, P. H., & Tehovnik, E. J. (2003). Cortical inhibitory circuits in eye-movement generation. *Eur J Neurosci, 18*(11), 3127-3133. doi:10.1111/j.1460-9568.2003.03036.x

Schiller, P. H., & Tehovnik, E. J. (2005). Neural mechanisms underlying target selection with saccadic eye movements. *Prog Brain Res, 149*, 157-171. doi:10.1016/s0079-6123(05)49012-3

Spiering, L., & Dimigen, O. (2024). (Micro)saccade-related potentials during face recognition: A study combining EEG, eye-tracking, and deconvolution modeling. *Attention, Perception, & Psychophysics*. doi:10.3758/s13414-024-02846-1

Stine, G. M., Trautmann, E. M., Jeurissen, D., & Shadlen, M. N. (2023). A neural mechanism for terminating decisions. *Neuron, 111*(16), 2601-2613.e2605. doi:<https://doi.org/10.1016/j.neuron.2023.05.028>

Tehovnik, E. J., Slocum, W. M., Carvey, C. E., & Schiller, P. H. (2005). Phosphene induction and the generation of saccadic eye movements by striate cortex. *J Neurophysiol, 93*(1), 1-19. doi:10.1152/jn.00736.2004

Tullis, T., & Albert, B. (2013). Chapter 7 - Behavioral and Physiological Metrics. In T. Tullis & B. Albert (Eds.), *Measuring the User Experience (Second Edition)* (pp. 163-186). Boston: Morgan Kaufmann.

Van Essen, D. C., Newsome, W. T., & Maunsell, J. H. (1984). The visual field representation in striate cortex of the macaque monkey: asymmetries, anisotropies, and individual variability. *Vision Res, 24*(5), 429-448. doi:10.1016/0042-6989(84)90041-5

Ward, A. S. J. M. (2017, 06 Dec 2017). Here's What You Can Learn About Architecture from Tracking People's Eye Movements. *ArchDaily*. Retrieved from <https://www.archdaily.com/884945/heres-what-you-can-learn-about-architecture-from-tracking-peoples-eye-movements>

Westner, B. U., & Dalal, S. S. (2019). Faster than the brain’s speed of light: Retinocortical interactions differ in high frequency activity when processing darks and lights. *bioRxiv*, 153551. doi:10.1101/153551

Westner, B. U., Lubell, J. I., Jensen, M., Hokland, S., & Dalal, S. S. (2021). Contactless measurements of retinal activity using optically pumped magnetometers. *Neuroimage, 243*, 118528. doi:10.1016/j.neuroimage.2021.118528

Yarbus, A. L. (2013). *Eye movements and vision*: Springer.

Yuval-Greenberg, S., Tomer, O., Keren, A. S., Nelken, I., & Deouell, L. Y. (2008). Transient induced gamma-band response in EEG as a manifestation of miniature saccades. *Neuron, 58*(3), 429-441. doi:10.1016/j.neuron.2008.03.027

Zhu, J., Zhou, X. M., Constantinidis, C., Salinas, E., & Stanford, T. R. (2024). Parallel signatures of cognitive maturation in primate antisaccade performance and prefrontal activity. *iScience, 27*(8), 110488. doi:<https://doi.org/10.1016/j.isci.2024.110488>
