# Supplementary figures and images for: Misinterpreting Electrophysiology in Human Cognitive Neuroscience
Source: Psychophysiology. 2026 Apr 21;63(4):e70303. doi: 10.1111/psyp.70303 (PMC13100568; doi:10.1111/psyp.70303)

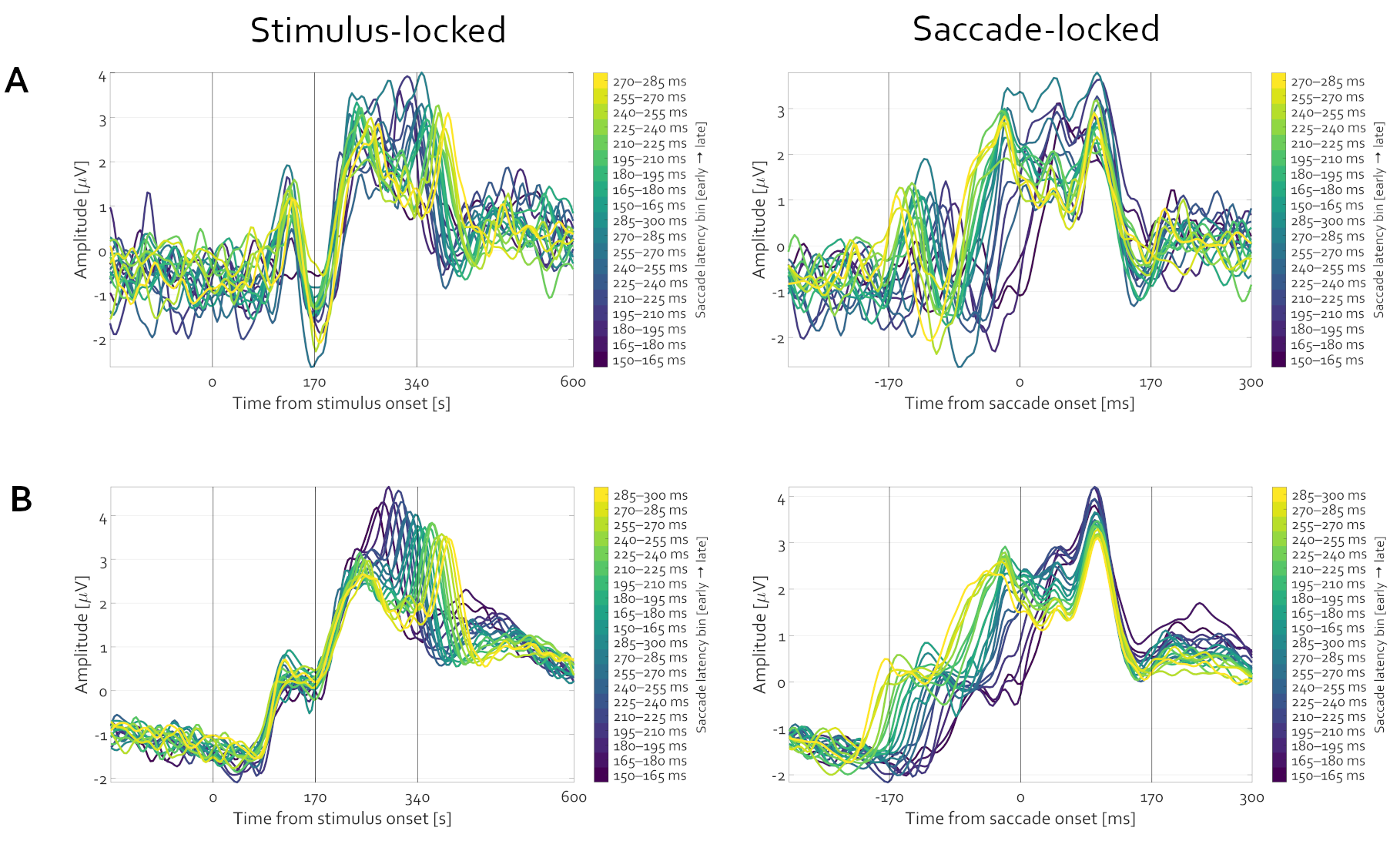

Supplement: Supplementary file 1 — Figure S1: Group‐level relationship between P1‐N170 timing and saccade onset latency. (A) Face viewing task; (B) IAPS picture viewing task. For both tasks, trials were sorted into bins according to the onset of the first saccade following stimulus onset within the 150–300 ms latency window (color‐coded from early to late saccade latencies). Left panels show stimulus‐locked ERPs averaged within each saccade‐latency bin (electrodes P7, P8, O1, O2 average reference montage), demonstrating a systematic shift in the timing of the P1‐N170 complex as a function of saccade onset latency. Right panels show the same data re‐aligned to saccade onset (saccade‐locked ERPs), revealing convergence of ERP waveforms across bins, with the negative deflection preceding saccade onset by approximately 100–150 ms. For both stimulus classes, this pattern replicates at the group level the single‐subject findings shown in Figure 5 (faces) and Figure 9 (IAPS) in the main text, albeit with reduced separation between bins due to fewer trials in the faces task than in the IAPS task and to lower trial counts for some participants. Figure S2: Stimulus‐locked and saccade‐locked ERPs reveal distinct temporal and topographic signatures of visual–oculomotor processing during passive viewing. Single participant data used in Figure 9 in the main paper. Note different y axes. (A) ERP time course locked to stimulus onset (averaged across 5496 trials), illustrating the canonical P1‐N170 complex followed by a sustained slow potential. The scalp topographies above and below the waveform depict the spatial distribution of the P1 and N170 components, respectively, highlighting their characteristic posterior‐occipital dominance. (B) ERP time course locked to first saccade onset during the 1 s viewing window (average across the 12,382 saccades that occurred in the same 5496 trials), revealing a saccadic‐spike potential (SP) tightly aligned to eye‐movement execution, followed by the P1‐N170 complex. The correspo [file PSYP-63-e70303-s001.zip › Figure S1.png]

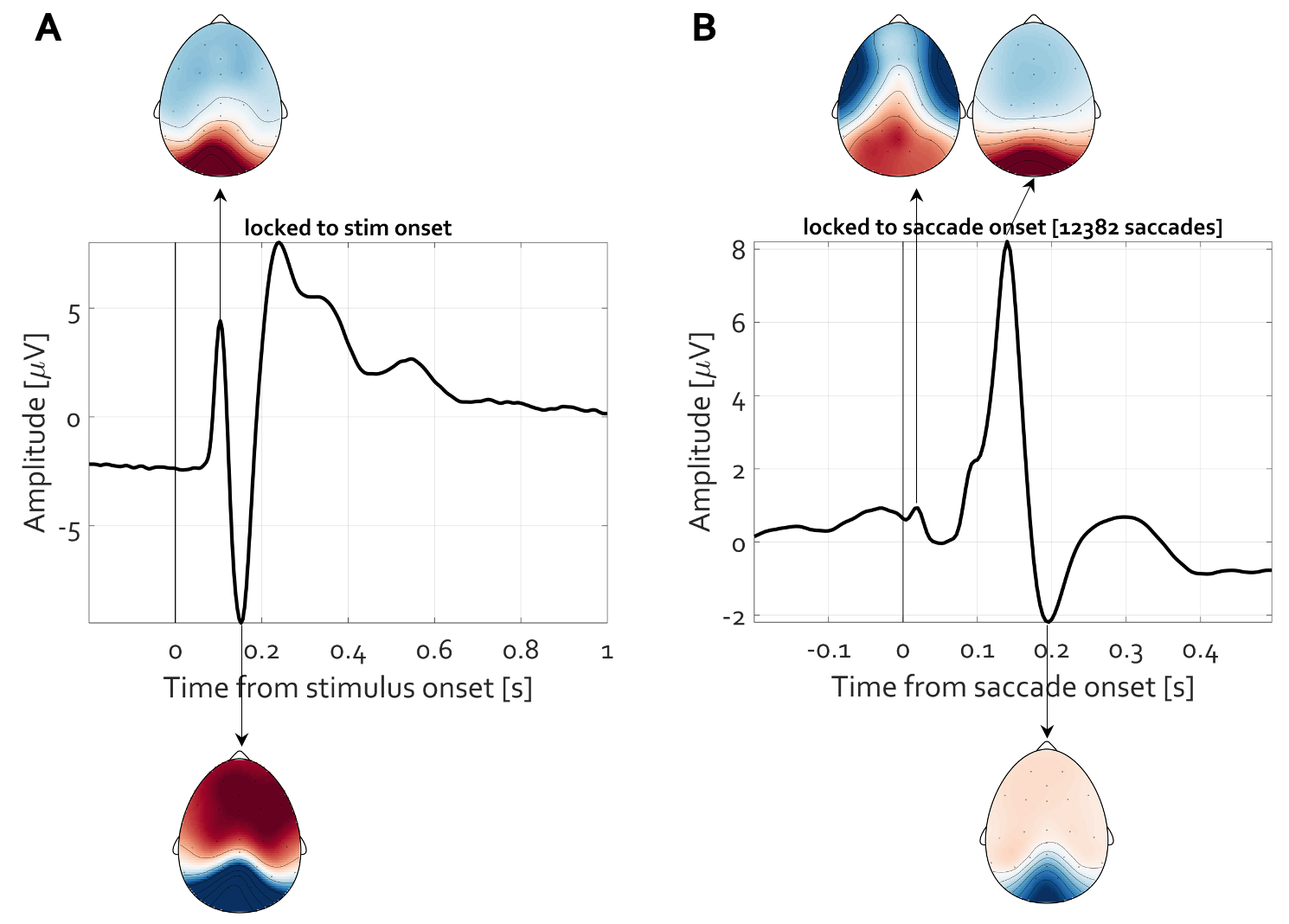

Supplement: Supplementary file 1 — Figure S1: Group‐level relationship between P1‐N170 timing and saccade onset latency. (A) Face viewing task; (B) IAPS picture viewing task. For both tasks, trials were sorted into bins according to the onset of the first saccade following stimulus onset within the 150–300 ms latency window (color‐coded from early to late saccade latencies). Left panels show stimulus‐locked ERPs averaged within each saccade‐latency bin (electrodes P7, P8, O1, O2 average reference montage), demonstrating a systematic shift in the timing of the P1‐N170 complex as a function of saccade onset latency. Right panels show the same data re‐aligned to saccade onset (saccade‐locked ERPs), revealing convergence of ERP waveforms across bins, with the negative deflection preceding saccade onset by approximately 100–150 ms. For both stimulus classes, this pattern replicates at the group level the single‐subject findings shown in Figure 5 (faces) and Figure 9 (IAPS) in the main text, albeit with reduced separation between bins due to fewer trials in the faces task than in the IAPS task and to lower trial counts for some participants. Figure S2: Stimulus‐locked and saccade‐locked ERPs reveal distinct temporal and topographic signatures of visual–oculomotor processing during passive viewing. Single participant data used in Figure 9 in the main paper. Note different y axes. (A) ERP time course locked to stimulus onset (averaged across 5496 trials), illustrating the canonical P1‐N170 complex followed by a sustained slow potential. The scalp topographies above and below the waveform depict the spatial distribution of the P1 and N170 components, respectively, highlighting their characteristic posterior‐occipital dominance. (B) ERP time course locked to first saccade onset during the 1 s viewing window (average across the 12,382 saccades that occurred in the same 5496 trials), revealing a saccadic‐spike potential (SP) tightly aligned to eye‐movement execution, followed by the P1‐N170 complex. The correspo [file PSYP-63-e70303-s001.zip › Figure S2.png]

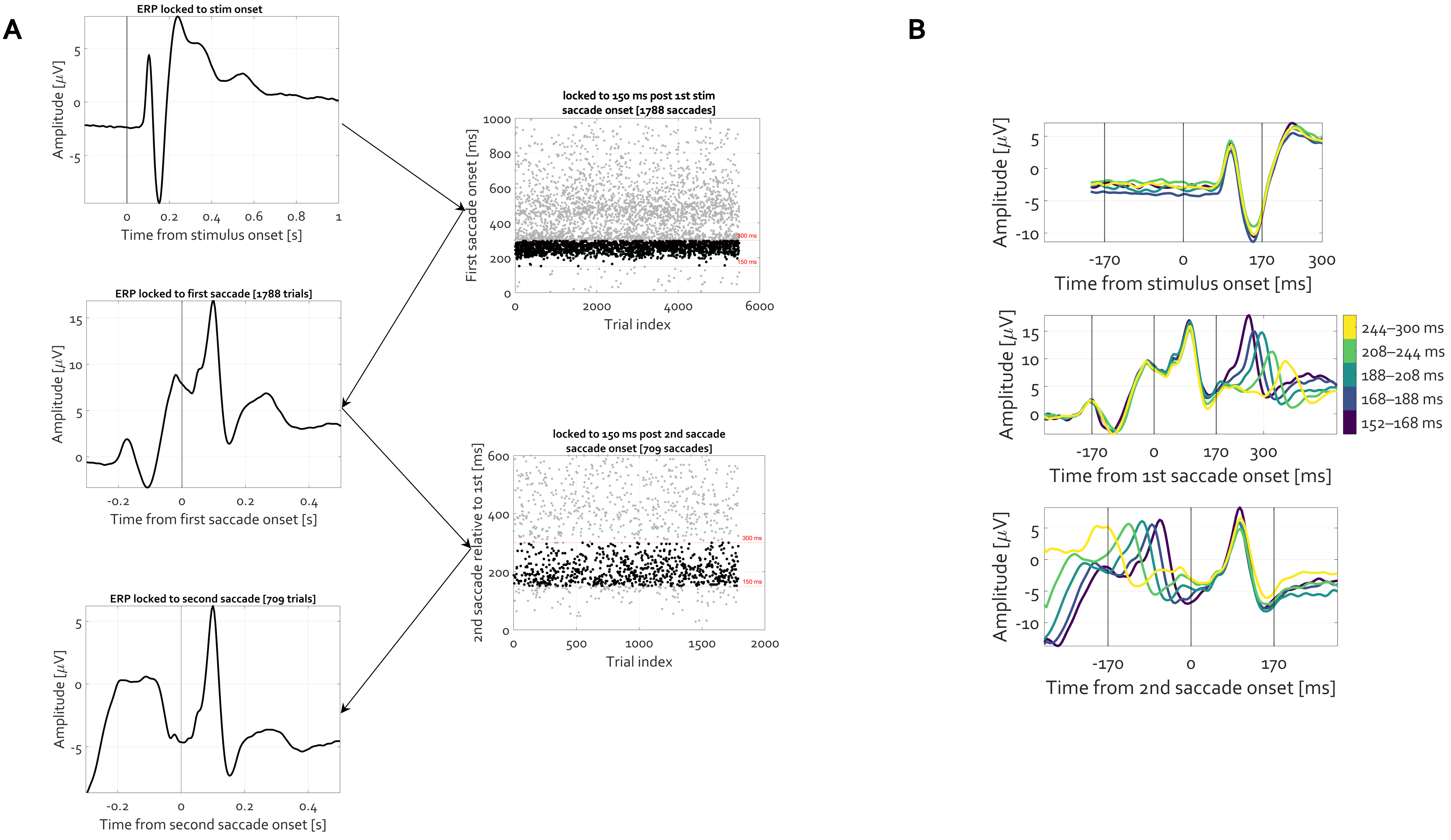

Supplement: Supplementary file 1 — Figure S1: Group‐level relationship between P1‐N170 timing and saccade onset latency. (A) Face viewing task; (B) IAPS picture viewing task. For both tasks, trials were sorted into bins according to the onset of the first saccade following stimulus onset within the 150–300 ms latency window (color‐coded from early to late saccade latencies). Left panels show stimulus‐locked ERPs averaged within each saccade‐latency bin (electrodes P7, P8, O1, O2 average reference montage), demonstrating a systematic shift in the timing of the P1‐N170 complex as a function of saccade onset latency. Right panels show the same data re‐aligned to saccade onset (saccade‐locked ERPs), revealing convergence of ERP waveforms across bins, with the negative deflection preceding saccade onset by approximately 100–150 ms. For both stimulus classes, this pattern replicates at the group level the single‐subject findings shown in Figure 5 (faces) and Figure 9 (IAPS) in the main text, albeit with reduced separation between bins due to fewer trials in the faces task than in the IAPS task and to lower trial counts for some participants. Figure S2: Stimulus‐locked and saccade‐locked ERPs reveal distinct temporal and topographic signatures of visual–oculomotor processing during passive viewing. Single participant data used in Figure 9 in the main paper. Note different y axes. (A) ERP time course locked to stimulus onset (averaged across 5496 trials), illustrating the canonical P1‐N170 complex followed by a sustained slow potential. The scalp topographies above and below the waveform depict the spatial distribution of the P1 and N170 components, respectively, highlighting their characteristic posterior‐occipital dominance. (B) ERP time course locked to first saccade onset during the 1 s viewing window (average across the 12,382 saccades that occurred in the same 5496 trials), revealing a saccadic‐spike potential (SP) tightly aligned to eye‐movement execution, followed by the P1‐N170 complex. The correspo [file PSYP-63-e70303-s001.zip › Figure S3.png]

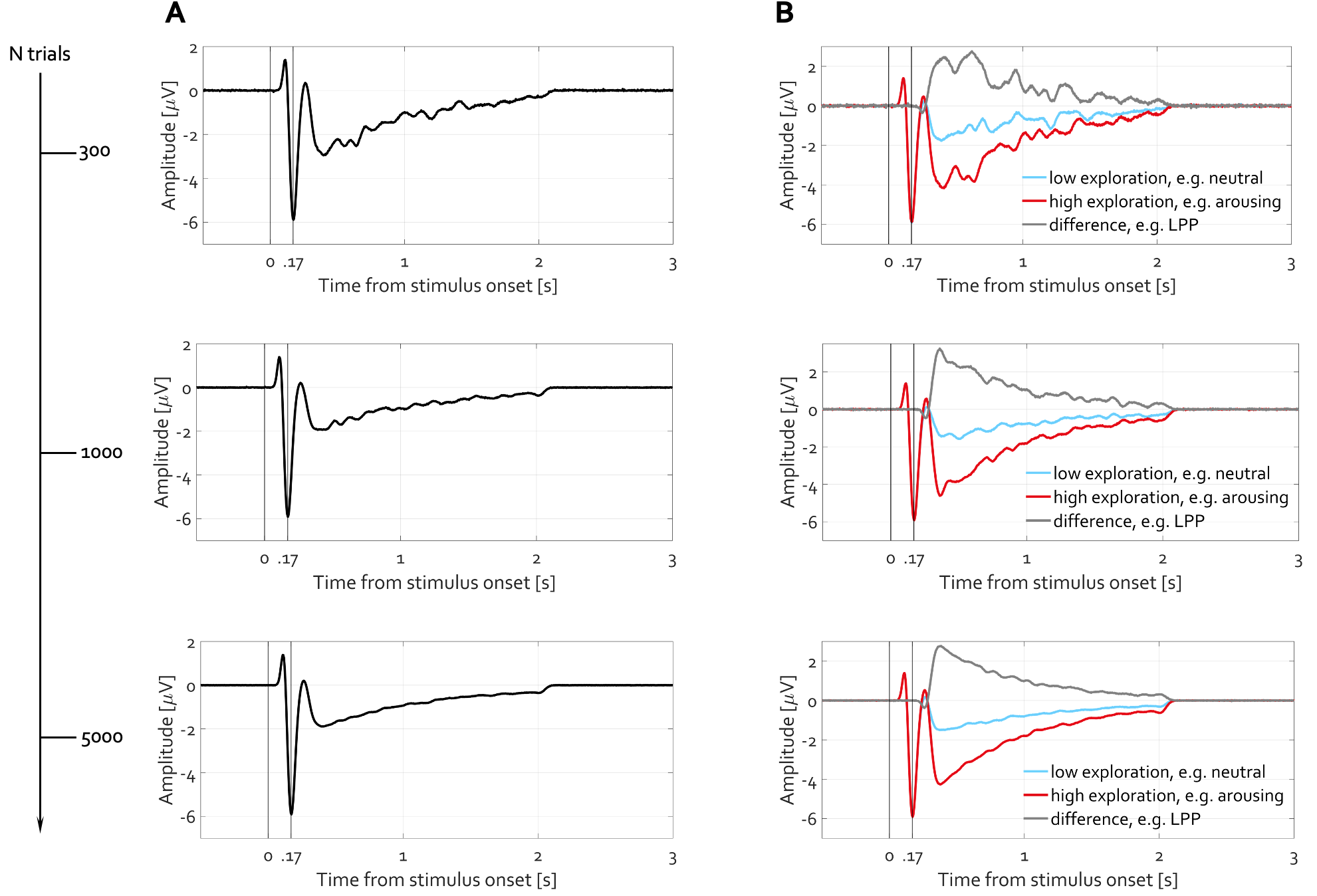

Supplement: Supplementary file 1 — Figure S1: Group‐level relationship between P1‐N170 timing and saccade onset latency. (A) Face viewing task; (B) IAPS picture viewing task. For both tasks, trials were sorted into bins according to the onset of the first saccade following stimulus onset within the 150–300 ms latency window (color‐coded from early to late saccade latencies). Left panels show stimulus‐locked ERPs averaged within each saccade‐latency bin (electrodes P7, P8, O1, O2 average reference montage), demonstrating a systematic shift in the timing of the P1‐N170 complex as a function of saccade onset latency. Right panels show the same data re‐aligned to saccade onset (saccade‐locked ERPs), revealing convergence of ERP waveforms across bins, with the negative deflection preceding saccade onset by approximately 100–150 ms. For both stimulus classes, this pattern replicates at the group level the single‐subject findings shown in Figure 5 (faces) and Figure 9 (IAPS) in the main text, albeit with reduced separation between bins due to fewer trials in the faces task than in the IAPS task and to lower trial counts for some participants. Figure S2: Stimulus‐locked and saccade‐locked ERPs reveal distinct temporal and topographic signatures of visual–oculomotor processing during passive viewing. Single participant data used in Figure 9 in the main paper. Note different y axes. (A) ERP time course locked to stimulus onset (averaged across 5496 trials), illustrating the canonical P1‐N170 complex followed by a sustained slow potential. The scalp topographies above and below the waveform depict the spatial distribution of the P1 and N170 components, respectively, highlighting their characteristic posterior‐occipital dominance. (B) ERP time course locked to first saccade onset during the 1 s viewing window (average across the 12,382 saccades that occurred in the same 5496 trials), revealing a saccadic‐spike potential (SP) tightly aligned to eye‐movement execution, followed by the P1‐N170 complex. The correspo [file PSYP-63-e70303-s001.zip › Figure S4.png]

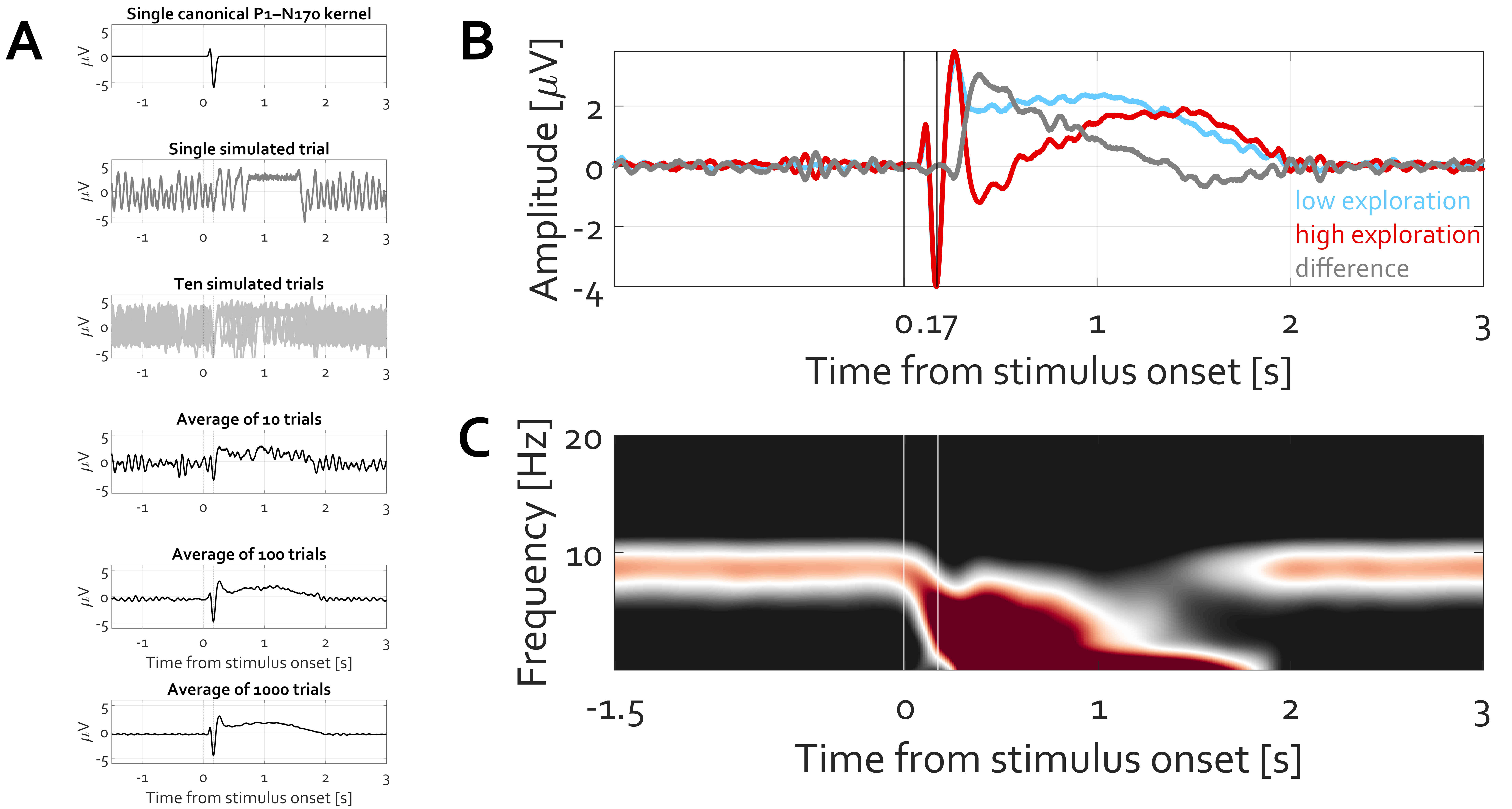

Supplement: Supplementary file 1 — Figure S1: Group‐level relationship between P1‐N170 timing and saccade onset latency. (A) Face viewing task; (B) IAPS picture viewing task. For both tasks, trials were sorted into bins according to the onset of the first saccade following stimulus onset within the 150–300 ms latency window (color‐coded from early to late saccade latencies). Left panels show stimulus‐locked ERPs averaged within each saccade‐latency bin (electrodes P7, P8, O1, O2 average reference montage), demonstrating a systematic shift in the timing of the P1‐N170 complex as a function of saccade onset latency. Right panels show the same data re‐aligned to saccade onset (saccade‐locked ERPs), revealing convergence of ERP waveforms across bins, with the negative deflection preceding saccade onset by approximately 100–150 ms. For both stimulus classes, this pattern replicates at the group level the single‐subject findings shown in Figure 5 (faces) and Figure 9 (IAPS) in the main text, albeit with reduced separation between bins due to fewer trials in the faces task than in the IAPS task and to lower trial counts for some participants. Figure S2: Stimulus‐locked and saccade‐locked ERPs reveal distinct temporal and topographic signatures of visual–oculomotor processing during passive viewing. Single participant data used in Figure 9 in the main paper. Note different y axes. (A) ERP time course locked to stimulus onset (averaged across 5496 trials), illustrating the canonical P1‐N170 complex followed by a sustained slow potential. The scalp topographies above and below the waveform depict the spatial distribution of the P1 and N170 components, respectively, highlighting their characteristic posterior‐occipital dominance. (B) ERP time course locked to first saccade onset during the 1 s viewing window (average across the 12,382 saccades that occurred in the same 5496 trials), revealing a saccadic‐spike potential (SP) tightly aligned to eye‐movement execution, followed by the P1‐N170 complex. The correspo [file PSYP-63-e70303-s001.zip › Figure S5.png]
